# Sex differences in alpha-synucleinopathies: a systematic review

**DOI:** 10.3389/fneur.2023.1204104

**Published:** 2023-07-20

**Authors:** Kausar Raheel, Gemma Deegan, Irene Di Giulio, Diana Cash, Katarina Ilic, Valentina Gnoni, K. Ray Chaudhuri, Panagis Drakatos, Rosalyn Moran, Ivana Rosenzweig

**Affiliations:** ^1^Sleep and Brain Plasticity Centre, Department of Neuroimaging, Institute of Psychiatry, Psychology and Neuroscience (IoPPN), King’s College London, London, United Kingdom; ^2^BRAIN, Imaging Centre, CNS, King’s College London, London, United Kingdom; ^3^School of Basic and Medical Biosciences, Faculty of Life Science and Medicine, King’s College London, London, United Kingdom; ^4^Department of Neuroimaging, Institute of Psychiatry, Psychology and Neuroscience (IoPPN), King’s College London, London, United Kingdom; ^5^Center for Neurodegenerative Diseases and the Aging Brain, University of Bari Aldo Moro, Lecce, Italy; ^6^Movement Disorders Unit, King’s College Hospital and Department of Clinical and Basic Neurosciences, Institute of Psychiatry, Psychology and Neuroscience and Parkinson Foundation Centre of Excellence, King’s College London, London, United Kingdom; ^7^Sleep Disorders Centre, Guy’s and St Thomas’ NHS Foundation Trust, London, United Kingdom

**Keywords:** alpha-synucleinopathies, sex differences, estrogen, Parkinson’s disease, Dementia with Lewy Bodies

## Abstract

**Background:**

Past research indicates a higher prevalence, incidence, and severe clinical manifestations of alpha-synucleinopathies in men, leading to a suggestion of neuroprotective properties of female sex hormones (especially estrogen). The potential pathomechanisms of any such effect on alpha-synucleinopathies, however, are far from understood. With that aim, we undertook to systematically review, and to critically assess, contemporary evidence on sex and gender differences in alpha-synucleinopathies using a bench-to-bedside approach.

**Methods:**

In this systematic review, studies investigating sex and gender differences in alpha-synucleinopathies (Rapid Eye Movement (REM) Behavior Disorder (RBD), Parkinson’s Disease (PD), Dementia with Lewy Bodies (DLB), Multiple System Atrophy (MSA)) from 2012 to 2022 were identified using electronic database searches of PubMed, Embase and Ovid.

**Results:**

One hundred sixty-two studies were included; 5 RBD, 6 MSA, 20 DLB and 131 PD studies. Overall, there is conclusive evidence to suggest sex-and gender-specific manifestation in demographics, biomarkers, genetics, clinical features, interventions, and quality of life in alpha-synucleinopathies. Only limited data exists on the effects of distinct sex hormones, with majority of studies concentrating on estrogen and its speculated neuroprotective effects.

**Conclusion:**

Future studies disentangling the underlying sex-specific mechanisms of alpha-synucleinopathies are urgently needed in order to enable novel sex-specific therapeutics.

## Highlights

Key findings

- There is conclusive evidence to suggest sex- and gender-specific differences in multiple aspects of alpha-synucleinopathies (i.e., genetics, demographics)
- The alpha-synucleinopathy process has a distinct motor and non-motor symptoms phenotype in men, compared to women.
- Gender, societal and lifestyle factors should be always considered when improving the quality of life and clinical management of patients suffering with alpha synucleinopathy.

What is known, and what is new?

- Male sex has been implicated as a predisposing factor toward developing alpha synucleinopathy.
- While there is evidence for the neuroprotective effects of female sex hormones, it is still unclear to what extent estrogen, or any other sex hormones, could be neuroprotective within the broad framework of alpha-synucleinopathies.

What is the implication, and what should change now?

- Addressing sex and gender differences in clinical and research settings has significant implications in improving diagnosis and management, implementing prevention strategies, and developing novel sex-specific health therapeutics.

## 1. Introduction

It has been more than twenty years ago since the discovery of the essential role of α-synuclein in the pathogenesis of Parkinson’s disease (PD) (1, 2). Since then, abnormal aggregates of α-synuclein, such as Lewy bodies and Lewy neurites, and glial cell inclusions, have been similarly linked with several other sporadic neurodegenerative diseases termed alpha-synucleinopathies [also please refer to (3, 4)]. The alpha-synucleinopathies, including idiopathic PD, Dementia with Lewy Bodies (DLB), Multiple systems atrophy (MSA), pure autonomic failure and REM sleep behavior disorder (RBD), have been also associated with synaptopathy and inflammation, as of yet poorly understood α-synuclein-related mechanisms, that likely contribute to the initiation and propagation of the disease (3). A body of work suggests that abnormal forms of α-synuclein may trigger selective and progressive neuronal death and dopaminergic transmission through mitochondrial impairment, lysosomal dysfunction, and alteration of calcium homeostasis not just in PD, but also in RBD, DLB and MSA (3). Alpha-synuclein aggregates perturb dopaminergic transmission and induce presynaptic and postsynaptic dysfunctions (5). Similarly, the presence of early inflammation in experimental models and PD patients, known to occur before deposition and spreading of α-synuclein, further supports a mechanistic link between inflammation and synaptic dysfunction (5).

All alpha-synucleinopathies appear to share synuclein-related neuroinflammation and many clinical, neurochemical and morphological features (3). Nonetheless, multiple clinical phenotypes exist for each of the three main α-synucleinopathies (PD, DLB and MSA), and a diverse dynamic distribution of their underlying neuropathologies has been demonstrated [also see (4, 5)]. For instance, in both PD and DLB α-synuclein inclusions are thought to be predominantly present in neurons and neurites (3, 4). However, while in PD their occurrence is associated with the loss of dopaminergic neurons in the substantia nigra, resulting in the prevalent motor symptoms; in DLB, it predominates in the neocortex with most prevalent symptoms being fluctuating cognition, recurrent visual hallucinations and spontaneous extrapyramidal motor features (5). On the other hand, in MSA the predominant presence of α-synuclein inclusions is thought to occur in the cytoplasm of oligodendrocytes, with selective neurodegeneration of the multiple brain areas resulting in parkinsonism, cerebellar ataxia and autonomic failure (4, 5). The understanding of these mechanisms is of pivotal importance to support the research on reliable biomarkers to identify the disease and possible disease-modifying therapies (3, 5).

In a similar vein, sex and gender differences have been a focus of interest in alpha-synucleinopathies in recent years, due to their potential to disentangle sex-specific disease phenotypes, and translate them to develop novel sex-specific therapeutics – known as a ‘bench-to-bedside’ approach (6–8). According to the Institute of Medicine’s Committee on Sex and Gender Differences, sex and gender differences are biological, physiological, and clinical differences between males and females that arise due to environmental factors and biological effects due to sex chromosomes and gonadal hormones (9).

Cumulative evidence has reported higher prevalence, incidence, increased disease severity and susceptibility of men compared with women in alpha-synucleinopathies such as PD (10), MSA (11, 12) and DLB (13), and even in the prodromal stage of alpha-synucleinopathies such as REM Behavior Disorder (RBD) (14). To address this, animal and clinical studies have posited the notion of neuroprotective properties of the female sex hormone estrogen against alpha-synucleinopathies (15–18). However, asserting any causality to estrogen as a protective factor in alpha-synucleinopathies remains speculative without a thorough investigation into the observable sex-and gender-specific differences. Hence, this systematic review aims to critically review the literature on sex differences in alpha-synucleinopathies, broadening our scope to sex-lineated assessments of prevalence, demographics, biomarkers, genetic factors, clinical features, neuroinflammatory and neurochemical responses, interventions, and quality of life themes. A comprehensive assessment of sex and gender differences in alpha-synucleinopathies holds promise for improving clinical diagnosis and developing treatments with optimal efficacy in both men and women.

## 2. Methods

### 2.1. Search strategies

**Figure 1:**
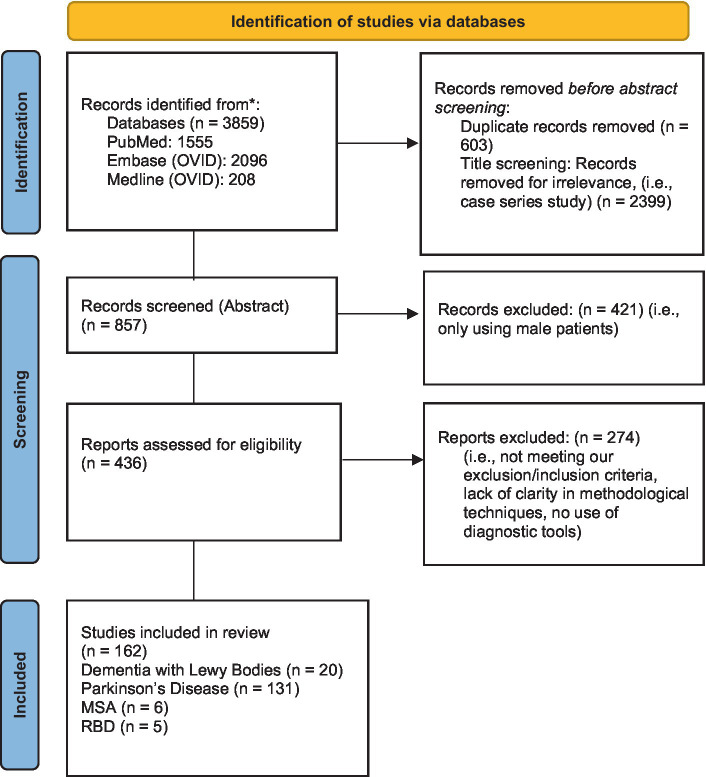
PRISMA 2020 Flow Diagram of study selection process.

This systematic review was conducted following the Preferred Reporting Items for Systematic Reviews and Meta-Analyzes (PRISMA) guidelines (19) (see Figure 1). Relevant studies were identified by two reviewers using the electronic databases of PubMed, Embase (Ovid) and Medline (Ovid). The following keywords were used: (sex OR gender differences) AND (alpha-synucleinopathies OR REM Behavior Disorder OR Parkinson’s disease OR Dementia with Lewy Bodies (DLB) OR multiple system atrophy (MSA)) (see Table 1). Eligible papers were extracted from 2012 until October 2022. The references of the selected articles were also examined to retrieve documents missed by the literature search.

**Table 1:** The search strategy and exclusion/inclusion criteria.

Database	Search strategy	Limits
PubMed	(Gender differences OR sex differences) AND (alpha synucleinopathies OR Parkinson’s disease OR Dementia with Lewy Bodies (DLB) OR Parkinson’s disease dementia (PDD) OR multiple system atrophy)	Year: 2012–2022 Species: Human Age: > 18 Only in English
Embase (Ovid)	(Sex differences) AND (Parkinson’s disease OR diffuse Lewy body disease OR multiple system atrophy OR Shy Drager syndrome)	Year: 2012–2022 Species: Human
Medline (Ovid)	(Sex characteristics) AND (Parkinson’s disease, Multiple System Atrophy or shy Drager syndrome, or alpha synucleinopathies)	Year: 2012–2022 Species: Human

### 2.2. Inclusion and exclusion criteria

Each article was first considered by title and abstract. This systematic review included: (1) original research articles; (2) only papers written in English; (3) observational, descriptive, longitudinal, retrospective, cross-sectional, or cohort studies; (4) meta-analyzes and systematic reviews that investigated sex differences in alpha-synucleinopathies; (5) human studies. Two reviewers (KR and GD) independently screened each eligible study, and disagreements were resolved through discussion after retrieving full text to determine whether inclusion and exclusion criteria were met (see Table 2). Please also refer to PICOS statement in Table 3.

**Table 2:** Search criteria.

	Exclusion criteria	Inclusion criteria
Manuscript characteristics	Conference abstracts and proceedings, unpublished data, preprints, government publications and reports, dissertations, and theses Animal studies Studies involving under 18 s, infants, pediatric Guidelines, statements, and comments General review papers	Original research articles Observational, descriptive, longitudinal, retrospective, cross-sectional, cohort, meta-analyzes, and systematic review studies that investigate sex differences in alpha-synucleinopathies Sample was well-described (e.g., number of subjects, recruitment criteria, age mean or age range etc)
Patients’ diagnosis	No use of any diagnostic tools	Clinical/probable diagnoses of alpha synucleinopathies Parkinson’s Disease (PD): Diagnosis of PD assessed using Unified Parkinson’s Disease Rating Scale (UPDRS) III or the United Kingdom Brain Bank criteria (41) or the International Classification of Diseases, 10th revision (ICD-10), or post-mortem, autopsy confirmation of PD pathology. Dementia with Lewy Bodies: Diagnosis made according to the international consensus criteria (42) or post-mortem, autopsy confirmation of DLB pathology. Multiple System Atrophy (MSA): Diagnosis made according to the Unified Multiple System Atrophy Rating Scale (UM-SARS) Part I and II (43) or post-mortem, autopsy confirmation of MSA pathology. REM Behavior Disorder (RBD): Diagnosed according to the International Classification of Sleep Disorders (ICSD) criteria (44) or polysomnography (PSG)
Study design	No comparison of male and female cohort	Case controlled study and/or with males and females’ comparison

**Table 3:** The PICOS statement.

Component of question	Example
Patient population	Alpha-synucleinopathies: Parkinson’s Disease, Dementia with Lewy Bodies, Multiple System Atrophy, REM Behavior disorder
Intervention	Medications, Surgical interventions
Control	Male and Female patients and/or healthy controls
Outcomes	Sex differences in PD, RBD, MSA and DLB
Study design	Retrospective, longitudinal, cross-sectional, observational, cohort studies, case–control studies, meta-analyzes, systematic reviews, randomized, controlled trials

### 2.3. Data extraction

**Figure 2:**
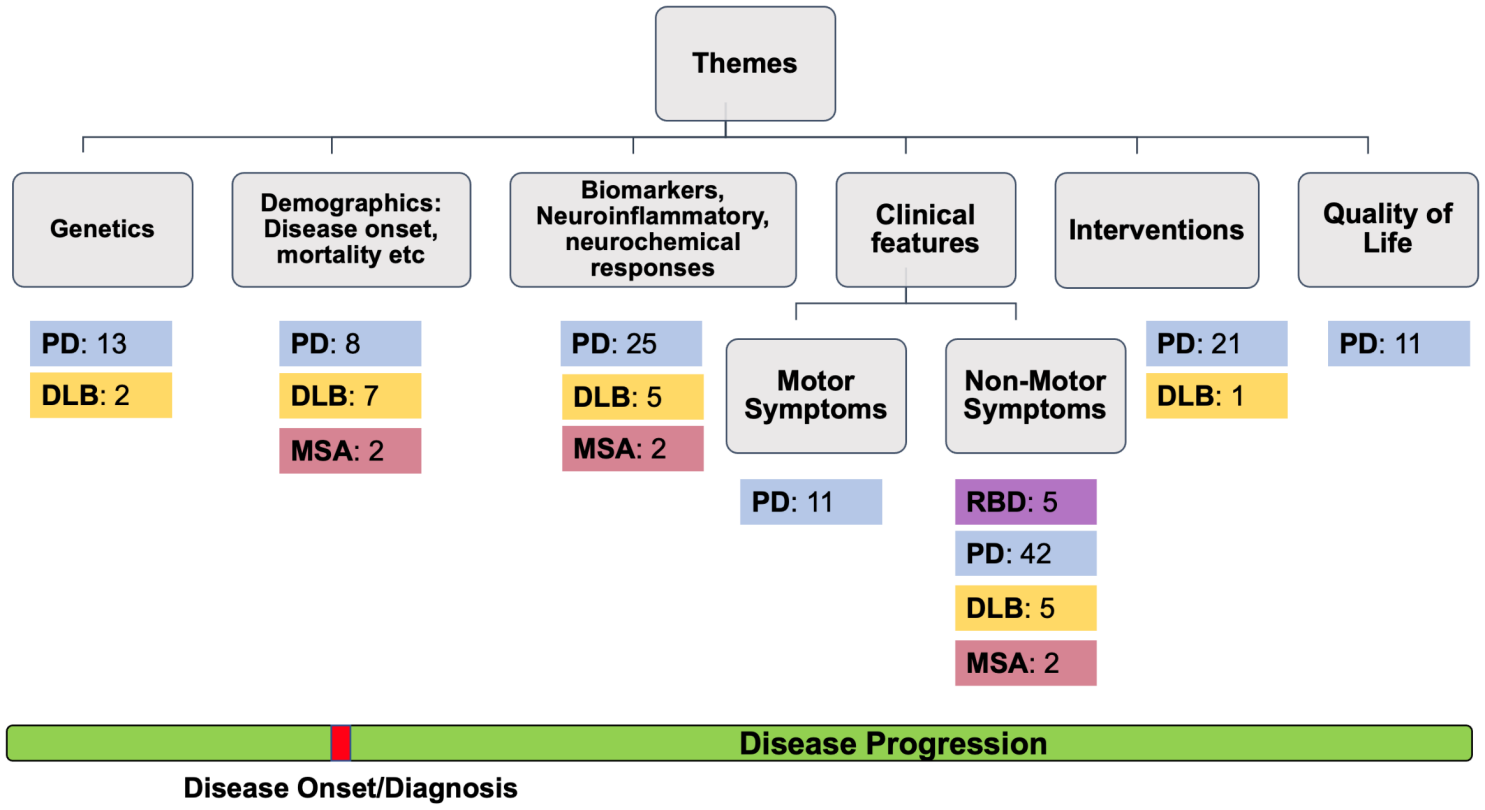
The number of studies found for REM Behavior Disorder (RBD), Parkinson’s Disease (PD), Dementia with Lewy Bodies (DLB) and Multiple System Atrophy (MSA).

For each article, two reviewers (KR and GD) independently extracted the following data: study name and year, the country, type of study, study aim, the subtype of alpha-synucleinopathy, sample size and age of male and female patients, the methods used, main findings and critical evaluation of the study. Then, the articles were classified and grouped according to the theme of the study (i.e., genetics, demographics, clinical features, interventions, or quality of life) (see Figure 2).

### 2.4. Quality assessment

Two reviewers (KR and GD) independently evaluated the quality of studies that were included using the two quality assessment scales: (1) Quality Assessment Tool for Quantitative Studies, developed by the Effective Public Health Practice Project (EPHPP)^1^ for observational, descriptive, longitudinal, cross-sectional, or cohort studies original research articles (20) and (2) A Measurement Tool to Assess Systematic Reviews-2 (AMSTAR-2) for meta-analyzes and systematic reviews (21). Any disagreements were resolved by discussion or by consulting with a senior reviewer. For the EPHPP scale, the following criteria were rated for each study on a scale of strong, moderate, or weak: selection bias, study design, blinding, data collection methods, confounders, and withdrawals/attrition (if any). Subsequently, these ratings were compiled to form a global rating: studies were rated as strong if they had no weak ratings, moderate if they had one weak rating, and weak if they received two or more weak ratings. As for systematic reviews and meta-analyzes, the AMSTAR-2 is a comprehensive critical appraisal tool focusing on weaknesses in multiple domains. AMSTAR-2 assesses 16 questions, among which 7 are critical domains (21) (Questions 2, 4, 7, 9, 11, 13, and 15; See Supplementary section). Subsequent evaluation is conceptualized into three options, “Yes,” “Partial Yes,” and “No.”

## 3. Results

### 3.1. Rapid eye movement behavior disorder

Rapid Eye Movement (REM) behavior disorder (RBD) is a parasomnia characterized by abnormal behaviors during REM sleep, accompanied by the loss of REM sleep muscle atonia and dream enactment (22–24). RBD can be categorized as either idiopathic RBD (iRBD) when not ascribable to other conditions or secondary RBD (sRBD) when associated with other neurological conditions or the use of certain medications (e.g., antidepressants) (25). Importantly, iRBD has been recognized as a prodromal stage in the development of alpha-synucleinopathies such as Parkinson’s disease (PD), Dementia with Lewy Bodies (DLB) and Multiple System Atrophy (MSA) (26–28). Sex differences demonstrated in RBD studies from 2012 to 2022 are summarized in Table 4.

**Table 4:** Sex differences in REM Behavior Disorder (RBD) studies from 2012 to 2022.

Author/year country type of study	Subtype	Sample size (age at time of study unless stated otherwise)	Methods	Main findings	Critical evaluation
Clinical features: non-motor symptoms; cognition					
Takeuchi et al. (32) Tokyo, Japan Retrospective, cross-sectional study	iRBD	*N* = 220 *M* = 141 (66.7 ± 6.7) *F* = 43 (68.7 ± 7.3)	Demographics and Clinical Assessments: Clinical interview with patient or bed partners RBDQ-JP ESS SST MoCA-J PSG: Video-PSG	Clinical/Demographics: Female iRBD patients had significantly later first symptom-witnessed age (e.g., sleep talking) No gender differences were found in the age of diagnosis, clinical severity, or olfactory or cognitive function PSG: No gender differences were found in the percentage of patients with motor events (simple/complex) and vocalization Phasic EMG activity was significantly higher in female patients, although no differences were found in tonic EMG activity Regarding neurodegenerative markers, no significant gender difference was found in the TDI score or proportion of patients with MCI	No inclusion of any other neurodegenerative markers, such as DAT scan or test for autonomic nervous symptoms No consideration of patients’ disease progression on symptoms manifestation EMG: phasic EMG activity was evaluated only on the chin, not the distal muscle of the arms Limitations of retrospective studies include recall bias
Castelnuovo et al. (33) Milan, Italy Retrospective, cross-sectional, clinical study	iRBD	*N* = 329 *M* = 280 (61.47 ± 6.66) *F* = 49 (64.88 ± 6.46)	Phonemic fluency 15 words test by Rey Raven’s Progressive Matrix Alternative matrix MMSE PSG	Significant gender differences in RBD-onset age No patients showed a cognitive impairment Females scored significantly better in tests that assess phonemic fluency (*p* = 0.014) and long-term verbal memory in learning (p = 0.012) and in false positive components (*p* < 0.001) Males performed significantly better in tests that assess nonverbal reasoning (*p* = 0.04) and visual selective attention (*p* = 0.046)	No inclusion of any other neurodegenerative markers, such as DAT scan or test for autonomic nervous symptoms No consideration of patients’ disease progression on symptoms manifestation Limitations of retrospective studies include recall bias
Clinical features: non-motor symptoms; sleep					
Bugalho and Salavisa (38) Lisbon, Portugal Retrospective, cross-sectional, study	iRBD sRBD	*M* = 40 (71.13 ± 9.87) *F* = 17 (71.69 ± 10.62) IRBD: M = 18\u00B0F = 4 sRBD: PD = 23 DLB = 11 MSA = 1 M = 22, *F* = 13	Clinical history and demographic information were obtained RBD-SQ Video-PSG REM Sleep Motor Event Assessment: Quantification of motor events according to type (myoclonic versus simple etc.)	The relation between sex and diagnostic category was nonsignificant, although there was a tendency for a higher frequency of iRBD in the male group	Small sample size Certain demographic information is not available (i.e., bedpartner information) EMG of the upper extremities was not available – missed patients with RBD
Fernández-Arcos et al. (45) Barcelona, Spain Retrospective, cross-sectional, longitudinal study	iRBD	*N* = 203 *M* = 162 (age at diagnosis = 68.6 ± 6.1) *F* = 41 (age at diagnosis = 68.8 ± 6.7)	Demographics and Clinical Assessments: Clinical history and demographics information were obtained (i.e., medication history) Sleep habits, dream recall and its content, self-awareness and characteristics of abnormal motor and vocal behaviors during sleep, resulting in injuries during sleep and overall subjective sleep quality Video-PSG	Clinical/Demographics: No significant differences were found for age of iRBD diagnosis, RBD duration and follow-up duration between males and females Dream Content: Males displayed more frequently aggressive behavior (e.g., punching, assaulting bed partner) and vocalizations (e.g., swearing), recalled more violent and action-filled dreams (E.g., flights, arguments) and were more likely to have a bed partner Females dreamed more commonly about children in life-threatening situations and had depression more commonly	Gender differences in PSG findings not mentioned Retrospective study: recall bias and complete information were not available in some instances Dream content was assessed *via* semi-structured interviews and not systematic analyzes
Zhou et al. (14) Sichuan, China Cross-sectional, clinical study	iRBD sRBD	*N* = 90 *M* = 63 (age at onset = 56.2 ± 14.1) *F* = 27 (age at onset = 45.3 ± 19.3)	Demographics and Clinical Assessments: Clinical interview to obtain demographics (i.e., disease duration, associated comorbidities and use of medications) RBDQ-HK Questionnaire Video-PSG: Quantification of EMG activity	Clinical/Demographics: Females were significantly younger than males in the mean age of RBD onset and mean age at diagnosis. Secondary RBD is significantly higher in females Antidepressant use more common in females PSG: No gender differences in the quantification of EMG activity during REM sleep Females spent significantly more time in SWS and less stage 1 time than males Behaviors during sleep in females were fewer than in males, although no gender differences were found in phasic or tonic activities Dream Enactment/ Content: No significant gender differences in dream content, although: Females have less dream-enacting behaviors, especially in movement-related dreams and falling out of bed	The first study to quantify EMG activity in males and females with RBD

DLB, Dementia with Lewy Bodies; EMG, Electromyography; ESS, Epworth Sleepiness Scale; F, Female sample; iRBD, idiopathic RBD; M, Male sample; MMSE, Mini-Mental State Exam; MoCA-J, Japanese version of Montreal Cognitive Assessment; MSA, Multiple System Atrophy; N, Total number of sample; PD, Parkinson’s Disease; PLM index, Periodic Limb Movement index; PSG, Polysomnography, REM, Rapid Eye Movement; RBD, REM Sleep Behavior Disorder; RBDQ-JP, Japanese version of RBD Questionnaire; RBDQ-HK, Hong Kong version of RBD questionnaire; sRBD, secondary RBD; SST, Sniffin’ Sticks Test.

RBD has long been considered a male-dominant parasomnia, with more than 80% of patients being male (29–31). Additionally, women with RBD were reported to have a significantly later age onset of iRBD than men with RBD (32, 33). However, when sRBD patients were included, females make up a higher proportion of early-onset RBD patients than males (14). This latter result corroborates findings from previous studies that found a greater proportion of females in early onset RBD, as compared to the late-onset groups, predominantly due to secondary factors such as narcolepsy and antidepressant use (34–37).

Apparent sex differences in clinical presentation and polysomnography (PSG) findings have also been reported (32, 33, 38) (Table 4). In sleep architecture, sex differences in time spent in different sleep stages and electromyography (EMG) activity were found (14, 32, 33, 38). More specifically, sleep stage N1 percentage was significantly higher in males with RBD than in females with RBD (11.96 ± 7.32 vs. 9.60 ± 6.23, *p* = 0.047; 19.9 ± 13.1 vs. 12.1 ± 10.8, *p* = 0.028) [14, 33], while REM latency (132.03 ± 76.37 vs. 108.86 ± 69.99, *p* = 0.049) and slow wave sleep latency (9.3 ± 7.9 vs. 13.1 ± 6.0, *p* = 0.032) were significantly higher in females with RBD (14, 33). This could be due to the effects of female hormones on sleep architecture (39, 40), as female adults tend to engage in more deep sleep than males. It is also worth noting that slow wave sleep decreases with age, and in sRBD, younger age could explain the longer deep sleep in females with RBD (14, 40).

With regards to EMG activity, significantly higher phasic EMG activity was reported in females with RBD compared to males with RBD (*p* = 0.009), although no sex differences were found in the percentage of RBD patients with motor events (simple/complex) and vocalization (32). In contrast, Bugalho and Salavisa demonstrated a significantly higher phasic muscle activity index and relative number of myoclonic and trunk movements in males with RBD compared to females with RBD (*p* = 0.005) (38). This is supported by the fact that the periodic limb movements (PLM) index was significantly higher in males with RBD compared to females (*p* < 0.001) (33). However, Zhou et al. did not find any significant sex differences in phasic (*p* = 0.466) or tonic (*p* = 0.988) EMG quantification (14). These conflicting findings could be due to methodological discrepancies in stratifying for disease severity, stage of RBD and age onset.

Men with RBD were also more likely to exhibit violent and aggressive behavior (otherwise incongruous to their premorbid personality), while women with RBD experienced less dream-enacting behavior (14, 33, 45). Fernández-Arcos et al. reported that men with RBD displayed significantly more aggressive behavior [e.g., punching, assaulting bed partner, vocalizations (swearing)] and increased recall of violent, action-filled dreams, while females with RBD dreamt more about children in life-threatening situations (45). With the inclusion of sRBD patients, women with RBD also displayed significantly less dream-enacting behaviors, especially in movement-related dreams and falling out of bed (14).

Several biological and societal factors could explain the male predominance of RBD. Firstly, sex hormones (i.e., estrogen, androgens) may mediate the distinct phenotypical presentation of RBD (46, 47). Notwithstanding this, in a study conducted on men with RBD and healthy controls, no differences in serum sex hormone levels were found, suggesting that androgenic abnormalities may not account for this male predominance (46). More specifically, in this study, serum levels of total testosterone, calculated free testosterone, calculated bioavailable testosterone, luteinizing hormone, follicle stimulating hormone, estradiol-17 beta, sex-hormone binding globulin, and prolactin were not found different between male idiopathic RBD patients and healthy male controls (46). On the other hand, some evidence seems to point to the neuroprotective effect of estrogen against neurodegeneration in the nigrostriatal regions, although this remains obscure (48). Furthermore, on a more behavioral level, women with RBD tend to experience less disruptive behavior. This might make them less likely to seek medical consultation (49, 50). Additionally, RBD occurrence in females might also be underreported, predominantly due to the inadequacy of questionnaires for detecting female sleep behaviors (37).

### 3.2. Parkinson’s disease

*Demographics, epidemiology, and prevalence:* Parkinson’s Disease (PD) is the second most common neurodegenerative disorder associated with multiple neuropathological hallmarks, including neuronal loss in the substantia nigra (51). Consequently, patients with PD (PwP) typically display a range of motor and non-motor symptoms, including cognitive impairment, dementia, and motor dysfunction (52, 53) (please see Supplementary Table S1 for further details). Across prevalence, incidence, and mortality studies in PD, two trends emerged; (1) Higher incidence, prevalence, and mortality rate were consistently reported in male PwP, and (2) male-to-female incidence ratio across age groups were not constant; instead, it strikingly increases with age, and this was observed across different countries (54–61).

In a French nationwide study and meta-analysis, Moisan et al. reported that the prevalence and incidence of male-to-female ratio increased by 0.05 and 0.14 per decade, respectively, with incidence increasing over 1.6 (*p* < 0.001) times higher in male PwP, in age group over 80 years (59). When geographical locations are considered, Pringsheim et al. also showed a significantly higher prevalence of PD in males, particularly in Western countries and South America (60). However, when parsed by age groups, a significantly higher sex ratio PD prevalence was reported only in the younger age group 50 to 59 (PD prevalence of 41/100000 in females and 134/100000 in males; *p* < 0.05) (60). However, in a Norwegian study, Brakedal et al. did not observe an age-dependent change in male-to-female ratio of PD prevalence, which remained at approximately 1.5 across all age groups (54). Surprisingly, when adjusted for sex-specific mortality of the general population, mortality among female PwP was equal to or higher than mortality in male PwP (54). These findings also did not support previous mortality studies in which a higher mortality rate was consistently reported in male PwP (55, 58).

For example, an Italian mortality study conducted from 1980 to 2015 reported that male PwP have higher mortality, as compared to female PwP (Annual Mortality Rate (AMR)/100,000: 9.0 in males, 5.25 in females) (55). Similarly, PwP with dementia and male PwP had a higher mortality risk of 3.78-fold and 2.05-fold, respectively (58). Indeed, the male sex remains a significant predictor of mortality and survival predominantly due to increased disease severity in multiple domains, including cognition, postural instability, and a higher prevalence of dementia (56–58, 61).

*Genetics:* Mutations in Leucine-Rich Repeat Kinase 2 (LRRK2) and Glucosidase Beta Acid (GBA) have often been considered the most common genetic cause of monogenic and sporadic forms of PD (62–66). Several studies have posited a higher prevalence of LRRK2 PD mutations in female PwP (67–69). In a meta-analysis that included 66 studies, Shu et al. parsed clinical heterogeneity among four LRRK2 variants in PD (G2019S, G2385R, R1628P and R1441G) and confirmed the association of female sex to G2019S. Interestingly, PwP with G2019S were more likely to have high University of Pennsylvania Smell Identification Test (UPSIT) scores (*p* = 0.01) and good response to levodopa (*p* < 0.0001) (68). Other variants of the LRRK2 mutation, such as G2385R, also displayed sex-related phenotypes differences, with male carriers of G2385R having a lower risk of cognitive impairments (*p* = 0.003) and female G2385R carriers displaying a lower risk of autonomic dysfunction (*p* = 0.04) (70). Crucially, these findings emphasize genetics’ key role in driving sex-specific phenotypical differences. Conversely, the GBA gene encodes for the lysosomal enzyme glucocerebrosidase known to maintain glycosphingolipid homeostasis (71). It has been suggested that up to 15% of PD patients may have mutations in the GBA gene, making it one of the most important genetic risk factor for PD (71). Clinically, GBA-associated PD may have an earlier age at onset, common cognitive impairment and more rapid progression (72, 73). Despite its importance, the relationship of sex and GBA mutation remains unclear to date.

Genes related to mitochondrial functions have also been identified to exhibit a sex-specific protective mechanism (74–76). For example, mitochondrial haplogroup U demonstrated a significant protective effect in female PwP of the Cypriot population (74); mutations on mitochondrial DNA (51782A) were lower in male PwP, particularly in younger age groups and provided a protective effect on longevity in Chinese Han, Uygur, and Japanese populations (76–78) while, variants of mitochondrial transcription factor A (TFAM) increase the risk of PD in males (75).

**Figure 3:**
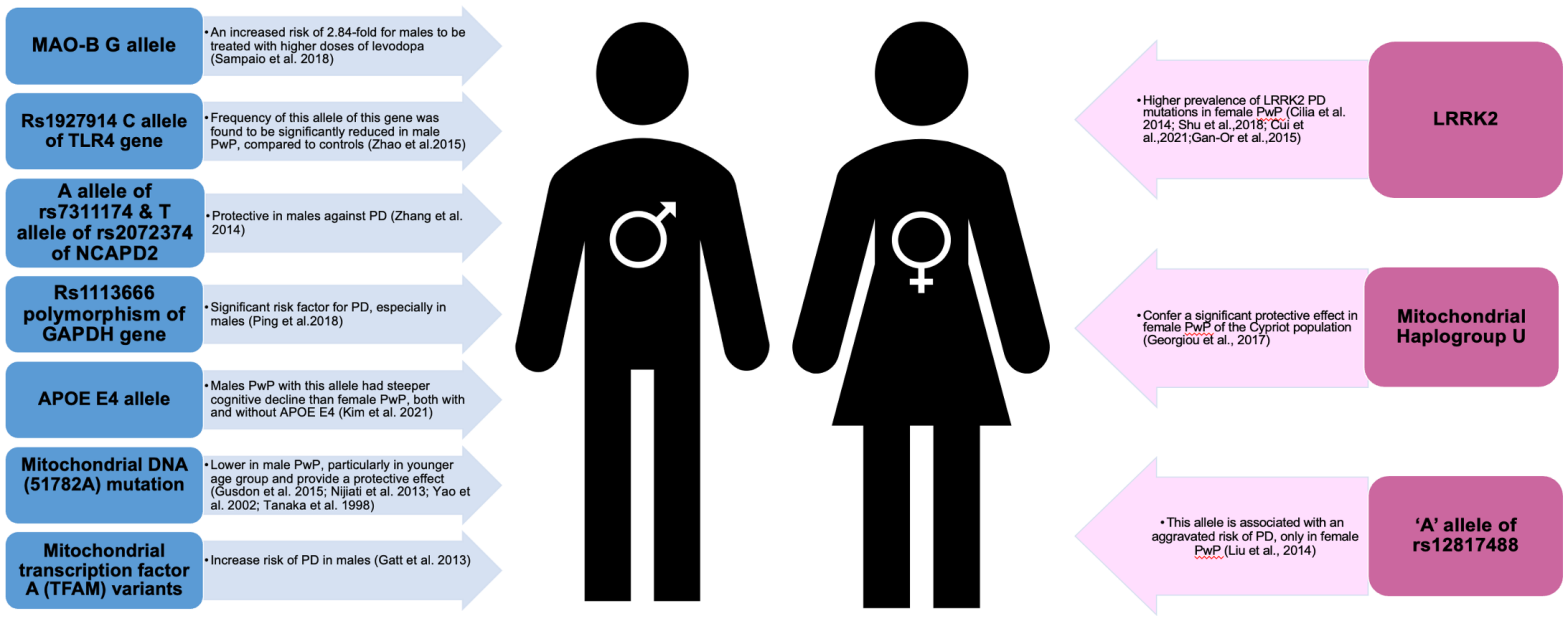
Schematic presentation of genes implicated in male and female patients with Parkinson’s disease.

Other genes involved in immunological and inflammatory responses, estrogen regulation, dopamine modulation and chromosome condensation were similarly to affect (either direction) the pathogenesis of PD, especially in male PwP (79–82) (See Figure 3). For example, male PwP carriers of MAO-B G allele had a 2.84-fold increased risk of being treated with higher doses of levodopa (79); rs1113666 polymorphism of GAPDH gene was found to be a significant risk factor for PD, especially in male PwP (81); and A allele of rs7311174 and T allele of rs2072374 was reported to be protective in males (82). On top of this, male PwP with the APOE4 allele had steeper cognitive decline than female PwP groups, both with and without APOE4 (83), while association of rs12817488 with PD was reported only in females PwP (84), further reiterating the need to consider genes and sex differences in PD.

*Biomarkers:* Low uric acid (UA) levels have been consistently linked with an increased risk of PD and increased disease severity, particularly in male PwP (85–91). However, controversial findings were obtained when analyzes were stratified by age and estrogen levels (92). Notably, Cortese et al. showed a significant association between exposure to urate-lowering drugs in reducing PD risk in females in a higher age group (>70 years old) when there were higher UA levels premenopausally, but not in males (92). Based on these findings, there seems to be a sex-dependent predisposition of uric acid on nigrostriatal dopaminergic neurons and estrogen, which may confer beneficial neuroprotective properties in females, although further analyzes are warranted.

Another potential sex-specific biomarker for PD progression is serum homocysteine (93, 94). Elevated homocysteine levels displayed a sex-specific profiling of PD (93, 94). For instance, a positive association of elevated homocysteine with motor impairments (Unified PD Rating Scale (UPDRS)-III) in only male PwP (*p* < 0.001) and a negative association of elevated homocysteine with cognition only in female PwP (*p* = 0.021), further reiterating the distinct phenotypical sex-specific profiles of PD (93).

Metabolites and lipoproteins could also serve as sensitive biomarkers in identifying sex-specific profiles of PD (95–99). In lipid profiling studies, there is a mutual agreement on sex-specific lipid profiling and functioning in cognitive manifestations of PD (96, 97). For example, in female PwP, a positive association between hypertriglyceridemia and cognitive performance on the Frontal Assessment Battery (FAB) task was found (*p* = 0.013) and a negative correlation between triglyceride serum levels and cognitive performance on FAB task (*p* = 0.005) (96). However, in male PwP, a negative association was found between hypercholesterolemia and normal FAB performance and between high low-density lipoprotein cholesterol levels and FAB score (*p* = 0.027) (96), suggesting a differential functional role of lipids in sex-specific phenotype presentation of symptoms.

Biomarkers, such as alpha-synuclein, DJ-1 protein, and serum brain-derived neurotrophic factor (BDNF) levels, have also been expressed differently between sexes (100–102). Immunoenzymatic analyzes revealed lower plasma alpha-synuclein concentration levels in severe PD stages only in male PwP (100). This association is in line with more severe cognitive impairments, hallucinations, and sleep disorders, experienced by male PwP (100). Furthemore, DJ-1 protein levels was reported to be significantly higher by 1.7-fold in male PwP than male controls, suggesting a clear sex-specific biomarker of PD (101). In females, on the other hand, decreased BDNF levels were reported to be associated with females only among depressed PD patients, suggesting a sex-specific expression of biomarker and symptom profiling (102).

Sex differences in the expression of gut microbiome and immunological biomarkers have also been identified (103–105). In the first-ever metabolites profiling study using nuclear magnetic resonance (NMR), Baldini et al. analyzed 129 microbial metabolites through personalized metabolic modeling using microbiome data and genome-scale metabolic reconstructions of human gut microbes (103). The reported PD-associated microbial patterns were statistically dependent on sex, with *Paraprevotella genera* (a genus of bacteria) significantly influenced in female PwP (103). This was the first study to portray sex differences in the microbiome environment in PD, which supports the association of the gut-brain axis in immune response. Other analyzes of immune biomarkers in the stools of PD patients also reported a disease-related increase in numerous immune and angiogenesis mediators, only in stools of female PwP (106). This needs further research as monocyte response and phagocytic markers in PD have been reported to exhibit distinct sex-specific expression (104, 105, 107).

#### 3.2.1. Clinical features

##### 3.2.1.1. Motor symptoms

There is a general trend for severe motor impairment in male PwP than in females (108, 109). This is accompanied by an altered pattern of functional networks (e.g., sensorimotor networks), abnormal motor cortex measurements and lower dopaminergic binding in male PwP (110–112). In a recent study, Boccalini et al. investigated dopaminergic dysfunction according to PD-stratified clinical subtypes of motor function (i.e., mild, intermediate, or diffuse-malignant) in *de novo* PD patients using the Parkinson’s Progression Markers Initiative (PPMI) database (108). In mild motor and intermediate subtypes, they found that male PwP exhibited poorer cognitive performance than females, and those with motor impairments had lower dopamine binding in the putamen with more severe widespread connectivity alterations in the nigrostriatal dopaminergic neurons than female PwP (108). This dysfunction was also observed on a behavioral level (113, 114). For instance, in a 5-year longitudinal study, Picillo et al. reported that male PwP experienced a significantly higher longitudinal decline in self-reported motor symptoms, with a yearly increase in UPDRS-II by 0.57 relative to females (1.27 vs. 0.7, *p* < 0.001) (113).

Nonetheless, the findings of several studies suggest a more complex relationship between female hormones and motor symptoms in PD (115). For instance, in a study on female PwP, younger age of onset and higher Hoehn and Yahr (H&Y) stage were identified as risk factors of wearing off phenomenon, while younger onset age was associated with dyskinesia (115). Moreover, female PwP with wearing-off phenomenon and dyskinesia were shown to have higher levels of prolactin (115). It has been hypothesized that in some patients age onset and disease severity might override the neuroprotective benefits of female hormones.

Furthermore, motor symptoms tend to emerge later in female PwP and display a sex-specific phenotypical motor presentation (116, 117). Female PwP were more likely to experience reduced rigidity (116), tremor (117), and levodopa-induced dyskinesias (115, 118), while male PwP were reported to be more susceptible to later development of freezing of gait (119), and camptocormia (abnormal severe forward flexion of the trunk) (120).

##### 3.2.1.2. Non-motor symptoms

Non-motor symptoms (NMS) consist of a wide range of symptomology spectrum and severity, such as cognitive deficits, sexual and urinary dysfunction, sleep, mood disorders and psychosis and odor discrimination (8, 53, 72, 113, 121–150). Despite methodological differences due to different screening tools being adopted, two trends emerged, (1) male PwP were more likely to experience severe non-motor symptoms, particularly in cognition, olfaction, sleep, speech problems, impulse control disorders (i.e., pathological gambling and hypersexuality), dementia, urinary and sexual dysfunction (113, 121, 125–127, 129, 131, 132, 136, 138–144, 151, 152) (2) female PwP were more likely to experience fatigue, higher pain levels, and psychosis and mood disorders (i.e., depression (153), anxiety) and impulse control disorders (i.e., binge eating and compulsive buying) (131–133, 139, 145–147, 151, 154–156).

The correlates of cognitive sex differences in healthy, neurotypical people remain poorly understood (157). It is thought that many biological and psychosocial factors act to modulate these cognitive abilities leading to mixed results in the scientific literature (157). Nonetheless, numerous studies have suggested that male sex may be a dominating risk factor for dementia and cognitive impairment (53, 121, 126, 142–144). In keeping, male PwP have been shown to develop a more rapid and severe cognitive decline by comparison to female PwP (53, 72, 113, 121). In a recent 5-year longitudinal study in *de novo* PD population, male PwP experienced a steeper decline in both motor (*p* = 0.009) and non-motor (*p* = 0.009) symptoms, with a yearly increase in self-assessed UPDRS I by a multiplicative factor of 0.98, as compared to 0.67 in female PwP (113). Sex differences were also noted in differential phenotypes of deficits in executive functioning (53, 121, 122, 126, 148, 149). Both healthy males and male PwP groups performed significantly worse than females in semantic verbal fluency and delayed recall, while healthy females and female PwP groups performed worse in visuospatial function (126).

Clear sex differences in sleep have also been reported (127, 128, 130). Male PwP were more likely to experience increased daytime sleepiness, higher motor impairment and lower mini-mental score in tandem with abnormal sleep-related motor-behavioral episodes (127, 128, 130). In line with this, RBD and PD studies have also shown that male PwP have a higher prevalence of RBD and display greater global cortical and subcortical gray matter atrophy even when compared with females in PD-RBD group (125, 126, 143, 158). This suggests distinct sex-specific heterogenous profiling of RBD and other sleep parameters in PD.

Across studies using different cohorts’ groups, male PwP consistently presented with more prominent sexual and urinary dysfunction than females (8, 131, 150). For instance, Martinez-Martin et al. reported a lower prevalence of sexual dysfunction in female PwP (~28%) as compared to males (~50%). This could be due to distinct biological features between sexes (8). The autonomic nervous system itself is sexually dimorphic with differences in urinary tracts (159, 160), brain anatomy (161, 162), and genital system (163).

Female PwP, on the other hand, have been reported to have a higher prevalence of mood disorders such as anxiety, depression and apathy, as well as to have a heightened experience of fatigue and pain (130–133, 139, 145–147, 154, 155). Zhu et al. reported higher scores on the Hamilton Rating Scale for Depression (HAMD) domains of anxiety/somatization, and hopelessness in female PwP (154), perhaps indicative of the functional role of estrogen in mood regulation (164). Specifically, affective regulation has been linked to neural structures rich in estrogen receptors and estrogenic regulation of neurotransmitters. Interestingly, even in healthy women, studies have reported a higher incidence of depression (165, 166) and anxiety (167) during peri/menopause – a period of drastic reduction in estrogen levels, which have been reported to coincide with the onset of PD (168, 169). Conversely, it has been shown that hormone therapy may prevent mood disorders during this period, and while the exact mechanism remains unknown, there is compelling evidence that supports neuromodulatory and neuroprotective effects of estrogen, which are directly relevant to mood symptomatology (164). In future, it would be important to elucidate the nature of postmenopausal exogenous hormone formulations in relation to premenopausal endogenous levels, as well as the ratio of estrone to estradiol, all of which warrants urgent consideration to address these debilitating non-motor symptoms in female PwP during the peri/menopause (164).

Moreover, impulse control disorders in PD, described as aberrant behaviors such as pathological gambling, hypersexuality, binge eating, and compulsive buying, which typically occur as a result of dopaminergic therapy, have all variably been shown to sport variable phenotypic sex-related expressions, e.g., with pathological gambling and hypersexuality more prevalent in men, whereas binge eating and compulsive buying occur more frequently in women (170). In that background, and given that specific impulse control disorders share clinical, phenomenological and biological features with obsessive–compulsive disorder (171), it is of note that sexually dimorphic pattern of genetic susceptibility to OCD’s clinical heterogeneity has been recently demonstrated, potentially requiring different specific therapeutic strategies (172). Further research is warranted to validate sex as one of the important determinants of the heterogeneity of impulse control disorders in PwP.

Pain is also more frequently reported in female PwP (133, 146, 173, 174). The mechanisms underlying this, and other mood phenomena, remain unclear. Arguably, however, they may reflect differential effect of the alpha-synucleinopathy process on distinct pain/mood centers in the female brain. For instance, one of the neuroanatomical candidates may be the dysfunction of the circuitry involving the posterior bed nucleus of the stria terminalis (BNST). The BNST is the center of the psychogenic circuit from the hippocampus to the paraventricular nucleus, this circuit is important in the stimulation of the hypothalamic–pituitary–adrenal axis, and its dysregulation may lead to mood, pain and anxiety disorders, social dysfunction and psychological trauma (175). It is known that oestradiol exerts its effects in the canonical pathway through the transcription factor estrogen receptor-α, the neuronal targets of which include the BNST for a review see (176). The BNST, is a sexually dimorphic structure, commonly approximately 1.5–2 times larger in men, compared to women (176). Of note, atrophy of the BNST has been demonstrated in *de novo* PD (177), possibly suggesting that in women, who have smaller BNST, any such neurodegenerative process may have proportionally larger negative impact on affective processing of pain.

#### 3.2.2. Interventions

##### 3.2.2.1. Pharmacological

One commonly used first-line PD treatment is levodopa (178). Several patterns were observed in levodopa pharmacokinetics and treatment outcome between sexes (79, 173, 178–180). Female PwP were more susceptible (“brittle response”) to levodopa-induced dyskinesia and wearing-off phenomenon (115, 118, 173, 181, 182). Studies into intra-and inter-individual variability in levodopa’s pharmacokinetics (PK) reported sex-specific treatment responses (178). Conti et al. measured plasma levodopa concentrations and pharmacokinetic parameters (Area under curve (AUC), Maximum plasma concentration (Cmax), time to reach Cmax (Tmax), half-life (t1/2)) in levodopa-naïve and levodopa-treated PD patients (178). Interestingly, AUC and Cmax were significantly higher in female PwP than in males, with body mass index (BMI) significantly predicting t1/2 only in female PwP (*p* = 0.027) (178). It is worth noting that in this study, female PwP had a longer duration of disease (59 ± 24.5 months) than male PwP (34 ± 28.5 months).

UA-level modification may also offer a tailored sex-specific PD treatment (183). Previous studies consistently reported the association of lower serum UA with higher disease severity, particularly in male PwP (85–88). This sex-specific profiling of UA also extends to urate-altering drugs (183). Schwarzschild et al. conducted a randomized, double-blinded clinical trial of the Safety Urate Elevation in PD (SURE-PD) trial and found that inosine elicited higher levels of serum urate that were 50% greater in female PwP (3.0 mg/dL) than in male PwP (2.0 mg/dL). CSF urate was also significantly higher on mild (+87%, *p* < 0.001) or moderate (+98%, *p* < 0.001) inosine than placebo, only in female PwP (183). Regarding motor severity, slower UPDRS progression was related to an increase in serum urate (*p* = 0.001) and plasma antioxidant capacity (*p* = 0.006). No relationship was found in male PwP, suggesting a protective effect of underlying female sex steroids interplay with urate (183).

Targeting a non-dopaminergic system may be effective in ameliorating motor and non-motor fluctuations that arise when on levodopa (184). One such treatment is safinamide (185). Safinamide acts on the reversible inhibition of the monoamine oxidase-B (MAO-B) enzyme and modulation of excessive glutamate release (186). In a recent study on the efficacy of safinamide on PwP, Pellechia et al. reported improvements in the total UPDRS score were 43.5% in males versus 39.1% in female PwP (185), further providing support for sex-specific treatment response in PD.

*Surgical:* Deep brain stimulation (DBS), a neurosurgical procedure that involves electrical stimulation of the global pallidus internus (GPi) or subthalamic nucleus (STN), is an alternative treatment for PD, particularly in advanced PD (187). In terms of sex disparities and treatment outcomes, three trends emerged: (1) sex disparities in DBS selection, particularly in the undertreatment, referral and follow-ups of female PwP, (2) similar surgical outcomes postoperatively after DBS between sexes, although males were more likely to display lasting improvements and (3) quality of life postoperatively depend on sex-specific symptoms phenotype (187–193).

Gender-specific disparities in treatment accessibility and patients’ behavioral approach to mitigating PD symptoms are a primary concern, particularly for healthcare professionals (187, 190). In a cross-sectional, pseudo-randomized study in the United Kingdom, female PwP were disproportionally underrepresented in referral compared to the general PD population (*p* = 0.002), although they were more likely to be approved for DBS than males (*p* = 0.029) (187). Furthermore, female PwP were less likely to undergo DBS due to their preference (*p* < 0.001), while male PwP were more likely to be lost to follow-up (*p* = 0.046) (190). In terms of behavioral approach, female PwP were more likely to express strong fear of complications and were more likely to consult with immediate family members prior to deciding on DBS (194).

Although there was no sex differences in postsurgical outcomes improvements right after DBS (187, 190, 195), in subsequent follow-ups, female PwP showed a trend toward worsening in bradykinesia after 1 year and a lower score in non-dopaminergic features after 10 years (196). Furthermore, a recent study has also identified male sex as a significant predictor of DBS-induced improvement in camptocormia and global postural angle (193). Despite that, interestingly, in a mortality study assessing PwP treated with DBS, only male sex and disease duration were significant predictors of mortality (197).

Another controversial aspect of gender disparities after DBS is the quality of life in PwP (198, 199). While the long-term effect and short-term effect of DBS are similar in cognitive function and depressive symptoms, at 5-year follow-up post-DBS, physical quality of life is significantly more improved only in male PwP (*p* < 0.001) but not in female PwP (*p* = 0.409) (198). Despite that, there are also reports that suggest that female PwP experience greater improvements in activities of daily life (ADL) and positive effects on mobility, stigma and cognition than males (199).

##### 3.2.2.2. Quality of life

Despite the higher prevalence and disease severity in male PwP, there seems to be a trend of lower quality of life in multiple aspects of female PwP (156, 174, 200–206). This could be attributable to several gender, societal factors and the nature of clinical manifestation that contribute to lower quality of life in female PwP (174, 200, 207). In an Israeli study, lower quality of life in female PwP was attributable to the higher prevalence of depression and pain, while male PwP’s quality of life only worsened in advanced stages (174, 208). These findings align with studies conducted worldwide, in which severe anxiety, lower nutritional status, lower emotional well-being, higher stigma, and psychosocial functioning were the most robust features of poorer quality of life in female PwP (201–203, 205, 207). This suggests that societal expectations of gender role factors are crucial in disease management and interventions in PD.

Furthermore, other environmental factors such as living conditions and visitation/seeking-care behavior could also account for lower quality of life in female PwP (201, 209). Female PwP were more likely to live alone (18% had no caregivers, compared to 2.4% of males) (201). Even if they utilized care services, female PwP were more likely to use home health and nursing facility care more often. They had less outpatient physician contact than male PwP throughout PD (204). For effective delivery of treatment, these societal expectations and gender patterns of seeking help should be considered by clinicians.

### 3.3. Dementia with Lewy Bodies

Dementia with Lewy Bodies (DLB) is the second most common neurodegenerative dementia among the elderly (210). Core clinical features of DLB include neuropsychiatric symptoms (i.e., visual/auditory hallucinations), parkinsonism, and cognitive impairments (i.e., deficits in memory and executive functions) (211). On a pathological level, DLB is characterized by the presence of Lewy bodies (i.e., neuronal inclusions of alpha-synuclein) with differing degrees of co-existing Alzheimer’s disease (AD)-related pathology (i.e., amyloid plaques and neurofibrillary tangles (NFT)) (212, 213). In addition, it has been suggested that inflammation may also play an important role in DLB, for instance PET imaging and blood biomarkers support an increase in cerebral and peripheral inflammation in the early phases of DLB, while these features appear reduced with disease progression (214, 215). Numerous studies have reported a greater male predominance in the incidence, prevalence, and mortality, although these findings are inconsistent (216–221) (please see Table 5).

**Table 5:** Sex differences in Dementia with Lewy Bodies (DLB) studies from 2012 to 2022.

Author/year country type of study	Subtype	Sample size	Methods	Main findings	Critical evaluation
Disease diagnosis: epidemiology, prevalence, demographics, survival rate					
Mouton et al. (216) French National Alzheimer Database A repeated, cross-sectional study	DLB AD PD PDD	DLB: *N* = 10,309 (80.11 ± 7.84) *M* = 4,674\u00B0F = 5,635 AD: *N* = 135,664 (81.42 ± 7.98) *M* = 40,566\u00B0F = 95,098 PDD: *N* = 3,198 (79.45 ± 8.09) *M* = 1746\u00B0F = 1,452 PD: *N* = 8,744 (73.86 ± 10.79) *M* = 4,979\u00B0F = 3,765	Demographics and Clinical Assessments: Variables such as gender, age, living conditions, education level, type of center, and location of patients were collected Cognition: MMSE Sex ratio and demographic data were compared using multinomial logistic regression and a Bayesian statistical model	Sex ratios (female percent/male percent) were different across the four groups; DLB: 1.21 (54.7%/45.3%); AD: 2.34 (70.1%/29.9%); PD: 0.76 (43.1%/56.9%) and PDD: 0.83 (45.4%/54.6%) There were significant differences between each group (including DLB), but not between PDD and PD, which had a similar sex ratio	Large sample size Diagnoses were made by clinical judgment and not according to anatomopathological results Data entry by different physicians
Gan et al. (217) Beijing, Tianjin, China Retrospective, clinical study	DLB PDD	DLB & PDD: *N* = 455 *M* = 239 (age onset = 69.2 ± 8.1) *F* = 216 (age onset = 68 ± 8.8)	Clinical Assessments: Cognitive fluctuations: The Mayo Fluctuations Composite Scale Visual hallucinations: NPI Delusions and depression from Parkinsonism: UPDRS III RBD: RBDSQ/ Video-PSG MRI/PET/DAT	There were slightly more males than females with DLB (50.9%) and PDD (57.9%) Patients with DLB had a poorer performance compared to those with PDD on the MMSE (*p* = 0.001), the MoCA (*p* < 0.001), the CDR (*p* = 0.002) and the MTA (p = 0.002).	Retrospective study design which could introduce recall bias Diagnoses of the patients were not subsequently validated by autopsy, which is the gold standard for a diagnosis Not all patients were diagnosed using the updated protocols – inconsistencies Gender differences were only focused on the prevalence not in other domains within PDD and DLB
Savica et al. (218) Minnesota, USA Epidemiologic study	DLB PDD	DLB: *N* = 64 PDD: *N* = 46	Diagnostic criteria included two steps: the definition of parkinsonism as a syndrome and the definition of the different types of parkinsonism within the syndrome Reliability and validity of diagnosis checks	The incidence rate of DLB was 3.5 per 100,000 per person-years overall, and it increased steeply with age Patients with DLB were younger at onset of symptoms than patients with PDD and had more hallucinations and cognitive fluctuations Males had a higher incidence of DLB than females across the age spectrum. The pathology was consistent with the clinical diagnosis in 24 of 31 patients who underwent autopsy (77.4%)	It is possible that some patients with mild symptoms might go unrecognized and hence undiagnosed Some of the clinical features (e.g., cognitive fluctuations) were not systematically recorded in medical records Cognitive status was not systematically studied in all patients with parkinsonism
Price et al. (219) Cambridge, United Kingdom Retrospective study	DLB AD	DLB: *N* = 251 (age at diagnosis = 79.3 ± 7.6) *M* = 122\u00B0*F* = 129 AD: *N* = 222 (age at diagnosis = 80.2 ± 8.8) *M* = 83\u00B0F = 139	Case identification: Searches of diagnosed DLB on electronic records across an 8-year period Demographics, clinical and temporal data extracted Other information: Medications, mortality	Median survival was 3.72 years for DLB and 6.95 years for AD Controlling for age at diagnosis, comorbidity and antipsychotic prescribing, the model predicted median survival for DLB was 3.3 years for males and 4.0 years for females	The retrospective nature of the study meant that accurate estimation of the timing of symptom onset was not possible, limiting the ability to report the duration of illness accurately The findings of this study do not reflect the total populations with these diagnoses–diagnosis in a secondary care setting may reflect greater symptom
Boot et al. (220) Rochester, USA Retrospective study	DLB AD	DLB: *N* = 147 (age at diagnosis = 72.5 ± 7.3) *M* = 113\u00B0F = 34 AD: *N* = 236 (age at diagnosis = 74.9 ± 10.1) *M* = 90\u00B0F = 146 Controls: *N* = 294 *M* = 226\u00B0F = 68	Demographics and clinical history 19 Candidate risk factors (i.e., family history, depression, diabetes)	Compared to controls, DLB patients were significantly more likely to have a history of anxiety, depression, a family history of PD, and carry APOE4 alleles but less likely to have had cancer Compared with AD patients, DLB patients were significantly younger and more likely to be male, have a history of depression, be more educated, and have a positive family history of PD.	Relatively small sample size Some reports of missing data
Abdelnour et al. (234) European DLB (E-DLB) Consortium A multicentre, international study	DLB	*N* = 107 (68 ± 8.7) *M* = 77\u00B0F = 30	Clinical, neuroimaging and CSF assessments: Assessments for parkinsonism, visual hallucinations, RBD and other clinical core features Atrophy: MRI Amyloid-b and tau neurofibrillary tangles were assessed through CSF levels of AB42 and phosphorylated tau (p-tau) using enzyme-linked immunosorbent assays (ELISAs)	Hierarchical clustering identified 4 clusters: (1) Cluster 1 was characterized by amyloid-b and cerebrovascular pathologies, medial temporal atrophy, and cognitive fluctuations; (2) Cluster 2 had posterior atrophy and showed lowest frequency of visual hallucinations and cognitive fluctuations and the worst cognitive performance; (3) Cluster 3 had the highest frequency of tau pathology, showed posterior atrophy, and had a lower frequency of parkinsonism; (4) Cluster 4 displayed normal AD biomarkers, the least region brain atrophy and cerebrovascular pathology, and the highest MMSE scores Cluster 4 showed a slight predominance of females, while the whole cohort was mostly constituted by males	Relatively small sample size
Jones and O’Brian (221) Newcastle, Cambridge, United Kingdom Systematic review	DLB	Total of 31 studies included in this review	Literature review of all relevant population and clinical studies conducted using PubMed	Only eight prevalence studies included the sex of those with DLB Five of these studies reported disproportionately more females with the disease when controlling for the sex of DLB population (271–275) The three remaining studies reported disproportionately more males (276–278).	Need more representative samples There is a need to increase the likelihood of accurate diagnosis on a case-to-case basis.
Genetics					
Gámez-Valero et al. (228) Barcelona, Spain Post-mortem, clinical cohort study	DLB	Post-mortem: DLB = 50 PD = 43 Controls = 34 Clinical cohort: DLB = 47 (75.8) Controls = 131 (72.3)	Post-mortem brain samples with clinical and neuropathological diagnoses were obtained from tissue bank GBA Mutation Screening: 11 DNA fragmentations and sequencing	16 GBA mutation carriers were identified, 5 of which were brains with pure DLB The most common mutation, E326K, was strongly associated with pure DLB and PD with dementia 3. GBA mutations were overrepresented in males and associated with earlier DLB onset	There is lack of consideration of other factors such as clinical characteristics and lifestyle factors
Liu et al. (229) Jilin, China Meta-analysis	DLB	Total of 14 studies included in this review	PubMed, Cochrane and EMBASE databases were used to retrieve related studies The odds ratios and 95% confidence interval were calculated to determine the association between GBA and DLB and between GBA and the clinical characteristics of DLB	This meta-analysis confirmed that the GBA variant rate was significantly higher in DLB group than in the control group, as were the variant rates of L444P, N370S, and E326K, whereas the variant rate of T369M showed no significant difference between the groups. The GBA variant group had a younger age of onset and lower MoCA score than the GBA non-variant group in DLB patients There were no significant sex differences in GBA variants between sexes	Lack of consideration of other factors that might affect occurrence and severity of DLB such as education level, smoking history and living habits
Clinical Features: Non-motor Symptoms					
Utsumi et al. (13) Hokkaido, Japan Retrospective, clinical study	Probable DLB	*N* = 234 (age at diagnosis = 79 ± 7.5) *M* = 101 (age at diagnosis = 78.6 ± 6.7) *F* = 133 (age at diagnosis = 79.2 ± 8)	Initial symptoms assessment by an interview with patients and caregivers in nine initial symptoms: Cognitive impairment Visual hallucinations Parkinsonism RBD (e.g., frequent shouting) Depression Auditory hallucinations Delusions Disturbance of consciousness Syncope DLB-related symptoms at diagnosis, all the above (except cognitive impairment) and four symptoms: Fluctuations in attention and arousal levels Orthostatic hypotension Constipation Hyposmia	Initial symptoms findings: A larger proportion of females than males initially present with psychiatric symptoms. For all assessed psychotic symptoms, females had higher rates than males, and there was a significantly higher rate of auditory hallucinations in females than in males RBD was significantly more frequent in male than female patients DLB-related symptoms at diagnosis: There were significantly higher rates in males than females in the incidence of RBD There was also a significant difference between males and females in RBD, parkinsonism, hyposmia and syncope (higher rates in males) at diagnosis Females experienced significantly more auditory hallucinations than males	No PSG was used to confirm RBD
Chiu et al. (231) Taiwan, China Cross-sectional, longitudinal clinical study	DLB	*N* = 152 *M* = 87\u00B0F = 65	Demographics and Clinical Assessments: Patients were interviewed by a trained neuropsychologist for the assessment of the NPI domain of hallucinations that included ratings on eight individual forms of hallucinations CDR Cognitive function: MMSE, CASI UPDRS Cumulative frequency, 1-month frequency and phenomenology of VHs were summarized and compared between females and males with DLB.	Females had a higher frequency of visual hallucinations of nonfamily people, passed families and nonchildren families. After adjusting for age and dementia severity, factors associated with VHs among all patients with DLB were female gender, longer duration of psychiatric disorder, higher total NPI score, a higher caregiver burden score and higher rates of antipsychotics	Comparison of the factors associated with VHs DLB in this study is cross-sectional. Hence, we cannot speculate on the causal relationship of factors with dementia Diagnostic criteria: lack of dopamine transporter uptake imaging until 2010, the revised consensus criteria were not available in the hospital for the first two years; therefore, a lower diagnostic rate for probable DLB may be observed
Tsunoda et al. (230) Kumamoto, Japan Cross-sectional, retrospective study	DLB	*N* = 124 (78.3 ± 5.6) *M* = 54\u00B0F = 70	Screening/Assessment: Routine laboratory testing: Vitamin B1, Vitamin B12, thyroid function Cognitive function: MMSE NPI MRI/Computed tomography and single-photon emission computed tomography for cerebral perfusion Neuropsychiatric Symptoms: Hearing impairment Semi-quantitative interview with primary caregivers using NPI: Auditory hallucinations, visual hallucinations	35.5% of patients had AHs, and 60.5% had VHs 90.9% with AHs also had VHs 90% of patients hear the AHs in the form of a soundtrack of the scene The presence of AHs was significantly more likely to be associated with female patients and those with hearing impairments	Internal psychiatric symptoms such as AHs cannot be directly studied because of patients’ incomplete recollection Selection bias because of clinical diagnostic criteria for DLB – makes the prevalence of DLB patients with pure AHs lower than it is Multiple comparison problem: Type I error
Bayram et al. (233) Data obtained from the NACC Neuropathology Data Set, Genetic Data, and Uniform Data Set (UDS) Case-controlled retrospective study	Pathological confirmed DLB	*N* = 211 *M* = 156 (age at last visit = 75.9 ± 8.4) *F* = 55 (age at last visit = 80 ± 8.7)	Before death: CDR-SOB NPI-Q UPDRS-III Clinician report of DLB core features (i.e., cognitive fluctuations, VHs) at any visit during data collection Autopsy: LB pathology staging Thal phase (amyloid-B plaque score) Braak tau stage (neurofibrillary tangle stage) CERAD (neuritic plaque score) Level of substantia nigra	Females were more likely to die older, have fewer years of education, and had a higher tau burden Females were also less likely with dementia and clinical DLB Females reported lesser VHs than males	This study used a relatively small sample size of participants with limbic or neocortical stage LB pathology without cognitive impairment No consideration of medications being taken for motor, behavioral, and cognitive symptoms Pathological assessments recorded did not focus on regional severity – need finer grain comparisons
Symptomology: non-motor symptoms; sleep					
Choudhury et al. (232) Minnesota, USA Longitudinal clinical study at the Mayo Clinic Alzheimer’s Disease Research Center (ADRC)	DLB	*N* = 488 (age at first visit = 73) *M* = 370 (age at first visit = 72) *F* = 118 (age at first visit = 75)	Clinical assessments: The clinician obtained information regarding each core feature’s presence or absence Recurrent episodes of dream enactment behavior during sleep with movements that appeared to match dream content Parkinsonism neurological examination: Parkinsonism severity: UPDRS 4-item Mayo Fluctuation Scale for cognition GLDS Cognition: MMSE and DRS Neuropathological examination	RBD is more apparent at a younger age in males than in females Males were more likely to develop RBD before the onset of cognitive symptoms, while females were more likely to develop RBD and cognitive symptoms within the same time frame Females met clinical criteria for probable DLB at an older age and after a longer latency from cognitive onset Only half of the females in this study reported a history of RBD, compared to 84% of the males At initial visit, females were older and more cognitively impaired than males Females were also more likely to have visual hallucinations than males. In males, the clinical cohort and autopsy subset showed that visual hallucinations were more likely to emerge after the other core features in men, while females did not demonstrate this time lag	This study was carried out in a tertiary care setting with referral patterns that may limit generalizability to other settings This study did not include biomarkers, clinical symptoms
Mechanisms: inflammatory responses, brain structures etc.					
Van de Beek et al. (236) Amsterdam, Netherlands; Amsterdam Dementia Cohort Retrospective, clinical study	DLB	*N* = 223 *M* = 184 (67.7 ± 7.3) *F* = 39 (70.1 ± 6)	Clinical and cognitive features: Hallucinations: NPI Neurological examination: i.e., tremor/bradykinesia and/or rigidity Semi-structured patient history interview RBD Depression: GDS MMSE Memory: Verbal learning test (RAVLT) Attention and speed: TMT-A, TMT-B Apolipoprotein E genotyping QIAamp DNA blood isolation kit CSF Analysis	Females had lower CSF alpha-synuclein and CSF AB42 levels compared with male Females were significantly older, had a shorter duration of complaints, more frequent hallucinations and scored lower on MMSE and fluency task No significant differences were found for fluctuations, RBD, parkinsonism, other cognitive tests, or tau concentrations	Well-defined, large sample of DLB patients with a clinical diagnosis of DLB supported by DAT Retrospective design – not all features reported for all patients CSF total alpha-synuclein is not yet validated as a clinically useful marker in DLB – there may be differences in sensitivity between different alpha-synuclein species A small number of patients had normal DAT imaging, which is not supportive of DLB diagnosis, but clinical diagnosis made in tertiary centers
Ferreira et al. (227) Multicentre cohort (Combination of E-DLB and the Mayo Clinic DLB Cohort) Prospective study	DLB	*N* = 417 *M* = 287 (70.2 ± 8.6) F = 129 (72.5 ± 8.2)	Demographics and clinical assessments: Medical history review, informant interview, neurological examination, and neuropsychological assessment (i.e., MMSE) B-amyloid and tau biomarkers: B-Amyloids (A+) and tau NFT (T+) were measured with CSF biomarkers and PET imaging Patients were stratified into 4 groups: A-T-, A + T-, A-T+, and A + T+	The percentage of A-T-decreased with age, and A+ and T+ increased with age in both males and females A+ increased more in APOE e4 carriers with age than in noncarriers A+ was the main predictor of lower cognitive performance when considered together with T+ T+ was associated with a lower frequency of parkinsonism and probable RBD A + T+ was more common in females than males compared with the A − T− and A − T+ groups.	Multicentre study added the value of increased statistical power and ability to generalize the findings
Bayram et al. (226) NACC Uniform Data Set (UDS) Retrospective study	DLB	*N* = 691 *M* = 468 (Age at last visit = 76.4 ± 8.9) *F* = 223 (Age at last visit = 79.9 ± 10)	Clinical and neuropathological assessments: Males and females were divided into two groups based on the staging of LB and AD pathologies CDR-Dementia Staging Instrument-Sum of Boxes Thal phase (amyloid-B plaque score), Braak tau stage (neurofibrillary tangle stage) and Consortium to Establish a Registry for Alzheimer’s Disease (CERAD) score (neuritic plaque score)	Females with more severe AD copathology and tau had worse cognitive decline and higher likelihood of AD clinical phenotype than males Males with more severe AD copathology had lower likelihood of LB clinical phenotype than females Interaction of sex and pathology was more prominent in those aged between 70 and 80 years	Analyzes included only clinician reports of LB core clinical features, because of significant amounts of missing data for other features that may help with clinical identification of LB disease Clinical diagnosis and cognitive status of NACC were determined by a single clinician, a group of clinicians or an *ad hoc* consensus group which may include a combination of detailed examination
Sarro et al. (237) NACC; Rochester, USA Retrospective study	DLB AD Dementia	DLB: *N* = 81 (Age at MRI = 72 ± 8) *M* = 67\u00B0F = 14 AD Dementia: *N* = 240 (Age at MRI = 75 ± 10) *M* = 135\u00B0F = 105	Clinical and neuropathological assessments: MMSE, DRS, CDR-Sum of Boxes, UPDRS-III, Mayo Fluctuations Questionnaire Neuropathology assessment: Consortium to Establish a Registry for Alzheimer’s Disease (CERAD) MRI	DLB patients had a higher white matter hyperintensities (WMHs) volume compared to controls, and WMH volume was higher in the occipital and posterior periventricular regions in DLB compared to AD Female sex and older age were associated with higher WMH volumes in both DLB and AD dementia groups	Relatively smaller sample size There is lack of consideration of other clinical characteristics and lifestyle factors
Wennström et al. (238) Malmö, Sweden Retrospective study	DLB AD	DLB: *N* = 18 (74 ± 7) AD: *N* = 26 (73 ± 6) Non-demented controls: *N* = 24 (72 ± 8)	Clinical assessments and CSF profile: Demographics and neuropsychological assessments (i.e., MMSE) a1-antichymotrypsin (ACT) concentrations in CSF and the basic CSF AD-biomarker profile (AB1-42, T-Tau, P-Tau181) CSF Orexin samples were determined using radioimmunoassay CSF alphasynuclein was determined using enzyme-linked immunosorbent assay (ELISA)	There was a decrease in CSF orexin concentrations in DLB as compared to AD patients and controls. The observed differences in orexin levels were found to be specific to females with DLB patients Females with DLB also exclusively displayed lower levels of alphasynuclein compared to AD patients and controls Orexin was associated to alphasynuclein and total Tau in female non-demented controls whereas associations between orexin and AB1-42 concentrations were absent in all groups regardless of gender	Very small sample size
Interventions: pharmacological					
Agbomi et al. (235) South Carolina, USA: PRISMA Health Registry Retrospective study	DLB PDD	DLB: *N* = 608 *M* = 332 (75.93 ± 9.18) *F* = 276 (81.74 ± 9.24) PDD: *N* = 7,594	From PRISMA Health registry: Cognition: MMSE, MoCA, Saint Louise University Mental Status Examination History of alcohol, tobacco, and length of stay in the hospital Medication use: ChEIs, SGAs, or SSRIs	ChEIs, including donepezil, galantamine, and rivastigmine, were associated with DLB SGAs such as risperidone were associated with females with DLB Olanzapine, escitalopram, and tobacco use were associated with males with DLB	Data entry by different physicians – no external validation that standard criteria were met No differentiation was made for patients with early and late DLB PRISMA patients are not fully representative of the total DLB/PDD population No outcomes of tests mentioned, i.e., MMSE Retrospective study

AD, Alzheimer’s Disease; CASI, Cognitive Abilities Screening Instrument; ChEIs, Central Acetylcholinesterase inhibitors; CDR, Clinical Dementia Rating; CSF, Cerebrospinal Fluid; DAT, Dopamine Active Transporter; DLB, Dementia with Lewy Bodies; DRS, Mattis Dementia Rating Scale; F, Females; GDS, Geriatric Depression Scale; GLDS, Global Deterioration Scale; LB, Lewy Body; M, Males; MMSE, Mini Mental State Examination; MoCA, Montreal Cognitive Assessment; MRI, Magnetic Reasonance Imaging; N, Total Sample Size; NPI, Neuropsychiatric Inventory; PET, Positron Emission Tomography; PD, Parkinson’s Disease; PDD, Parkinson’s Disease Dementia; PSG, Polysomnography; RBD, Rapid Eye Movement Behavior Disorder; RBDSQ, REM Behavior Sleep Disorder questionnaire; SGAs, Second Generation Antipsychotics; SSRIs, Selective Serotonin Reuptake Inhibitors; TMT, Trail Making Test; UPDRS, Unified Parkinson’s Disease Rating Scale; VHs, Visual Hallucinations.

In a retrospective study on Parkinson’s Disease Dementia (PDD) and DLB in China, DLB was found to be more common in women in the age group 60 to 69 years but more balanced in younger age groups (217). In contrast, for age groups older than 70 years, males have a greater prevalence of DLB than females (217). Further severity-stratified analyzes revealed that males were more likely to visit their physician when experiencing mild symptoms in both PDD (63.6%) and DLB (56.9%), while females were more likely to visit only when experiencing moderate to severe symptoms levels (217), reiterating the need for more focus on early stages of DLB in females.

Other studies on sex distribution in DLB show inconsistent findings of DLB incidence between sexes (216, 221). In a cross-sectional study of DLB, AD, PD, and PDD, Mouton et al. reported a slight predominance of females with DLB, particularly in those older than 75 years and the sex ratio with a preference for females increased with age (216). These inconsistencies in sex distribution findings of DLB could be due to three reasons. Firstly, most DLB diagnoses were made by clinical judgments rather than pathological results. Nelson et al. posited that clinically suspected DLB was more likely to be over-diagnosed in females, which might explain this variation in the prevalence of DLB in different studies (222). Secondly, DLB shares similarities in pathological and clinical characteristics with AD, which may result in a higher proportion of females being diagnosed as AD is predominantly associated with female sex (223–225). For instance, a recent study reported that females with DLB had a higher Braak tau staging and less nigrostriatal loss than males with DLB, despite having similar Lewy body staging with males with DLB (226). Thirdly, there is also a genetic component to DLB (227, 228). For example, a clinical cohort study reported the association of GBA mutations with early onset DLB and male sex, although these findings have been somewhat inconsistent (228, 229).

Sex differences have also been reported in the initial symptoms of DLB diagnosis (13). In the initial stage of clinical manifestations, females with DLB exhibited a significantly higher overall rate of psychiatric symptoms (*p* = 0.009), particularly in auditory hallucinations (AHs) (*p* = 0.012), while males with DLB had a higher incidence of RBD (*p* < 0.001) (13). These findings align with Tsunoda et al., in which AHs were significantly associated with female sex (*p* = 0.04) (230).

Visual hallucinations have also been reported in DLB, with different symptomatology profiling between sexes (231–233). Cumulative and 1-month frequency analyzes of visual hallucinations of DLB patients found that the contents of visual hallucinations frequencies of non-family people, passed families, and nonchildren families were significantly higher (231), and earlier in women with DLB than men (232). Additionally, both sexes had distinct predisposing factors associated with visual hallucinations (231). More specifically, older age (*p* = 0.003) and higher neuropsychiatric inventory (NPI) score (p = 0.009) were associated with women with DLB, while severe dementia stage (*p* = 0.008) and higher rates of antipsychotics (*p* < 0.047) were associated with men with DLB (231). Furthermore, in a factorial analysis using the European DLB consortium, Abdelnour et al. parsed DLB clinical presentations into four subtypes and reported a greater predominance of females with DLB with characteristics such as higher MMSE scores, cognitive fluctuations and cerebrovascular pathology (234). This could indicate a distinct phenotype of DLB between sexes and age groups, although this remains elusive (233, 234).

Understanding sex differences also have significant implications in identifying biomarkers, neuropathology and evaluating the efficacy of pharmacological interventions in DLB (226, 227, 235–238). Lower cerebrospinal fluid (CSF) alpha synuclein and CSF amyloid levels were reported in women with DLB, accompanied by distinct sex-specific characteristics, such as more frequent hallucination and lower scores on a cognitive task (236). This aligns with previous study by Wennstrom et al. who reported lower levels of CSF alpha synuclein and CSF orexin concentration, particularly in women with DLB, as compared to AD and controls (238). In other brain biomarkers, females with DLB have also been associated with greater white-matter hyperintensities (WMHs), further reiterating sex-specific biomarker profiles in DLB (237). Finally, in a recent study on medication use history, a differential preference of medications between sexes was reported, with second-generation antipsychotics such as risperidone associated with females with DLB, while olanzapine, escitalopram and tobacco use were associated with males with DLB (235). The pathomechanism behind this diversity is currently unclear.

### 3.4. Multiple system atrophy

Multiple system atrophy (MSA) is an uncommon progressive neurodegenerative disorder characterized by autonomic failure and motor involvement of parkinsonism (MSA-P) or cerebellar ataxia (MSA-C) (11, 239). Autonomic failure in MSA includes orthostatic hypotension, constipation, and sexual and urinary dysfunction (239). In MSA, an astrocytic and microglial activation, along with a significant change in the expression of a subset of inflammation-associated genes, have all been reported in the MSA brain, suggesting that targeting inflammation-related processes might limit the disease progression (240, 241). Sex differences in MSA have been reported in many studies focusing on gender distribution, survival, and clinical features studies (11, 242, 243) (as summarized in Table 6). MSA is known to be more prevalent in men (11, 244), however, other studies focusing on sex-differences report oppositional findings. For instance, some studies quote longer survival in women (11, 242), others report longer survival in men (243, 245, 246) or no differences between sexes (247–253).

**Table 6:** Sex differences in Multiple System Atrophy (MSA) from 2012 to 2022.

Author/year country type of study	Subtype	Sample size	Methods	Main findings	Critical evaluation
Demographics, clinical features, and mortality					
Coon et al. (11) Minnesota, USA Retrospective clinical study	MSA-P MSA-C	*N* = 685 *M* = 356 (age onset = 60.6 ± 9.8 *F* = 329 (61.3 ± 9.3) Alive = 100 *M* = 52\u00B0F = 48	Information of patients’ demographics (i.e., Age onset), clinical features (i.e., ataxia, dream enactment, parkinsonism) and autonomic tests (i.e., Systolic blood pressure) were obtained Living patients were called and examined for symptoms development since last neurologic examination	There are sex and gender differences in MSA in terms of symptoms onset, presentation, and survival: Symptoms: Females were more likely to have a motor onset of symptoms than males. Males were more likely to have autonomic symptoms at onset, which is more severe Age onset: Females were more likely to receive a diagnosis of MSA earlier than males Survival: The difference in time of diagnosis to death is almost one year between males and females, with 3.6 months benefit in females (using a cohort of patients who had died data) Other clinical features: Urinary dysfunction: Less severe urinary dysfunction in females Sexual dysfunction: Rarely addressed in females	The retrospective nature of this study with different providers makes it hard to ascertain patient reporting of symptoms relating to confounding sex-specific factors such as childbirth, menopause etc. Using a large number of patients and standardized questionnaires helped reduce bias and statistical power.
Coon et al. (253) Minnesota, USA Retrospective, clinical study	MSA-P MSA-C	*N* = 685 (60.9 ± 9.6) *M* = 355\u00B0F = 330	Demographics and clinical assessments: Motor and autonomic symptoms were obtained from recorded clinical history, neurological examination, and standardized patient-completed symptom questionnaire Autonomic testing: Autonomic Reflex Screen Survival data were obtained from the clinical record Imaging: MRI scan	Neither MSA subtype, classification as probable or possible MSA, nor sex was significantly associated with survival	Retrospective nature of this study Patients were seen by different providers over a long time, which may account for a difference in the recording of symptoms
Clinical Features: Non-motor Symptoms; Cognition					
Cuoco et al. (258) Salerno, Italy Case-controlled prospective, longitudinal clinical study	MSA-P MSA-C	Start: *N* = 55 *M* = 29 (61.79 ± 8.43) *F* = 26 (62.57 ± 7.51) After one year: *N* = 26/55 Attrition = 29/55 (10 died (4 M, 6F), 19 were unable to return due to worsening of disease (11 M, 8F)	Neuropsychological and neuropsychiatry battery at the start and after one-year follow-up: UMSARS MoCA Memory: Rey auditory verbal learning test (15-RAWLT), the prose memory test, and recall of Rey-Osterrieth figure Attention: TMT-A and Stroop color word test Executive function: Clock design test, SVF, and the copy of Rey-Osterrieth figure Visuospatial: Constructional apraxia test and BJLO Language: two subtests from ENPA, the non-word repetition test and the hearing comprehension test of sentences Functional autonomy: IADL, ADL Mood: BDI-II, AES	At baseline: Females with MSA had lower performance on global cognition abilities and visuospatial abilities Females with MSA exhibited a higher prevalence of depression and apathy than males At follow-up: Females with MSA deteriorated more than males in attention abilities and motor functions and had a higher prevalence of depression than males Mild Cognitive Impairment was more pronounced in females than males Females with MSA deteriorated more than males over time for motor functions and attention	Small sample size The attrition rate at follow-up is high No account of any sex hormones or menstrual cycle in females’ hormones
Clinical Features: Non-motor symptoms; Others					
Yamamoto et al. (12) Chiba, Japan Retrospective, clinical study	MSA-P MSA-C MSA-mixed	*N* = 66 (62.2) *M* = 39\u00B0F = 27	Patients responded to a urinary symptoms questionnaire and underwent urodynamic examination twice: Urinary Symptoms Questionnaire Urodynamic Examination EMG: Performed standard EMG and a motor unit potential (MUP) analysis using an EMG computer, inserted into the most superficial layer of the anal sphincter muscle	There were significant sex differences in reduced urine flow, increased post-void residuals, and decreased contractility at the second examination. At the first examination, night-time urinary frequency and voiding symptoms were significantly more severe in males than in females; however, at the second examination, except for urinary urgency, sex differences were not observed for any other symptoms Urodynamic examination: the degree of detrusor contraction was significantly less in males at the first examination. At the second examination, no significant differences were found in the urodynamic examination	Selection bias: It is known that MSA patients ultimately become bedridden and need urethral catheterization. However, it is not possible to examine such patients The urodynamic examination is the only application to assess MSA patients whose daily living is not severely impaired Not much validation in the urodynamic measure used
Mechanisms: Biomarkers, neurochemical or Inflammatory responses					
Chen et al. (259) Guangzhou, China Cross-sectional clinical study	MSA-P	MSA: *N* = 47 (58.74 ± 10.18) *M* = 31\u00B0F = 16 Controls: *N* = 50	Clinical Assessments: UMSARS Detailed motor examination Global disability scale (IV) H&Y & ADL Webster scale: Assess the degree of motor disability Non-motor symptoms: NMS and PDSS MMSE: Cognitive abilities Blood Sampling - Serum levels of: Hcy UA CRP	Serum Hcy was found to be higher in MSA patients compared to healthy controls, especially in male patients Serum UA was found to be lower in MSA patients when compared to healthy controls, especially in males Levels of Serum Hcy were positively associated with the severity of MSA, such as movement dysfunction, declined cognition, and cardiovascular symptoms	Small sample size Most patients with MSA are at the early stages of the disease – not representative No consideration of sex factors (hormones) or genetic factors
Cao et al. (260) Sichuan, China Clinical, longitudinal study	MSA-C MSA-P	MSA: *N* = 234 *M* = 121\u00B0F = 113 Controls: *N* = 240 Follow-up (longitudinal): *N* = 107 *M* = 56\u00B0F = 51	Clinical information including gender, age, BMI, histories of hypertension and diabetes mellitus (i.e., UMSARS) Fasting serum uric acid concentrations of the MSA patients and controls were measured in the clinical laboratory	Serum acid levels were lower in all MSA patients than that in controls. However, in a gender-specific analysis, this difference was only found in males compared with controls However, the serum uric acid levels were not associated with either increased or decreased occurrence of MSA in females Longitudinal study: The level of uric acid, age, disease duration at initial visit, BMI, gender, and the subtype of MSA did not significantly correlate with the mean rate of annualized changes in the UMSARS	Case controls design – results did not reflect the longitudinal effects of uric acid

ADL, Activities of Daily Living; BDI-II, Beck Depression Inventory-II; BJLO, Benton’s Judgment of Line Orientation; BMI, Body Mass Index; CRP, C-reactive protein; EMG, Electromyography; F, Females; Hcy, Homocysteine; H&Y, Hoehn and Yahr Scale; M, Males; MMSE, Mini Mental State Examination; MoCA, Montreal Cognitive Assessment; MRI, Magnetic Resonance Imaging; MSA, Multiple System Atrophy; N, Total Sample Size; NMS, Non-Motor Scale; PDSS, Parkinson’s Disease Sleep Scale; SVF, Semantic Verbal Fluency Test; TMT, Trail Making Test; UA, Uric Acid; UMSARS, Unified Multiple System Atrophy Rating Scale.

Several differences have been similarly reported regarding clinical presentation at disease onset (11). Women with MSA are more likely to have motor symptoms at onset, while men are more likely to experience severe autonomic symptoms (11). Men with MSA are also more likely to have orthostatic intolerance (*p* = 0.0156) and early catheterization (*p* = 0.0396), which may contribute to worse survival rates (11, 247, 251, 254). The distinct symptoms at onset may prompt women to seek an earlier referral to a neurologist, which could explain an earlier diagnosis of MSA in women (11). Women with MSA were also less likely to experience severe urinary and sexual dysfunction (11, 12, 255). However, there is also a possibility that these autonomic symptoms, such as urinary and sexual dysfunction, are underdiagnosed in women. For example, sexual dysfunction was addressed differently, with a significantly higher number of male patients with documented sexual dysfunction (*p* = 0.0001) than female patients (11). This could be due to a lack of appropriate scales for measuring sexual and urinary dysfunction in women (256, 257).

In line with other subtypes of alpha-synucleinopathies, sex differences in other non-motor symptoms and biomarkers in MSA also displayed a distinct sex-specific phenotype (258, 259). In cognitive abilities of MSA patients, Cuoco et al. demonstrated that at the start of the study, women with MSA had significantly lower performance on global cognitive abilities, language, visuospatial ability, and attention (258). Additionally, at follow-up, women with MSA deteriorated more than men with MSA, particularly in motor functions and their attention abilities, and they had higher prevalence of depression (258). Mirroring this, elevated serum homocysteine levels and lower UA levels have also been reported MSA patients, particularly in males (259, 260). Furthermore, these markers are positively correlated with the severity of MSA, such as movement dysfunction and declined cognition (259). This further corroborates the notion of sex-specific profiling in alpha-synucleinopathies.

## 4. Discussion

We have critically analyzed a body of work to date that investigated sex and gender differences in alpha-synucleinopathies. Our findings simultaneously demonstrate (1) a scarcity of studies that systematically focused on sex and gender differences, and (2) clear phenotypical differences in multiple aspects of alpha-synucleinopathies, solely driven by sex and gender differences. In addition, very little appears to be known about the specific interplay of various sex hormones in humans. Moreover, past clinical studies predominantly focus on the role of estrogen, and its potential protective role against the process of alpha-synucleinopathy, the argument for this is somewhat supported by higher incidence of PD in peri/and menopausal period (168, 169, 261). In preclinical studies, oestradiol and progesterone manipulation in ovariectomised, or gonadectomised mice, has demonstrated distinct sex differences in multiple aspects of alpha-synucleinopathy process (17, 262–267). Importantly, several of these animal models suggest that estrogen deprivation may results in dopaminergic neuron loss and lower dopaminergic binding (268).

Clinical studies on estrogen replacement therapy demonstrate a clear role for the estrogen in improving motor symptoms in post-menopausal women (269, 270). Nevertheless, many pieces of this pathomechanistic puzzle are missing; we are yet to clarify the importance of endogenous versus exogenous estrogen exposure, the causality of estrogen effects on multiple aspects of a disease (i.e., genetics) and the interplay between hormonal changes and the progression of alpha-synucleinopathies. Moreover, the threshold, the time-window (e.g., perimenopause versus postmenopause), and all other potentially modifying factors, to which estrogen confer a neuroprotective effect, remain unknown.

Similarly, there are several methodological caveats that should be considered while evaluating preclinical and clinical studies, all of which are rarely systematically considered in their translational importance. For example, in majority, if not in all, analyzed clinical and preclinical studies, there is a lack of focus on the synergistic and antagonistic effects of different sex hormones on various aspects of alpha-synucleinopathies. Most studies predominantly focus on one specific hormone (i.e., estrogen/progesterone), which makes it impossible to fully understand the pathomechanistic complexity. Additionally, even in clinical studies that included multiple hormone measures, women were frequently excluded (46, 47). Moreover, the stage of their menstrual cycle (e.g., follicular versus luteal stage, or and other endocrinology measures was rarely reported).

In conclusion, there is urgent need for future prospective multi-center studies that will account for a more integrated, representative account of sex differences in alpha-synucleinopathies. We suggest that the ideal research framework should systematically account for (1) a specific subtype and distinct phenotype of alpha-synucleinopathies (2) ethnicity and geographical location, (3) disease progression, rate and severity (i.e., early versus late onset), (4) monitoring menstrual cycle and endocrinology health in women, (5) direct quantification of sex hormones in both sexes, (6) medication history and responses (i.e., hormones replacement therapy) and (7) consideration of societal, cultural and gender factors that could impact treatment of PD.

## Data availability statement

The original contributions presented in the study are included in the article/Supplementary material, further inquiries can be directed to the corresponding author.

## Author contributions

KR selected and reviewed. KR and GD assessed all the eligible studies. All authors contributed to the article and approved the submitted version.

## Funding

This research was funded in whole, or in part, by the Wellcome Trust [103952/Z/14/Z]. For the purpose of open access, the author IR has applied a CC BY public copyright license to any Author Accepted Manuscript version arising from this submission.

## Conflict of interest

The authors declare that the research was conducted in the absence of any commercial or financial relationships that could be construed as a potential conflict of interest.

## Publisher’s note

All claims expressed in this article are solely those of the authors and do not necessarily represent those of their affiliated organizations, or those of the publisher, the editors and the reviewers. Any product that may be evaluated in this article, or claim that may be made by its manufacturer, is not guaranteed or endorsed by the publisher.

## Footnotes

^1^www.ephpp.ca/tools.html
