# Supplementary material for: Sex differences in alpha-synucleinopathies: a systematic review
Source: Front Neurol. 2023 Jul 20;14:1204104. doi: 10.3389/fneur.2023.1204104 (PMC10398394; doi:10.3389/fneur.2023.1204104)
Supplement: Supplementary file 1 [file Table_1.DOCX]

**SUPPLEMENT**

**SS1.** List of studies’ information, aim(s), population group, methods, main findings, and critical evaluation of sex differences in Parkinson’s Disease (PD) from 2012 to 2022.

| **Author/Year**  **Country**  **Type of Study** | **Subtype** | **Sample size (Age at time of study, unless stated otherwise** | **Methods** | **Main findings** | **Critical Evaluation** |
| --- | --- | --- | --- | --- | --- |
| **Demographics/Epidemiology** | | | | | |
| **Brakedal et al. (2022) [43]**  Bergen, Norway  Retrospective study | PD | N = 22,060  M = 12,229 (median age onset = 72)  F = 9831 (median age onset = 73) | 1. Used the Norwegian Prescription database to assess the incidence, prevalence, and mortality of PD in Norway between 2005 and 2017. 2. Predicted PD incidence based on the amount of PD drugs dispensed at least three consecutive times, 30 days apart. 3. The prevalence of PD was defined during the time from incidence to an endpoint of either death or end of observation. | 1. PD prevalence increased over the observation period, with larger changes observed in older age groups – incidence and prevalence of PD increased with age, peaking at 85 years 2. The male/female prevalence ratio was 1.5 across all ages, whereas the incidence ratio increased with age, from 1.4 in those 60 years to 2.03 among those >90 years 3. The mortality among females with PD was equal to or higher than the mortality among males with PD | 1. Older age groups are potentially missed by this study design. 2. Large sample size. 3. There is a possibility of misclassification of diagnosis |
| **de Lau et al. (2014) [45]**  Leiden, Netherlands  Longitudinal Cohort Study | iPD | N = 414 (age onset = 61.14 ± 11.37)  M = 265  F = 149 | **Demographics and Motor assessments:**   1. Information on patients’ demographics (i.e., Age at onset), clinical features (i.e., H&Y stage) and drug prescriptions (i.e., LEDD) were obtained 2. SPES-SCOPA   **Cognitive assessments:**   1. SCOPA-COG 2. BDI 3. SCOPA-AUT, 4. SCOPA-SLEEP 5. SCOPA-PC | 1. Predictors of mortality in Parkinson’s disease include higher age, male sex, cognitive impairment, higher postural instability gait disorder score, and the presence of psychotic symptoms. | 1. This study used a sizable population and a detailed assessment of a broad range of symptoms. 2. The study cohort did not comprise incident PD patients but prevalent cases with varying disease duration. 3. Patients unable to be assessed in hospital were assessed at home reducing selection bias. |
| **Moisan et al., (2016) [48]**  Saint-Maurice, France  Meta-analysis | iPD | N = 149,672 (NA)  M = 74,836  F = 74,836 | 1. PD patients were identified using a prediction model from a database on drug reimbursements. 2. Dose of antiparkinsonian drugs, proportion of time treated in a calendar year, number of GP/neurologist visits and sex were obtained | 1. Age-standardised rates were higher in males (prevalence = 2.865/1000; incidence = 0.490/1000 person-years) than females (prevalence = 1.934/1000; incidence = 0.328/1000 person-years) 2. The overall M-F ratios increased by 0.05 and 0.14, respectively, per 10 years of age 3. Incidence was similar in males and females under 50 years and over 1.6 (p < 0.001) times higher in males than females above 80 years (p < 0.001) 4. A meta-analysis of 22 incidence studies confirmed M-F ratios increased with age (0.26 per decade, p = 0.005). | 1. This study’s approach fails to identify patients that are not registered with the SNIIRAM (e.g., those in institutions with in-house pharmacies). 2. PD cases were not examined directly but instead used a prediction model. 3. Large sample size. |
| **Pinter et al., (2015) [46]**  Innsbruck, Austria  Cohort study | iPD | N = 237 (Age at onset = 62.4 ± 1.25)  M = 126  F = 111 | 1. Information on patients’ demographics (i.e., Age at symptom onset, gender) and clinical features (i.e., presence of rest tremor, asymmetry of parkinsonism or gait disorder at disease onset) were obtained. 2. Deceased patients were identified through multiple sources (including national death registries) and cause and date of death were tabulated using the International Classification of Diseases (ICD) 10 codes, according to 8 general disease categories. | 1. Male sex, gait disorder, absence of classical rest tremor, and absence of asymmetry predicted poor survival in this cohort 2. The standardised mortality ratio (SMR) at 38 years of follow-up was 2.02 (1.76-2.29) | 1. Potential for misreporting the cause of death by the physician. 2. The shorter survival in males in the PD cohort most likely reflects the overall shorter survival of males versus females in the general population. 3. Follow-up study on previous paper done at 20-year point. |
| **Pringsheim et al., (2014) [49]**  Alberta, Canada  Systematic Review and meta-analysis | iPD | Total of 134 studies included in this review | 1. Extracted the following data: Study reference, screening procedure, diagnostic criteria, exclusion criteria, number of PD cases used to estimate prevalence, results, study design, screening personnel and target population. 2. Synthesised data for a meta-analysis using information extracted from papers included in the systematic review. | 1. 47 studies were included in this study. 2. Meta-analysis of worldwide data displayed a rising prevalence of PD with age 3. A significant difference was observed in prevalence by geographical location only for individuals 70 to 79 years old, with a prevalence of 1601 in individuals from North America, Europe, and Australia, compared with 646 in individuals from Asia (p < 0.05) 4. A significant difference in prevalence by sex was observed only for individuals 50 to 59 years old, with prevalence of 41 in females and 134 in males (p < 0.05) | 1. Data from various studies could amount to small differences in diagnostic and inclusion criteria. 2. Data taken from locations around the world increases the geographical robustness of the results. |
| **Savica et al., (2018) [50]**  Minnesota, USA  Cross-sectional study | PD with and without dementia | N = 296  M = 187  F = 109 | 1. A movement disorder specialist established the year of onset and type of parkinsonism from the medical records of patients (i.e., Whether the patient also had dementia). 2. Prevalence and incidence of PD was established, and projections of PD with and without dementia by 2060 was estimated using age – and sex-specific prevalence and US population estimates. | 1. The overall prevalence of dementia increased with age from 0.01% (30-39 years) to 2.83% (≥90 years) | 1. Study do not distinguish between PDD and other alpha-synucleinopathies with dementia symptoms, (e.g., DLB) - only categorises patients as being either ‘with’ or ‘without’ dementia. 2. Variability may be a result of different medical personnel making the diagnosis of PD. |
| **Ulivelli et al., (2022) [44]**  Siena, Italy  Observational descriptive study | iPD | N = 115,004  M = 57,778  F = 57,226 | 1. Calculated standardised mortality ratios (SMRs) and annual mortality rates (AMR), standardised by age and sex for the country of Italy and five main geographical areas within it. | 1. The highest mortality rates were observed in males in North-West and Central Italy between 1980 and 2015. 2. PD mortality decreased from mid-eighties onwards, and then the trend rapidly reverses in the period 1998–2002. | 1. Potential for inappropriate death certification. 2. Mortality statistics rely on the underlying cause of death, although several comorbidities may be contributing factors. 3. International classification of diseases changed from version 8 to 9 during the study. 4. ISTAT coding until 1994 was manual. Therefore, it was susceptible to human error. |
| **Xu et al., (2014) [47]**  Jiangsu, China  Meta-analysis and systematic review | iPD | Total of 8 studies with 72,833 participants included in this review | 1. Original articles (prospective observational studies) were obtained from PubMed and Embase databases 2. Pooled adjusted overall risk ratio (RR) of mortality and 95% confidence interval (CI) were computed either by fixed-effects models or by random-effects models | In pooled analyses, patients with PD had a greater risk of all-cause mortality  Subgroup analyses based on the design, gender, follow-up duration and sample size showed that a consistent positive association between PD and mortality risk in each subgroup | 1. The individual studies included in the meta-analysis had different inclusion criteria in terms of age at enrolment, disease duration, disease severity and methods of PD diagnosis. 2. The duration of follow-up was inconsistent across included studies. |
| **Genetics** | | | | | |
| **Georgiou et al., (2017) [56]**  Cyprus  Case-control study | iPD | **PD:**  N = 230 (66.5 ± 10.5)  M = 126  F = 104  **Controls:**  N = 457 (65 ± 10.7)  M = 228  F = 228 | **Demographics, sampling population:**   1. All Greek Cypriots 2. Questionnaires: demographics, environmental exposures, medical history, lifestyle, and other relevant characteristics such as diet, smoking, alcohol consumption   **Genotyping, determination, and classification of mtDNA haplogroups:**   1. Total genomic DNA was isolated from peripheral-blood lymphocytes or saliva samples using QIAamp DNA Blood mini kit (Qiagen) 2. PCR 3. Haplogroup: web-based prediction tool HaploGrep | 1. PD risk: Significant protective effect of mitochondrial haplogroup cluster N(xR) and supercluster LMN for PD risk only in females 2. Age onset: Females with PD with UKJT and R haplogroup, have a later age of onset of PD 13 and 15 times higher than for PD females with H haplogroup | 1. Small sample size 2. No consideration of disease progression, or duration 3. First investigation of mtDNA haplogroups and PD risk in Cypriot population |
| **Sampaio et al., (2018) [61]**  Recife, Brazil  Retrospective study | iPD | N = 162 (64.0 ± 9.4)  M = 92  F = 70 | **Group classification:**   1. PD patients were classified into two groups based on the medication intake; Group 1: PD treated with a daily levodopa dose equal or below 600mg/day; Group 2: PD treated with levodopa above 600mg   **DNA Extraction and Genotyping:**   1. Genomic DNA extracted from 3mL of peripheral blood of PD patients 2. PCR to genotype MAO-B and COMT variants | 1. Significant differences between PD groups for sex, duration of disease, levodopa therapy duration, age onset and COMT inhibitor 2. MAO-B: MAO-B A and AA genotypes and COMT LL genotype suffered more frequently from levodopa-induced-dyskinesia 3. Males with PD with MAO-B G allele have an increased risk of 2.84-fold to be treated with higher doses of levodopa | 1. Hard to establish causality 2. Relatively small sample size |
| **Cilia et al., (2014) [52]**  Milan, Italy  Cross-sectional study | PD | N = 2392 (65.6 ± 9.9)  M = 1417  F = 975 | **Demographics assessment:**   1. Family history in LRRK2-carriers was evaluated during formal genetic counselling, and questionnaires for non-carriers 2. Clinical features of LRRK2-carriers were compared to those of patients whose molecular analysis was negative for the major LRRK2 mutations and for other mutations in known PD genes 3. UPDRS I-III & H&Y   **Mutation analysis:**   1. R1441C/G/H, G2019S and I2020T analysis with standard methods | 1. LRRK2 mutations were identified in 40 PD patients (1.6%) 2. No major clinical differences were found between LRRK2-carriers and non-carriers 3. Female sex was significantly more common amongst carriers than non-carriers (57% vs. 40%), with no differences in clinical features 4. Family history of PD more common in female, regardless of LRRK2 status | 1. Large sample size 2. LRRK2-carriers were identified within a large consecutive series of patients devoid of bias due to pre-selection based on clinical or demographics features 3. First study to investigate gender effects on a Caucasian non-Jewish population |
| **Gan-Or et al., (2015) [54]**  Montreal, Canada  Meta-analysis, including 17 case-control studies | Sporadic PD | N = 24088 | 1. All case-control studies describing LRRK2 mutations and PD were examined in a sex-stratified meta-analysis | 1. A total of 1080 LRRK2-associated PD patients were identified 2. M:F ratio was 1:02:1.00 (50.6% males and 49.4% females) 3. Among males, LRRK2 mutation carriers had a pooled OR for PD of 4.20 whilst in females, LRRK2 mutation carriers had a pooled OR for PD of 4.73 | 1. Relatively small number of studies 2. Lack of age matching between patient and controls in three of the studies 3. Publication bias |
| **Gatt et al., (2013) [57]**  International Multicentre study  Cross-sectional study | PD | **PD:**  N = 63 (77.02 ± 6.69)  **Controls:**  N = 56 | 1. Tissue and data obtained from a number of UK and Scandinavian clinical cohorts and tissue resources: Clinical and autopsy study 2. Genotyping: TFAM rs2306604 SNP was genotyped 3. qPCR of mtDNA levels | 1. rs2306604 genotype frequencies were significantly different to controls in PDD but not DLB 2. ‘A’ allele was associated with PDD in males only, compared with AG/GG carriers | 1. Small sample size 2. Unclear whether TFAM expression is differentially affected at mRNA or protein level In DLB or PDD |
| **Gusdon et al., (2015) [58]**  Shanghai, China  Cross-sectional study | iPD | **PD:**  N = 484 (60.6 ± 10.1)  M = 266  F = 218  **Controls:**  N = 710 (68.4 ± 10.6)  M = 332  F = 378 | **Genotyping:**   1. DNA was extracted from blood leukocytes, and was performed using restriction fragment length polymorphism, followed by PCR | 1. There was no significant association between 5178A and PD population 2. Subgroup analysis revealed that in males the frequency of 5178A significantly lower in males with PD (aged 60-70) | 1. Relatively large sample size 2. Results not generalizable to other population groups |
| **Liu et al., (2014) [66]**  Wenzou, China  Cross-sectional study | Sporadic PD | **PD:**  N = 341  M = 178  F = 163  **Controls:**  N = 423  M = 226  F = 197 | **Genotyping:**   1. Genomic DNA samples extracted from human peripheral blood using DNA blood mini-kit 2. PCR and restriction fragment length polymorphism used to analyse the alleles of rs12817488 and rs2697962   **Cell isolation and Western blot** | 1. ‘A’ allele of rs12817488 was significantly associated with an aggravated risk of PD. This association was only found in females with PD 2. Protein level of CCDC62 in peripheral blood mononuclear cells has higher expression in PD patients with AA genotype 3. Potential interaction found between two oestrogen-related loci (rs12817488/CCDC62 and rs2697962/PRDM2), particular in female stratum | 1. Testing power is limited |
| **Mariani et al., (2016) [254]**  Rome, Italy  Cross-sectional study | PD | **PD:**  N = 92 (age at onset = 65.2 ± 9.7)  M = 62  F = 30  **Controls:**  N = 112 | **Genotyping:**   1. Genomic DNA from fresh whole blood was prepared 2. Genotyping of singlenucleotide polymorphisms (SNPs) rs1049296, rs701753, rs115552500, rs1799945, rs1800562   **Biochemical investigations:**   1. Serum samples collected after 12-hour fasting 2. Serum concentrations of iron, copper and related proteins analysed | 1. No significant difference in the D544E and R793H variants of the ceruloplasmin gene (CP), the P589S variant of the transferrin gene (TF) and the H63D and C282Y variants of the HFE gene found between PD and controls 2. When effect of sex is considered, an increased probability of having PD is associated with low iron concentration and transferrin-saturation | 1. Small sample size may not be sufficient to detect allelic effects |
| **Zhang et al., (2014) [64]**  Huazhong, China  Cross-sectional study | PD | **PD:**  N = 265  M = 156  F = 109  **Controls:**  N = 269  M = 161  F = 108 | 1. Sequence analysis: Peripheral blood collected from each patient and control. Genomic DNA was extracted. PCR was subsequently conducted. | 1. The A allele of rs7311174 and the T allele of rs2072374 of the NCAPD2 were found to be protective in male with PD patients | 1. Small sample size |
| **Zhao et al., (2015) [62]**  Qingdao, China  Cross-sectional study | Sporadic PD | **PD:**  N = 380 (62.5 ± 10.8)  M = 236  F = 144  **Controls:**  N = 380 (70.4 ± 8.2)  M = 229  F = 151 | 1. Patients subdivided into two groups: early onset PD and late onset PD 2. SNP selection and genotyping: Genomic DNA extraction kit used to extract genomic DNA from peripheral blood samples and genotyped by PCR and restriction fragment length polymorphism | 1. The frequency of rs1927914 C allele of the TLR4 gene was found to be significantly reduced in males with PD compared to male controls | 1. Small sample size |
| **Shu et al., (2018) [53]**  Changsha, China  Meta-analysis | PD | 66 studies | - - - 1. Articles from Medline, Embase and Cochrane databases were extracted, with relevant exclusion/inclusion criteria       2. Publication biases analysis and pooled and sensitivity analysis were conducted | 66 studies of PD patients with G2019S, G2385R, R1628P and R1441G were included  Prominent clinical features of LRRK2-G2019S-related PD were female sex, higher rates of early-onset PD (EOPD) and family history (p < 0.00001, 0.02, < 0.00001) | Due to the lack of sufficient data and small sample size, a meta-analysis of the relationship between other variants such as A419V was unable to be performed  Heterogeneity between studies’ design should be kept in mind  Other factors on lifestyle and social factors should also be considered |
| **Ping et al., (2018) [63]**  Jiangxi, China  Cross-sectional study | iPD | **PD:**  N = 265  **Controls:**  N = 269 | Demographics, clinical history (i.e., early onset versus late onset PD) and medication information were obtained  SNaPshot method was applied to determine gene sequences of patients (i.e., sequence analysis, cell culture and transfection, Western blotting, ROS, and antioxidants measurement, GADPH enzyme activity and apoptosis detection) | The rs1136666 polymorphism of GAPDH was closely associated with PD, particularly in older male patients  The genetic expression of rs1136666 CC could induce cell injury and apoptosis via regulation of the oxidant-antioxidant and apoptosis-antiapoptosis balance | Population sample only consist of Chinese Han population, hence, findings may not be generalizable to other ethnic groups  No standardized definition of what constitutes as early and late onset PD |
| **Cui et al., (2021) [55]**  Shanghai, China  Retrospective, Clinical Study | PD | **LRRK2 G2385R Carriers:**  N = 79  M = 42 (64.7 ± 7.5)  F = 37 (61.3 ± 11.6)  **LRRK2 G2385R Non-carriers:**  N = 534  M = 295 (61.5 ± 10.3)  F = 239 (61.5 ± 10.9) | **Demographics and clinical assessments:**  Demographics such as age, sex and years of education were obtained during a clinical interview  Disease stage: H&Y, ADL, UPDRS-II, III  Quality of life: PDQ-39  Medications: LEDD  Cognition: MoCA  Mood: HAMD, HAMA  Olfactory: SSET  Autonomic function: SCOPA-AUT  Sleep quality: RBD-SQ, ESS  **Genetic Analysis:**   1. Peripheral blood samples were obtained, and DNA was extracted | In male sex, LRRK2 G2385R carriers showed lower risk in cognitive impairment compared with non-carriers (p = 0.003).  In female sex, LRRK2 G2385R carriers showed lower risk in autonomic dysfunction compared to non-carrier (p = 0.04)  In LRRK2 G2385R non-carriers, female sex showed lower risk of impairment in activity (p = 0.007), substantia nigra hyperechogenicity (p = 0.019), autonomic dysfunction (p = 0.016) and higher risk in mood disorders (p = 0.022), compared with males.  In LRRK2 C2385R carriers, female sex showed lower risk of autonomic dysfunction (p = 0.024), compared with males | This sample size for LRRK2 G2385R carriers was relatively small.  Effects of other SNPs and genes cannot be excluded in this study  Some continuous variables are being treated as binary outcomes with arbitrary cut-offs, leading to weakened powerfulness |
| **Biomarkers, neurochemical and neuroinflammatory response** | | | | | |
| **Lee et al., (2018) [67]**  Seoul, Korea  Case-control study | Drug naïve, *de novo* PD | **PD:**  N = 90  M = 41  F = 49  **Controls:**  N = 45  M = 20  F = 25  **PD:**  M with L-UA = 64.8 ± 5.4  M with H-UA = 69.1 ± 10.1  F with L-UA = 68.3 ± 7.3  F with H-UA = 70.5 ± 1.3 | **Clinical assessments:**   1. UPDRS-III 2. BDI 3. MMSE 4. Other demographics measures: total cholesterol, history of smoking, alcohol, hypertension, diabetes, and BMI   **Brain MRI**  **Routine chemistry: Uric acid levels**  **DAT scan** | 1. There was a significant interaction between the lowest (PD-L-UA) and the highest UA level (PD-H-UA) groups according to gender within the dorsal attention network (DAN), executive control network (ECN) and default mode network (DMN) 2. Males with PD-H-UA had higher cortical functional connectivity (FC) than controls 3. Females with PD had lower cortical FC, regardless of UA level within all seeds 4. Male patients with PD-H-UA had increased FC than those with PD-L-UA | 1. Small sample size 2. The differences in the UA levels between patient with PD-H and PD-I were relatively narrow 3. No consideration of other factors that may influence pattern of RSN such as depression or olfaction 4. Cortical seeds used in this study may not sufficiently represent a complete functional connectivity analysis |
| **Baik et al., (2020) [68]**  Seoul, South Korea  Cross-sectional study | *De novo* PD patients | N = 167 (69.3 ± 9.82)  M = 83 (70.19 ± 9.68)  F = 84 (68.41 ± 9.93) | **Demographics and Clinical Assessments:**   - - - 1. UPDRS-III       2. Serum UA levels checked before anti-Parkinson medication administered   **DAT scan (18F-FP-CIT PET)** | 1. There is a significant effect of sex and serum uric acid on the intersubregional ratio (ISR) but not on the asymmetric ratio (AR) 2. The ISR was negatively correlated with serum UA level in all patients with PD (r= -0.156, p = 0.045). This association was more prominent in males with PD (r= -0.422, p < 0.001) 3. There was no significant association between AR and serum UA in any patients 4. Serum UA levels were significantly associated with DAT availability in the posterior putamen on both the more affected side (r = 0.312, p = 0.005) and the less affected side (r = 0.312, p = 0.005) only in males with PD. | 1. Serum UA levels were only checked once at the initial visit, this might not reflect the actual UA levels in vivo 2. No causality can be established between UA levels and DAT patterns 3. Reduced DAT uptake not only reflects the nigral cell death, but compensatory downregulation could also be affected, which may overestimate the presynaptic dopaminergic neuronal dysfunction 4. Region-specific association of UA on DAT availability is not in accordance with a previous single PET study |
| **Seyfried et al., (2018) [255]**  Multiple cities, USA  Cohort study | PD | **PD:**  N = 12 (M = 78.4 ± 3.5; F = 75.8 ± 3.5)  **Controls:**  N = 18 (M = 75.6 ± 2.4; F = 72.6 ± 1.7) | - - - 1. Brain tissues were sourced, and diagnosis were confirmed at autopsy by presence of gross depigmentation of SN and microscopic confirmation of SN cell loss and presence of Lewy bodies in SN  1. Content and composition of the major glycosphingolipids, phospholipids and cholesterols were evaluated by lipid isolation and purification and high-performance thin-layer chromatography | - - - 1. Total SN ganglioside sialic acid content and water content were significantly lower in the males with PD than controls  1. Content of all major gangliosides were reduced in males with PD to such degrees, with greatest reduction in the neuronal-enriched gangliosides, GDIa and GTIb most significantly reduced 2. Distribution of phosphatidylethanolamine, phosphatidycholine, and phosphatidylinositol were also significantly lower in males with PD than controls 3. Distribution of myelin-enriched cerebrosides and sulfatides were significantly higher in males with PD than in controls, suggesting myelin sparing | - - - 1. Very small sample size  1. No distinction of disease progression, severity and manifestations was considered |
| **Bakeberg et al., (2019) [75]**  Multiple cities, Australia  Clinical based study | PD | **PD:**  N = 205 (64 ± 9.38)  M = 128  F = 77  **Controls:**  N = 78  M = 31  F = 47 | **Clinical assessment:**   1. Demographic variables 2. Medication dosage: LEDD 3. Assessments of motor and cognitive function and other disease-related features: UPDRS-III, H&Y, ACE-R   **Serum analysis:**   1. 10ml of blood taken prior to clinical/psychological assessments 2. Serum homocysteine, folate, and vitamin B12 levels | 1. PD patients displayed significantly elevated homocysteine levels (p < 0.001) but not in folate or vitamin B12, as compared to controls 2. A significant positive correlation was found between homocysteine and UPDRS-III score only in males with PD (r= 0.319, p < 0.001) 3. In females with PD, a significant negative correlation between homocysteine levels and total ACE-R score was found (r = -0.449, p < 0.001) 4. Multivariate general linear models further confirmed homocysteine to be a significant predictor of UPDRS-III in males (p = 0.004) and predictive of total ACE-R in females with PD (p = 0.021) | 1. Did not include patients with more advanced PD who were no longer independent 2. No consideration of Homocysteine levels over the course of the disease 3. ACE-R not a comprehensive executive function measure 4. No considerations of other factors that might affect homocysteine levels, such as cerebrovascular disease burden |
| **Wu et al., (2020) [76]**  Zhe Jiang, China  Case-control study | De novo, untreated PD | **PD:**  N = 253  M = 146 (63.8 ± 10.7)  F = 107 (62 ± 9.9)  **Controls:**  N = 218  M = 110 (65.5 ± 11.1)  F = 108 (64.8 ± 11.7) | **Clinical assessments:**   1. BMI, height, weight 2. Cognition: MoCA 3. Anxiety: HAMA 4. Depression: HAMD 5. UPDRS and H&Y 6. Systolic and diastolic blood pressure   **Blood sample collection:**   1. Glycated haemoglobin (HbA1c) 2. Homocysteine (HCY) 3. Uric acid (UA) 4. Total cholesterol (TC) 5. Low density lipoprotein (LDL) 6. High density lipoprotein (HDL) 7. Triglyceride (TG) levels | 1. Females with PD have significantly lower incidence of underweight and higher BMI than males with PD 2. There is a differential pattern of sex differences in serum lipids, HCY levels and depression severity 3. Binary regression analysis demonstrated that only in males with PD patients was underweight associated with UPDRS scores and lower change in SBP and change in DBP (all p < 0.05) 4. BMI was also associated with MoCA and lower UPDRS motor scores in males and lower HAMD scores in females | 1. Relatively small sample size 2. Results are not generalizable to advanced PD or those taking medications |
| **Tremblay et al., (2020) [94]**  Montreal, Canada  Data obtained from PPMI database | *De novo*, unmedicated PD | **PD:**  N = 232  M = 149 (61 ± 9)  F = 83 (60 ± 9)  **Controls:**  N = 117  M = 78  F = 39 | **Clinical and structural MRI:**   1. Brain structural analysis 2. Deformation-based morphometry 3. Cortical thickness 4. Diffusion imaging analysis 5. Network connectivity construction | 1. Males with PD had significantly greater tissue loss than females with PD in 11 cortical regions including bilateral frontal and left insular lobe, right postcentral gyrus, left inferior temporal and cingulate gyrus and left thalamus 2. Females with PD had greater atrophy in 6 cortical regions, including regions in the left frontal lobe, right parietal lobe, left insular gyrus, and right occipital cortex 3. Local efficiency of white matter connectivity showed greater disruption in males with PD in multiple regions such as basal ganglia, hippocampus, amygdala, and thalamus | 1. Study account for the expected effect of age and sex on brain morphometry 2. Males and females with PD included here had similar clinical measures of disease severity and were drug naïve 3. Despite that, there were more males in both controls and patient population 4. Database included data from multiple centres and scanners from around the world - variability/heterogeneity in data |
| **Caranci et al., (2013) [82]**  Pozzilli, Italy  Observational, exploratory cross-sectional study | PD | **PD:**  N = 69 (64.59±9.26)  M = 40 (65.2 ± 9.08)  F = 29 (63.76 ± 9.60)  **Controls:**  N = 110 (64.31 ± 9.17)  M = 57 (64.88 ± 8.4)  F = 53 (63.7 ± 9.99) | **Demographics and clinical assessments**:   1. Medication history, interventions, H&Y, UPDRS   **Laboratory assay:**   1. Alpha-synuclein | 1. Decreased levels of plasma alpha-synuclein were observed in advanced stage males with PD but not females 2. Only in males with PD, plasma alpha-synuclein was associated with cognitive impairments, sleep disorders and hallucinations | 1. No specific scales used for non-motor symptoms quantification 2. No genetic tests were conducted in the 10 familial PD cases used in this study 3. Assessment between alpha-synuclein concentrations and clinical variables are stratified by sex |
| **Gao et al., (2016) [69]**  Three cohorts in USA  Nested case-control study | PD | **PD:**  N = 388  M = 202  F = 186  **Controls:**  N = 1267  Nested = 90 214 | **Demographics and clinical assessments:**   1. Questionnaires: BMI, age, height, weight, chronic disease   **Measurement of plasma urate and potential confounders:**   1. Plasma low-density lipoprotein (LDL) 2. High-density lipoprotein cholesterol 3. Ferritin concentration   **Additional meta-analysis of prospective studies:**   1. Included 325 PD cases from 3 prospective studies | 1. Lower PD risk was associated with higher baseline urate in males (p = 0.018) but not in females (p=0.38) 2. Meta-analysis findings: Also found an association between PD risk and urate only in males with PD (p = 0.03), not in females with PD (p = 0.61) | 1. Large prospective study 2. Large sample size 3. As it is an observational study, cannot exclude possibility of residual confounders 4. All measures of urate level in this study and previous studies included was based on a single measure of urate - no accountability of within-person variability and factors affecting it 5. Generalizability issues as majority of participants were Caucasian with similar social factors |
| **Ho et al., (2014) [83]**  Iksan, South Korea  Cross-sectional study | PD | **PD:**  N = 26  M = 14 (73 ± 2.1)  F = 12 (73 ± 2.6)  **Controls:**  N = 21  M = 10 (70 ± 3.2)  F = 11 (71 ± 3.2) | **Demographics and clinical assessments:**   1. H&Y and UPDRS 2. Medications history   **Urine analyses:**   1. Exosome isolation 2. Western Blot analysis | 1. No significant difference was found in LRRK2, alpha-synuclein, and DJ-1 urine exosome in PD and controls 2. However, when age, disease duration, LEDD, and gender considered as parameters, LRRK2 and DJ-1 protein levels demonstrated gender specific differences 3. DJ-1 was significantly higher by 1.7-fold in males with PD as compared to male controls and increased in an age-dependent manner in males with PD | 1. Small sample size 2. No consideration of other factors that might affect level of urine exosomes |
| **Jesus et al., (2013) [70]**  Seville, Spain  Cross-sectional study | PD | **PD:**  N = 161 (63.2 ± 11.9)  M = 90  F = 71  **Controls:**  N = 178 (60.5 ± 13.6)  M = 108  F = 70 | **Demographics and clinical assessments:**   1. H&Y 2. Medical history 3. Treatment 4. Disease duration and age onset   **Blood sample collection:**   1. Serum UA concentration | 1. PD patients exhibited statistically significant lower serum UA concentration than controls (p < 0.001) 2. Severity of PD is associated with level of UA serum concentration considering both sexes (p < 0.001) or males only 3. No other factors related to UA concentration, except LEDD which was associated with lower serum UA concentration serum only in males (p=0.021) | 1. Relatively small sample size 2. Results not generalizability as PD population is Spanish 3. Causality cannot be established |
| **McFarland et al., (2013) [71]**  Massachusetts, USA  Post-mortem study | PD  AD  DLB  Controls | **PD:**  N = 17 (79.2 ± 7.5)  **DLB:**  N = 13  **AD:**  N = 19  **Controls:**  N = 13 | **Tissue selection:**   1. Post-mortem tissue collection obtained 2. Demographics: disease state, gender, specified brain region, pathology   **Urate and precursor measurements:**   1. Urate and other purine precursors pathway metabolites using HPLC with electrochemical and ultraviolet detection | 1. Urate levels in cortical and striatal tissues was lower in PD and AD, compared to controls, only in males | 1. Small sample size 2. Lack of relevant midbrain tissue 3. Limited corresponding clinical information |
| **Hamid et al., (2019) [81]**  Rome, Italy  Cross-sectional study | iPD | **PD:**  N = 268 (63)  M = 151  F = 117  **Controls:**  N = 319 (52)  M = 142  F = 177 | 1. Thorough exclusion/inclusion criteria screening: i.e., MMSE score > 26, presence of dementia etc 2. Human blood sample collection 3. NAPE quantifications | 1. Results demonstrated panel of NAPEs may be able to be a biomarker for PD female patients from healthy controls, supporting the observed downregulation of these NAPEs in plasma of a mouse model of PD (6-OHDA) (part of study) | 1. What does observed biomarker means in terms of behaviour remains elusive |
| **Zhang et al., (2012) [72]**  Hunan, China  Cross-sectional study | PD | **PD:**  N = 534 (63.87± 11.84)  M = 341  F = 193  **Controls:**  N = 614 (63.56 ± 11.8)  M = 384  F = 230 | **Blood sample collection:**   1. Serum UA concentration 2. Haemoglobin 3. Glutamate-pyruvate transaminase (GPT) 4. Glutamic oxaloacetic transaminase (GOT) 5. Urea nitrogen (UN) 6. Creatinine (Cr)   **Demographics and clinical assessments:**   1. H&Y 2. BMI, weight, height | 1. Level of serum UA levels was significantly lower in PD patients than controls and were correlated with PD progression and duration in Chinese population 2. Only hyperuricemia is more strongly associated with lower rates of PD in males, compared to females and older people compared to younger people | 1. No consideration of factors that could affect serum UA concentration 2. Results not generalizable as it is only for Chinese population |
| **Shen et al., (2013) [73]**  Hangzhou, China  Meta-analysis study | PD | N = 33 185  M = 20 641  F = 12 544 | 1. NCBI (PubMed), Web of Science and EMBASE was used to identify studies that reported the risk of PD associated with serum urate 2. Fixed or random effects meta-analysis was used to pool results across studies | 1. 6 studies were identified 2. A 33% reduction in PD incidence among individuals with high serum urate level 3. Subgroup analysis conducted found a statistically significant protective effects of serum urate in males but not in females 4. High serum urate levels seem to slow the clinical decline of PD patients | 1. Results based on observational studies, cannot determined causality 2. All studies attempted to control for other confounders but differently 3. Publication bias |
| **Huang et al., (2021) [84]**  New Taipei, Taiwan  Cross-sectional study | iPD | **PD:**  N = 259 (62.5 ± 9.1)  M = 137  F = 122  **Controls:**  N = 110 | **Clinical and demographics assessments:**   1. Age, sex, educational, marriage, alcohol, and smoking behaviour 2. H&Y and UPDRS-III 3. Depression: HAMD-17   **BDNF Examination:**   1. Peripheral blood collection | 1. The serum BDNF levels were lower in depressed PD patients as compared to non-depressed and controls (p < 0.001) and negatively correlated with UPDRS III score (p < 0.001) and H&Y stage (p < 0.001) 2. Decreased BDNF levels were associated with females only among depressed PD patients (r = -0.45, p<0.001) 3. HAMD-17 score was negatively correlated with BDNF levels (r = -0.59, p < 0.001) and positively associated with UPDRS III score (r = 0.51, p < 0.001) | 1. Cross-sectional design limits causal inference between clinical features and BDNF 2. Depression diagnosis was only based on HAMD-17 rather than DSM-IV 3. No consideration of other factors such as family history or employment data 4. Serum BDNF level taken only at a single point |
| **Zhang et al., (2022) [77]**  Beijing, China  Cross-sectional study | iPD | N = 279 (64)  M = 159 (64)  F = 120 (64.5) | **Demographics and clinical assessment:**   1. Age at admission, sex, weight, height, disease duration 2. Medication: i.e., lipid lowering medicine, LEDD 3. H&Y and UPDRS-III 4. MoCA 5. HAMA and HAMD   **Laboratory assessments:**   1. Total cholesterol (TC) 2. LDH-C 3. HDL-C 4. TG 5. LDL-C 6. HDL-C 7. Apo-A1 8. Apo-B | 1. Females with PD showed significantly higher serum TC, LDL-C, HDL-C, Apo-A1, Apo-B levels than males with PD 2. After accounting for covariates, lower serum TG levels were significantly associated with higher UPDRS-III total scores and gait/postural instability sub scores 3. In males with PD, serum HDL-C or Apo-A1 levels were significantly associated with tremor sub scores | 1. Cross sectional nature so effect over time was not assessed 2. Genetic confounding not considered 3. No considerations of other vascular risk factors such as diet, physical exercise, smoking |
| **Meoni et al., (2022) [80]**  Kassel, German  Cross-sectional cohort study | *De novo* PD | **PD + Controls:**  N = 329  **Training Cohort:**  N (PD) = 72  N (Controls) = 59  **Independent validation cohort:**  N (PD) = 178  N (Controls) = 20 | - - - 1. Clinical assessments (i.e., MMSE, PSG for RBD), demographics and clinical history were obtained       2. NMR sample preparation and analysis of training and independent validation cohort       3. Spectral processing       4. Serum and lipoprotein identification and quantification: 27 metabolites and 111 lipoprotein components identified and quantified. | 1. There was a more pronounced fingerprint of metabolite and lipoprotein profiles pathology in males with PD patients, more particularly altered levels of acetone and cholesterol in males with PD | Training and independent validation cohort were useful in detecting the different metabolites profiles  No consideration of disease severity, symptoms manifestation and disease progression  No consideration of other lifestyle factors that might affect lipids and metabolites levels |
| **Luca et al., (2022) [78]**  Sicily, Italy  Cross-sectional study  (Part of PACOS study, The PArkinson’s disease COgnitive impairmentStudy) | Non-demented PD | N = 348 (age onset = 62.6 ± 10.5)  M = 200 (age onset = 63.1 ± 10.6)  F = 148 (age onset = 61.9 ± 10.4) | **Clinical and neuropsychological evaluation:**   1. Medical records 2. UPDRS-III 3. LEDD 4. FAB   **Serum analyses: Fasting blood samples**   1. Hypercholesterolemia 2. Hypertriglyceridemia 3. Low HDL cholesterol 4. High LDL cholesterol | 1. Females exhibited significantly with higher TC, LDL, and HDL than males 2. Positive association between hypertriglyceridemia and FAB score was found in females (p = 0.013). 3. There was a statistically significant negative association between hypercholesterolemia and FAB score and between high LDL levels and FAB score in males (p = 0.018). | 1. Relatively large sample size 2. Executive functioning measures were constraint to simply a frontal battery task – no investigation into different cognitive domains were done 3. Lack of consideration of disease duration and progression variability 4. No causality between executive functioning and lipid fractions can be established 5. No healthy controls group |
| **Bakeberg et al., (2021) [79]**  Data from Australian Parkinson’s disease Registry  Cross-sectional study | PD | N = 182 (64.5 ± 9.5)  M = 114 (64.9 ± 10.4)  F = 68 (63.9 ± 7.8) | **Clinical and demographics assessments:**   1. LEDD 2. UPDRS-III 3. SCOPA-Cog 4. ACE-R   **Serum analysis:**   1. Serum TC 2. LDL 3. HDL 4. Triglyceride (TRG) levels | 1. Females with PD showed significantly higher lipid subfraction levels (TC, HDL, LDL) as compared to males with PD 2. After accounting for covariates, HDL levels were highly associated with poorer cognitive performance across multiple domains only in females with PD | 1. Relatively small sample size 2. Causality cannot be established 3. Patients assessed in different centres - scoring variability 4. No accountability of other vascular factors that might influence serum levels |
| **Baldini et al., (2020) [85]**  Luxembourg  Clinical study | PD | **PD:**  N = 147 (69.3 ± 8.6)  M = 101  F = 46  **Controls:**  N = 162 (63.3 ± 8.3) | - - - 1. Detailed clinical assessment including neurological examinations and neuropsychological tests and self-reported questionnaires       2. Stool samples were obtained and analysed by 16S rRNA gene sequencing       3. Prediction for potential secretion for 129 microbial metabolites were done through personalised metabolic modelling using the microbiome data and genome-scale metabolic reconstructions of human gut microbes | 8 genera and 7 species changed significantly in their relative abundance between PD and controls  PD-associated microbial patterns were statistically depended on sex, age, BMI, and constipation. For instance, *Paraprevotella* was significantly reduced in females with PD in comparison with males but not in female controls (p < 0.05). Relative abundances of *Bilophila* and *Paraprevotella* were also significantly associated with H&Y staging after controlling for disease duration  Predicted microbial pantothenic acid production potential was associated to the presence of specific non-motor symptoms | Other factors such as diet, exercise and smoking were not included  Small sample size to detect any effects of drugs  16S rRNA gene sequencing does not allow analyses on the strain level as it could lead to misclassification |
| **Houser et al. (2018) [88]**  Georgia, USA  Cross-sectional study | PD | **PD:**  N = 156 (68.3 ± 8.8)  M = 112  F = 44  **Controls:**  N = 110 (70.8 ± 8.8)  M = 48  F = 62 | **Processing of stools:**  Stool samples were collected from PD and controls patients and were assessed for stool microbiota composition  **Multiplexed immunoassays:**  Levels of immune factors were measured in undiluted stool homogenates using the V-PLEX Neuroinflammation Panel 1, which is divided into five panels of analyses: (1) Angiogenesis; (2) Chemokine; (3) Cytokine; (4) Proinflammatory; (5) Vascular injury | PD patients reported a greater incidence of intestinal disease and digestive problems than controls  Direct comparison of levels of stools analytes in patients and controls revealed elevated Flt1, IL-1a, and CXCL8 in patients’ stool  Sex, BMI, a history of smoking, and use of probiotics were found to strongly influence levels of stool analytes | This study did not account for other factors such as lifestyle factors (i.e., diet)  Relatively small sample size |
| **Nissen et al., (2021) [87]**  Tubingen, Germany  Cross-sectional study | iPD | **Experiment 1:**  **PD:**  N = 109 (66.6 ± 9.9)  M = 56  F = 53  **Controls:**  N = 44 (64.1 ± 10.7)  M = 25  F = 19  **Experiment 2:**  **PD:**  N = 106 (64.2 ± 10.7)  M = 61  F = 45  **Controls:**  N = 16 (56.9 ± 10.9)  M = 5  F = 11 | Demographics, clinical information, and assessments were collected  Analysed the monocyte-specific biomarker sCD163, the soluble form of the receptor CD163 in the cerebrospinal fluid (CSF) and serum in two experiments using ELISA  Association between CD163 and alpha-synuclein were done in vitro | Serum-sCD163 increased in late-stage PD and correlated with PD biomarkers alpha-synuclein, Tau, and phosphorylated Tau, whereas it inversely correlated with the patients’ cognitive scores, supporting monocyte involvement in neurodegeneration  Serum-sCD163 increased only in female patients  Alpha-synuclein activates macrophages and induces shedding of sCD163, which in turn enhances alpha-synuclein uptake by myeloid cells, potentially participating in its clearance | Cross-sectional design of this study  Low numbers of healthy controls  Potential confounding factors such as inflammatory disease with anti-inflammatory medications |
| **Nissen et al., (2022) [86]**  Tubingen, Germany  Cross-sectional study | Sporadic PD | **Cohort 1A**  **PD:**  N = 78 (66.6 ± 9.5)  **Controls:**  N = 28 (64.2 ± 7.4)  **Cohort 1B (Longitudinal sample):**  **PD:**  N = 7 | **Demographics and clinical assessments:**  UPDRS-III, H&Y  MoCA and/or MMSE  BDI-II  Sniffin’ Sticks  LEDD  **Blood sampling:**  Peripheral blood mononuclear cells (PBMCs) were collected, and isolated  **Flow Cytometry:**  PBMCs were stained for flow cytometry with a combination of nine fluorochrome-conjugated antibodies | Elevated frequencies and surface levels of migration- (CCR2, CD11b) and phagocytic- (CD163) markers, particularly on classical and intermediate monocytes in early PD  HLA-DR expression was increased in advanced stage PD, whereas TLR4 expression was decreased in females with PD  The disease-associated immune changes of CCR2 and CD11b correlated with worse cognition, whilst increased TLR2 expression was related to worse motor symptoms | Low sample size for both PD and healthy controls  Alternative separation based on PD subtypes or progression rates may challenge the early/late division based on disease duration |
| **Carlisle et al. (2021) [89]**  Alabama, USA  Cross-sectional study | PD | **PD:**  N = 18 (61.1 ± 10.5)  M = 11  F = 7  **Controls:**  N = 16 (65.4 ± 9.7)  M = 10  F = 6 | **Demographics and clinical assessments:**  H&Y, MDS-UPDRS, family history  MoCA  PDQ-39  **Sample collection:**  50mL of venous blood were collected and peripheral blood mononuclear cells (PBMCs) were isolated by low-density gradient centrifugation using lymphocyte separation medium  **Subsequent PBMCs and RNA analysis:**  Flow cytometry analysis for monocyte subsets  RNA isolation and quantitative RT-PCR  RNA-sequence library-prep  A computational model that incorporated case/control status, sex and interaction between case/control and sex was used | There was a striking effect of sex on monocyte gene expression  There was inflammatory activation of monocytes in females with PD, with enrichment of gene sets associated with interferon gamma stimulation, while in males with PD, the activation patterns were more heterogenous | This study pooled monocytes from individual subjects rather than utilizing single-cell sequencing approaches and thus, only responses averaged over a large number of monocytes were obtained  Rather small sample size  No consideration of other factors that might affect monocytes activation such as lifestyle factors |
| **Cortese et al. (2018) [74]**  Bergen, Norway  Historical cohort study | PD | **Exposed:**  N = 108,520  M = 71,885 (age onset = 73.1 ± 9)  F = 36,635 (age onset = 74.4 ± 8.5)  **Unexposed:**  N = 3,463,917  M = 1,690,260 (age onset = 70.4 ± 10)  F = 1,773,657 (age onset = 72.4 ± 9.7) | The historical cohort study included the entire Norwegian population alive at least 18 years old  The use of urate-lowering drugs, a marker of high urate levels/gout, from the Norwegian Prescription Database were retrieved and individuals from 01/01/2005 to PD onset, emigration, death, or end of follow-up on 31/12/2013 were followed | Exposure to urate-lowering drugs was associated with lower risk of PD and this association was more marked in males, compared to females (p = 0.61).  The association varied significantly by age among females (p = 0.01) with protective effect suggested only at higher age (above 70 years) when urate levels are higher than premenopausally, but not in males (p = 0.61). | 1. This study used only indirect measures of high urate levels |
| **Clinical Features: Motor Symptoms** | | | | | |
| **Lubomski et al. (2014) [96]**  Victoria, Australia  Cross-sectional survey study | iPD | N = 210 (69.1 ± 10.8)  M = 129  F = 81 | **Demographics and clinical assessments:**   1. Sociodemographic factors and clinical management information 2. PDQ-39 3. UPDRS-III 4. Medications: LEDD | **Clinical characteristics**   1. Males had significantly higher levels of motor impairment than females (higher scores on the UPDRS-III), even after controlling for age and disease duration 2. Females reported significantly lower mean daily LEDD than males 3. Males reported significantly more caregiver reliance than females 4. PDQ-39 showed males had lower QoL in activities in daily living, cognition, and communication | 1. Self-reporting nature of study design needs to be kept in mind 2. No consideration of several confounding factors such as psychiatric morbidities and effects of hormone replacement therapy 3. Selection bias since participants are drawn from specialist clinics |
| **Boccalini et al.** **(2022) [90]**  Milan, Italy  Data obtained from PPMI database | *De novo*, drug-naïve iPD | **PD:**  N = 286  M = 189 (62.32 ± 9.69)  F = 97 (61.35 ± 9.65)  **Controls:**  N = 73  M = 39  F = 34 | **Clinical assessments:**   1. Clinical classification method of motor subtypes 2. UPDRS and H&Y 3. Sleep: ESS & RBDSQ 4. Autonomic function: SCOPA-AUT 5. Olfactory function: UPSIT   **Cognitive assessment:**   1. Global cognition: MoCA 2. Memory: HLVT-R 3. Visuospatial function: JLO 4. Speed attention: SDMT 5. Working memory: LNS and semantic fluency   **Neurobehavioral assessment:**   1. GDS 2. STAI 3. QUIP   **MRI imaging**  **[123I]FP-CIT-SPECT** | I**n mild motor and intermediate subtypes:**   1. Males with iPD showed poor cognitive performances than females in the mild motor and intermediate subtypes 2. Females with iPD exhibited more severe anxiety symptoms in the mild motor and intermediate subtypes 3. Males with PD with motor impairment were found to have a lower dopamine binding in the putamen and more severe widespread connectivity alterations in the nigrostriatal dopaminergic pathways than females with PD   **Diffuse-malignant subtype:**   1. Males showed more severe motor impairment, consistent with a lower dopamine uptake in the putamen   **Other findings:**   1. Anxiety levels was associated with a lower dopaminergic binding in the amygdala only in females | 1. Study included a sample of healthy controls 2. A need for consideration of social factors such as gender disparities in education and occupation which might influence brain organization 3. [123I]FP-CIT binding in the extra-striatal region is also due to SERT besides DAT density |
| **De Micco et al., (2019) [92]**  Naples, Italy  Case-control, longitudinal study | Drug naïve iPD patients | **PD:**  N = 56  M = 30 (60.2 ± 6.6)  F = 26 (58.5 ± 10.2)  **Controls:**  N = 30  M = 15 (60.1 ± 6)  F = 15 (55.7 ± 5.6) | **Baseline:**   1. Clinical and imaging data in the morning, in the same day, in distinct sessions 2. One week after baseline, dopaminergic treatment was administered 3. PD patients followed for an observation period, lasting 2 years, undergoing an extensive clinical follow-up every 12 months   **Clinical evaluation:**   1. H&Y, UPDRS-III, IV 2. Treatment-related motor complications: Wearing-off questionnaire (WOQ-19) and Unified Dyskinesia Rating scale (UDysRS) 3. Medications: LEDD, LEDD-DA and total LEDD   **Imaging:**   1. Resting state fMRI | 1. Males with PD exhibited an abnormal spectral composition of the sensorimotor and dorsal attention networks in the slow-5 band, compared females with PD and healthy control 2. Females with PD showed an increased connectivity within the basal ganglia compared to males 3. Functional sensorimotor connectivity changes at baseline showed to be an independent predictor of disease severity at 2-year follow-up | 1. Small sample size 2. Careful clinical recruitment and accurate matching of patients’ subgroups. |
| **Porta et al., (2019) [91]**  Cagliari, Italy  Retrospective cross-sectional study | PD | N = 35  M = 18 (70.7 ± 5.8)  F = 17 (70.8 ± 8.2) | **Spatiotemporal and kinematic data collection:**   - - - 1. Kinematic and spatiotemporal parameters of gait was performed using a motion-capture system with 8 infrared cameras       2. Patients’ demographics, clinical information, and physical body measurements (i.e., pelvis height) were used to assist with gait assessment | Males and females with PD displayed similar spatiotemporal parameters except for step width (wider in males with PD)  Significant difference was found in ankle kinematics, in which females with PD exhibit increased ankle dorsiflexion through all the stance phase and mid to late swing phase and reduced plantarflexion at the stance-swing phase transition | Very small sample size  Selection bias as only PD patients with no support from assistive devices were included  No clarification at what stage of the disease (i.e., time from onset) the different kinematic alterations emerged |
| **Kolmancic et al., (2019) [93]**  Ljubljana, Slovenia  Clinical-based study | Newly diagnosed, drug naïve PD patients | N = 41  M = 22 (age at diagnosis = 63.32 ± 9.37)  F = 19 (age at diagnosis = 64.22 ± 12.24) | **Demographics and clinical assessments**:   1. Clinical severity: UPDRS 2. Lateralized motor sub score was calculated from the sum for bradykinesia, rigidity, and tremor for less and more affected side   **TMS:**   1. Indices: resting motor threshold (RMT), active motor threshold (AMT), input-output (IO) curve, 1-mV motor-evoked potential (MEP), short interval intracortical inhibition (SICI), intracortical facilitation (ICF), cortical silent period (CSP) 2. Excitatory paired associative stimulation (PAS25): Assessment of its effect on corticospinal excitability   **Electromyographic (EMG) Recordings** | **Demographics findings:**   1. No significant differences between sexes were found in age, age of diagnosis, disease duration, and total or lateralized motor score   **TMS findings:**   1. Males had disturbed interhemispheric balance of motor thresholds, caused by decreased resting and active motor thresholds in the more affected hemisphere 2. In females, short interval intracortical inhibition was more effective than in males, in both hemispheres 3. Females exhibited a preserved physiological focal response to sensorimotor plasticity protocol while males exhibited an abnormal spread of protocol effect | 1. Very small sample size 2. Only a brief assessment of motor symptoms was evaluated – what does the observed brain activity means in terms of behaviour is unclear |
| **Rusz et al., (2022) [256]**  Prague, Czech Republic  Part of a longitudinal project BIO-PD (biomarkers in PD) | *De novo* PD patients | **PD:**  N = 100  M = 60 (60.8 ± 11.7)  F = 40 (61.2 ± 13.3)  **Controls:**  N = 100  M = 60 (61.1 ± 11.4)  F = 40 (61 ± 13.2) | **Clinical examination:**   1. Structured clinical interview on personal and medical history, history of drug and substance intake and current drug usage 2. UPDRS-III 3. MoCA 4. Perceptual speech severity: UPDRS-III it 5. Self-reported severity of speech impairment: UPDRS-II   **Dopamine Transporter Imaging:**   1. DAT-SPECT [123]   **Speech examination:**   1. Speech recordings in a single session using three vocal tasks   **Acoustic speech analysis:**   1. Quantitative acoustic vocal assessment of 10 distinct speech dimension related to phonation, articulation, prosody, and speech timing | 1. Prevalence of speech abnormalities: 56% for male and 65% for female 2. Females exhibited better performance in consonant articulation, voice quality, and pauses production 3. Males exhibited better performance in loudness variability 4. In males, the extent of monopitch was correlated to nigro-putaminal dopaminergic loss in males whilst severity of imprecise consonants was associated to cognitive deficits in females 5. No sex specific speech dysfunction in *de novo* PD was found | 1. Relatively small sample size 2. Not thorough measurement of different domains of cognition |
| **Bjornstad et al., (2016) [160]**  Norwegian ParkWest project, Norway  Prospective, population-based multicentre, longitudinal cohort study | Newly diagnosed, untreated PD | N = 189 (67.7 ± 9.3)  M = 114  F = 75 | **Demographics and clinical assessments:**   - - - 1. General medical and semi-structured interviews to obtain medical and drug history  1. UPDRS-III and H&Y 2. Non-motor features: MMSE, MADRS, FSS, PDSS, ESS, SAS 3. LEDD and other medications or surgeries 4. PD patients were followed at baseline for a period of 5 years, at 6-month intervals. | - - - 1. Besides higher motor severity predicting both motor fluctuations (p = 0.016) and dyskinesias (p < 0.001), lower age at diagnosis predicted motor fluctuations (p = 0.001), whereas female sex predicted dyskinesias (p = 0.001)  1. Actual levodopa dose at onset of motor fluctuations (p = 0.037) or dyskinesias (p < 0.001), rather than initial treatment with levodopa (p > 0.1) independently predicted development of motor complications | - - - 1. Relatively small sample size  1. No consideration of other lifestyle factors that might affect motor complications such as diet and exercise |
| **Colombo et al., (2015) [100]**  Multi-centres, Italy (Parkinson’s disease DEEP observational study)  Cross-sectional study | Non-demented, under levodopa (LD) and/or dopamine agonists (Das) therapy for >1 year before study screening | N = 617  M = 381 (66.6 ± 9.4)  F = 236 (67 ± 8.9) | **Demographics and clinical assessments:**   1. Structured interview and by examination 2. Medications: LEDD 3. UPDRS and H&Y 4. Wearing off: WOQ-19 | 1. Female had a higher prevalence of WO according to neurologists’ judgments and the WOQ-19 analysis 2. Female experience >1 motor symptom in 72.5% versus 64.0% in males and >1 non motor symptoms in 44.5% versus 36.7% in males | 1. Post-hoc analysis not originally intended to assess for sex differences 2. Patient selection bias and no analysis of non-APD medication 3. No assessment of contribution of other symptoms |
| **Cho et al., (2019) [257]**  Chonnam, South Korea  Cross-sectional study | *De novo* PD | **Coffee drinkers**:  N = 204 (64.57 ± 9.83)  M = 120  F = 84  **Non-coffee drinkers:**  N = 80 (68.79 ± 8.24)  M = 27  F = 53 | **Demographics and clinical assessments:**   - - - 1. Patients underwent a thorough neurological examination and detailed demographics, and clinical history were obtained       2. Disease severity: Modified H&Y, UPDRS-III, ADL       3. Postural instability and gait disturbance: PIGD and AR       4. Non-motor symptoms: MMSE, MoCA, NMSS, BDI       5. Past and present coffee consumption data were obtained via semi-structured interview | Coffee drinkers were predominantly male and early symptom onset; they were also younger, reported more years in formal education, and had better motor and non-motor scores than non-coffee drinkers  After adjustments, coffee drinkers had lower tremor scores than did non-coffee drinkers, and coffee consumption was related to tremors in a dose-dependent manner  The dose-dependent relationship between coffee consumption and tremor severity was significant only in males | There were significant differences between coffee drinkers and non-coffee drinkers in demographics background such as age at symptom onset, sex, and formal education  This study only investigated the association between coffee consumption and motor symptoms only in *de novo* PD patients, without examining control or advanced PD group  This study did not include any other information about the consumption of other caffeine-containing beverages |
| **Kim et al., (2018) [101]**  Seoul, Korea  Retrospective cohort study using PPMI | *De novo* PD | N = 390 | **Clinical assessments:**  Patients were followed up to 3-month intervals in the first year, 6-month intervals in the subsequent four years and 12-month intervals in the last three years  UPDRS-III  PIGD  MoCA  **DAT Imaging** | During a median follow-up period of 4 years, 36.7% of patients with PD developed FOG.  Multivariate Cox regression analyses showed that DAT uptakes in the caudate nucleus (p = 0.001) and putamen (p = 0.027) predicted the development of FOG  Male sex, higher postural instability, and gait difficulty score, and a lower MoCA score were also significant predictors of FOG. | Dopaminergic neurons may not degenerate in a linear pattern, and the initial DAT scan may not reflect a precision prediction of disease progression  FOG in the ON- and OFF-medication states is regarded to have different pathomechanisms, however, this study could not distinguish FOG depending on the medication state |
| **Ou et al., (2018) [102]**  Sichuan, China  Prospective Study | PD | N = 263 (62.3 ± 11.8)  M = 148  F = 115 | **Demographics and clinical Assessments:**  To obtain more positive outcomes, the planned final visit was set at 3 years. Additionally, a visit after one-year of enrolment is also conducted.  Face-to-face interview or telephone call was conducted during the one-year time interval  Demographic and clinical data (i.e., LEDD, disease duration, motor complications) were obtained at baseline  UPDRS-III, H&Y  NMSS  FAB, MoCA  HAMD, HAMA | The following variables including the proportion of males; age; disease duration; LEDD; UPDRS-III scores; H&Y stage; the percentages of festination, freezing of gait and falls; and the sub scores (e.g., “problems having sex”) and frequencies (e.g., “forget to do things”) of the NMSS were significantly higher in patients with camptocormia compared to those variables in patients without camptocormia (p<0.05).  The presence of camptocormia was associated with male sex (p = 0.001), a higher UPDRS-III scores (p = 0.001), a higher sexual dysfunction score (p = 0.038) and a lower orientation score (p = 0.018). | Although the prevalence of camptocormia in PD is low, the less positive outcome of camptocormia in the current study may also be attributed to the relatively short follow-up period  There are other confounding factors that may affect results  This study did not identify the risk factors of camptocormia in patients with different medication states (“OFF” or “ON”), so the role of levodopa therapy in the development of camptocormia cannot be identified |
| **Clinical features: Non-motor Symptoms; Cognition** | | | | | |
| **Bakeberg et al., (2021) [42]**  Australia: Australian Parkinson’s Disease Registry  Cross-sectional, longitudinal study | iPD | N = 392 (age onset = 56.9 ± 10.2)  M = 253  F = 139 | **Demographics and Clinical Assessments:**   - - - 1. Medication dosage, and other disease-related features.       2. Motor Symptoms: UPDRS- III and H&Y       3. Cognition: ACE-R   **For longitudinal study:**   1. A subset of participants (n=127, 62% males, 37% females) were followed up between 1 and 7 years. | **Cross sectional study findings:**   1. Males performed worse on global cognition, memory, and fluency domains   **Longitudinally study findings:**   1. Males had greater reduction in global cognition and language while females regressed more in attention/orientation, memory, and visuospatial domain, despite starting off with higher baseline scores (Selective decline) 2. Follow-up: Higher number of males fulfilled criteria for mild cognitive impairment or PD dementia | 1. Home-based data obtained from 3 different movement disorder centres across Australia: scoring variability 2. Excluded individuals with more advanced PD and dementia – likely to contribute to lower mean age of symptom onset and higher cognitive score at onset – not representative of a community-based sample 3. Attrition for longitudinal study – degree of bias |
| **Reekes et al., (2020) [103]**  California & Louisiana, USA  Cross-sectional, retrospective study | Non-demented Parkinson’s patients | **PD without Dementia:**  N = 84  M = 46 (67.34 ± 6.45)  F = 38 (66.53 ± 5.97)  **Controls:**  N = 59  M = 32  F = 27 | **Demographics, disease state data, motor performance, battery of neuropsychological tests:**   1. MMSE 2. ESS 3. NAART-R 4. GDS 5. LEDD 6. UPDRS and H&Y   **Executive functions:**   1. Delis-Kaplan Executive Function Scale (D-KEFS) Verbal Fluency (VF) Test 2. D-KEFS Colour Word Interference Test (CWI) 3. D-KEFS Trail Making Test (TMT) 4. WAIS-III Digit Span 5. SDMT | **Demographics findings:**   1. No significant differences for age, years of education, global cognition, daytime sleepiness, predicted premorbid IQ, UPDRS score, PD phenotype or disease duration   **Cognition findings:**   1. Males with PD exhibited worser performance for executive function and processing speed, although this difference was not found in controls 2. Males with PD performed significantly poorer in verbal fluency (category fluency, category switching and category switching accuracy), Colour Word Interference (Inhibition) and speed of processing (SDMT) 3. Steeper slope of disease progression in males with PD | 1. Attempted to explain findings with phenotypic profile (i.e., PIGD/TD phenotype), however, proportion of phenotypes were similar between sexes. 2. Sample size small – speculative to make a definitive statement on sex-phenotype interaction 3. This study included controls |
| **Cholerton et al., (2018) [121]**  Multi-states, USA  Prospective cohort study | PD | N = 567 (Total PD-MCI, Progressed = 71.2 ± 9.1; Total PD-MCI, Stable = 66.9 ± 8.0) | **Cognitive tasks (primary variables):**   1. MoCA 2. HVLT 3. LNS 4. TMT (Part B minus Part A) 5. Judgement of Line Orientation: measure of visuospatial ability 6. Semantic and phonemic verbal fluency   **Secondary variables:**   - - - 1. History of cardiovascular risk and hypertension (Hachinski Ischemic Index), head injury and past alcohol and tobacco use       2. Genomic DNA were obtained; Genes associated with PD cognitive function: GBA gene, APOE4 | 1. Processing speed and working memory were associated with conversion to PDD among those with PD-MCI at baseline, over and above demographic variables 2. The primary predictive factor in the transition from no cognitive impairment to PD-MCI or PDD was male sex and males progressed more rapidly than females 3. Among females with shorter disease duration, progression was slower than males, and poor baseline performance on semantic verbal fluency was associated with shorter time to cognitive impairment in females but not in males | - - - 1. Relatively large sample size  1. This study is unable to follow the natural history of cognitive impairment from the time of first PD diagnosis in all participants |
| **Gao et al., (2015) [131]**  Guangdong, China  Cross-sectional, observational study | PD | N = 311 (60.85 ± 11.35)  M = 172 (60.91 ± 11.93)  F = 139 (60.78 ± 10.63) | **Demographics and clinical assessments:**   1. Age, disease duration, education levels 2. UPDRS-III and H&Y 3. ADL 4. HAMD 5. HADS   **Cognitive Assessment:**   1. MMSE 2. MoCA 3. WAIS-RC 4. WMS-RC | 1. Males with PD exhibited a significantly higher MoCA score than females with PD (adjusted p < 0.05) 2. Males with PD displayed a better performance on visuospatial function, naming, and abstraction (p < 0.05) 3. On WAIS-RC, females with PD had lower scores in information, vocabulary, picture completion and picture arrangement (p < 0.05) 4. Cognitive disturbances were more prevalent and severe in females with PD | 1. Males with PD group had more years of education than female group, although this was adjusted 2. No control groups were used 3. Relatively small sample size |
| **Yang et al., (2018) [104]**  Fudan, China  Cross sectional, case-controlled clinical study | Non-demented PD | N = 60 (age onset = 54.97 ± 10.42)  M = 30 (age onset = 54.4 ± 11.38)  F = 30 (age onset = 55.53 ± 9.53) | **Demographics and clinical:**   1. H&Y, UPDRS III, LEDD 2. BDI 3. MMSE   **Cognitive domains/assessments:**   1. Verbal memory: Auditory verbal learning test (free recall testing with interference 2. Spatial processing ability: Rey-copy (Copy one complex line-drawing figure without reminding later recall), Clock Drawing test 3. Non-verbal memory: Rey-delayed recall 4. Language: BNT, Verbal fluency test (VFT) 5. Attention/Executive function: SDMT, TMT, Stroop Test | 1. As compared to controls, both males and females showed worse performance in AVLT and Symbol Digit Modality test 2. Males performed worse on Verbal Fluency Test-Animals, Cities and Alternative 3. Females performed worse on Clock Drawing Test, Boston Naming Test and Trails making Test A | 1. Small sample size 2. Lack of consideration for disease onset, progression, and ageing factors on cognition 3. Lack of consideration for the influence of medications on cognition – despite ruling out anticholinergic medication and levodopa equivalent dose, dopaminergic medications still influence development of neurodegeneration |
| **Heller et al., (2018) [105]**  Aachen, Germany  Cross-sectional, case-controlled study | Non-demented Parkinson | **PD:**  N = 51  M = 26 (63.9 ± 8.4)  F = 25 (64 ± 10)  **Controls:**  N = 44  M = 25  F = 19 | **Demographics and Clinical Assessments:**   1. German version of the Structured Clinical Interview for DSM-IV 2. MoCA 3. Attention and working memory: Digit span (backwards and forward) from Weschler Memory Scal 4. BDI-II and BAI 5. Alexithymia: Toronto Alexithymia Scale 6. UPDRS I-IV and H&Y 7. Non-Motor Symptoms 8. Medications: LEDD 9. Female: Hormonal factors   **Emotion recognition and fMRI paradigm:**   1. Benton facial recognition test 2. Event-related fMRI | **Cognition findings:**   1. No sex differences were found in cognition   **Emotion recognition:**   1. Males exhibited poorer performance in recognition of emotion anger, with diminished neural response to facial expressions in the putamen and insula 2. Fear recognition positively correlated with oestrogen levels in females 3. Genetic factor (Val158Met COMT polymorphism) implicated in fear recognition in PD patients | 1. Relatively small sample size 2. No consideration of influence of motor symptoms/disease progression/phenotypes on emotion recognition 3. Highly variable disease duration in PD sample 4. fMRI: used static images during behavioural task but dynamic stimuli were used to assess the neural correlates of emotion processing – somewhat different modality |
| **Kim et al., (2021) [65]**  Incheon, Korea; Data obtained from the PPMI database  Longitudinal study | *De novo* PD | N = 361 (61.4 ± 9.8)  M = 238  F = 123 | **Demographics, clinical and genetic assessments:**   - - - 1. Patients were classified into the following groups: APOE e4+/M (n = 65), APOE e4-/M (n = 173), APOE e4+/F (n = 25) and APOE e4-/F (n=98)       2. Cognitive decline: MoCA, assessed annually over a 5-year period | 1. Males with APOE4 had a steeper rate of cognitive decline than those without APOE4 2. No significant interaction between APOE4 and time on longitudinal MoCA performance in females 3. The main effect of APOE4 on the change in the MoCA score was not significant for either males or females 4. When the data from both males and females were used, the APOE4+/M group exhibited a steeper rate of cognitive decline than did the APOE4+/F and APOE4-/F groups | - - - 1. Relatively small sample size, especially when parsed into the different sex and status of APOE e4 status       2. MoCA is a global cognitive assessment, which might not be able to capture specific cognitive domains |
| **Oltra et al., (2022) [107]**  Data obtained from the PPMI database  Cross-sectional study | *De novo* PD | **Total PD with pRBD:**  N = 79  M = 54 (64.7 ± 7)  F = 25 (63.5 ± 7.5)  **Total PD without pRBD:**  N = 126  M = 73 (63.2 ± 7.4)  F = 53 (60.9 ± 7.4)  **Controls:**  N = 69 | **Clinical and Neuropsychological assessments:**   1. MoCA 2. Phonemic and semantic verbal fluency tests 3. SDMT, LNS, JLO, HVLT-R 4. UPDRS and H&Y 5. GDS 6. UPSIT-40 7. RBDSQ   **MRI:**   1. Cortical thickness | **MRI findings:**   1. Males showed greater global cortical and subcortical grey matter atrophy than females in the PD-pRBD group 2. Significant group-by-sex were found in the pallidum   **Cognitive findings:**   1. Significant group-by-sex interactions were found in the MoCA and Symbol Digits Modalities Test (SDMT) 2. Males performed worse than females in MoCA, phonemic fluency and SDMT in the PD-pRBD group 3. Male sex is related to increased cognitive impairment and subcortical atrophy in *de novo* PD-pRBD | 1. Large sample size 2. Diagnosis of RBD not made by PSG, only RBDSQ screened – false positive discovery rate 3. PPMI data acquired from multicentre cohort with variability in MRI acquisition protocol 4. Healthy controls might not be an appropriate control group since isolated RBD is rare |
| **Bayram et al., (2020) [106]**  Data obtained from the PPMI database  Longitudinal study | *De Novo* PD | **Cognitively intact PD:**  N = 257  M = 157 (61.33 ± 10.45)  F = 100 (60.03 ± 9.4)  **Cognitive impaired PD:**  N = 167  M = 167 (63.15 ± 8.9)  F = 45 (62.2 ± 10.03)  **Controls:**  N = 140  M = 84  F = 56 | **Demographics and clinics information:**   1. GDS 2. STAI 3. ESS 4. UPDRS III   **Cognitive assessments:**   1. MoCA 2. Hopkins Verbal Learning Test-Revised: Verbal learning/memory 3. Benton Judgment of Line Orientation 4. SDMT 5. Weschler Memory Scale-III Letter Number Sequencing 6. Animal naming | 1. Females exhibited better performance on verbal learning and memory, men have a better performance on visuospatial functioning 2. Controls exhibited significantly better performance on general cognitive abilities, verbal learning memory, visuospatial functioning, and processing speed measures than PD-MCI group 3. PD-NC performed better on general cognitive abilities, verbal learning and memory and visuospatial functioning than PD-MCI group | 1. Relatively large sample sizes 2. Use of PPMI database: not much elaboration of cognitive abilities, multicentre cohorts’ variability in data collection |
| **Oltra et al., (2022) [108]**  Data obtained from PPMI database  Cross-sectional study | *De Novo* PD patients | **PD:**  N = 205  M = 127 (63.8 ± 7.24)  F = 78 (61.76 ± 7.5)  **Controls:**  N = 69  M = 40 (64.05 ± 7.11)  F = 29 (60.55 ± 5.86) | **Clinical and neuropsychological assessments:**   1. UPDRS-III and H&Y 2. RBDSQ 3. MoCA 4. Phonemic and semantic verbal fluency 5. SDMT, LNS, JLO, HVRT-R   **MRI:**   1. T1-weighted MRI data | 1. Males with PD exhibited greater motor and RBD symptomology more than PD females 2. Males with PD also showed cortical thinning in postcentral and precentral regions, greater global cortical, and subcortical atrophy, and smaller volumes in thalamus, caudate, putamen, pallidum, hippocampus, and brainstem compared to females 3. Males with PD performed significantly worse than females with PD in global cognition, immediate verbal recall, and mental processing speed 4. In both healthy and PD male group, they performed worse than females in semantic verbal fluency and delayed verbal recall 5. Females in PD and healthy control groups performed worse than males in visuospatial function | 1. No consideration of other factors such as environment, lifestyle and diet on brain atrophy and functional outcomes in PD 2. PPMI study includes multisite data including varying field strength of MRI acquisition |
| **Song et al., (2014) [137]**  Beijing, China  Data from multi-centre cohort of Chinese Parkinson Study Group  Cross-sectional study | *De Novo* PD | N = 428 (age onset = 58.75 ± 10.55)  M = 258 (age onset = 59.39 ± 10.64)  F = 170 (age onset = 57.78 ± 10.38) | **Motor Measurements:**   1. UPDRS III 2. Motor subtypes: tremor dominant (TD), PIGD and intermediate type   **Non-motor measurements:**   1. Depression: CES-D 2. PSQI 3. MMSE 4. ADAS-Cog 5. Constipation and Fatigue 6. UPDRS I-II | **Motor findings:**   1. No gender differences found for UPDRS scores, four cardinal motor signs or motor subtypes   **Non-motor findings:**   1. Females were more likely to exhibit increased depressive symptoms 2. Males performed better on the MMSE scores but lower scores for identification in the ADAS-cog | 1. Relatively large sample size 2. Only focuses on several specific non motor symptoms rather than extensive symptoms 3. Only used global cognition tasks rather than specific cognitive domains 4. Selection bias |
| **Picillo et al., (2022) [95]**  PPMI database  Longitudinal, case-controlled study | *De novo*, untreated PD | **PD:**  N = 423  M = 277 (62.2 ± 9.7)  F = 146 (60.7 ± 9.6)  **Controls:**  N = 196  M = 126  F = 70 | **Clinical and neuropsychological assessments:**   1. UPDRS (I to IV) and H&Y 2. Medication history 3. Classification of subtypes (tremor dominant or non-TD; postural instability, and gait disorders and indeterminate) 4. UPSIT 5. SCOPA-AUT 6. GDS, STAI and QUIP 7. RBDSQ and ESS   **Cognitive Assessments:**   1. MoCA 2. HVLT 3. Benton Judgment of Line Orientation 4. SDMT 5. Letter Number sequencing 6. Semantic fluency   **Dopamine SPECT Imaging**: Screening at Year 1, 2 and 4 to assess degree of presynaptic dopaminergic dysfunction  **Biological samples:**   1. CSF biomarkers 2. Total tau (T-tau) 3. Phosphorylated tau (P-tau) 4. Unphosphorylated total a-synuclein | **Demographic findings:**   1. No difference in sex in age, education, ethnicity, race, family history of PD, duration of PD, age of diagnosis, or ApoE4 genotype 2. Females had significantly lower weight, BMI, and VRFS 3. Males were more likely to have the right side more affected   **Motor and non-motor symptoms findings:**   1. Males had greater longitudinal decline in self-reported motor and non-motor symptoms such as daily living, (males had a yearly increase of 1.27 UPDRS-II compared to female 0.7) 2. Males had increased longitudinal progression in clinical-assessed motor symptoms when on medication and required higher dopamine dosages compared to females 3. No significant changes in disease milestone and longitudinal changes in CSF biomarkers and DatScan between sexes | 1. PPMI population: Participants enrolled in the PPMI represent a highly selected population with less baseline disability than general 2. PD progression may not be linear 3. No accountability of pre-, post-, or peri menopause states |
| **Shin et al., (2017) [127]**  Multi-states, USA  Cross-sectional, descriptive survey | Community-dwelling people with PD, with no known dementia | N = 141 (69.7 ± 8.21)  M = 84 (70.57 ± 8.64)  F = 57 (68.53 ± 7.45) | **Demographics and other information:**   1. Age, sex, race/ethnicity, marital status, years of education, and annual household income 2. General health: Difficulty of performing daily activities, other chronic diseases, health care spending, health insurance, and visits to emergency room 3. PD specifics: duration, age onset, motor, and non-motor symptoms | 1. Males reported increased rigidity, speech problems, sexual dysfunction, memory problems and socializing compared to females 2. No other sex differences were found | 1. Relatively small sample size 2. Participants were homogenous in terms of race/ethnicity and education – no consideration 3. Other confounding variables: age, duration of PD, PD progression |
| **Fengler et al. (2016) [132]**  Multicentre, Germany  Prospective, observational cohort study using LANDSCAPE database | PD | N = 656  M = 445 (67.9 ± 7.8)  F = 211 (67.2 ± 7.8) | **Demographics and neuropsychological assessments:**   - - - 1. Patients’ demographics, clinical, neuropsychological test battery, blood tests and neuroimaging procedures were obtained from the LANDSCAPE study  1. MMSE, PANDA, CERAD-Plus, Modified Card Sorting Test, Stroop Interference test, BTA | 1. Females with PD were superior in verbal memory (word list learning, p = 0.02; recall, p = 0.03) than PD males 2. Males with PD were significantly better in visuoconstruction (p = 0.002), and figural memory (p = 0.005) 3. Gender-corrected Z scores showed that males were superior in verbal memory (word list learning, p = 0.02; recall, p = 0.02; recognition, p = 0.04), while no differences were found for visuospatial tests | 1. Neuropsychological assessments test battery was limited as visuoconstruction and figural memory was assessed with one test only 2. Longitudinal data were not available 3. Selection bias, severely demented PD patients were not included |
| **Augustine et al., (2015) [258]**  USA, Canada  Cross-sectional, clinical trial study | Early treated PD | N = 1741  M = 1123 (disease duration = 3.2 ± 2)  F = 618 (disease duration = 3.3 ± 2.3) | **Demographics and clinical assessments:**   - - - 1. Age at onset, age at PD diagnosis, age at randomization, years since symptoms onset, years since PD diagnosis etc  1. Clinical features: retrospective patient-reported symptoms at the time of diagnosis (motor symptoms) and symptoms present at the time of randomization (motor symptoms, non-motor symptoms, and daily functioning) | - - - 1. There were no differences found in mean age at PD onset, age at PD diagnosis, age at randomization, motor symptoms, or daily functioning between males and females  1. Differences in non-motor symptoms were observed, with females displaying better performance compared to males on SCOPA-COG and Symbol Digit Modality measures. | - - - 1. Relatively large sample size  1. This study used a broad range of well-validated assessments |
| **Szewczyk-Krolikowski et al., (2014) [126]**  Oxford, United Kingdom  Cross-sectional, cohort study using the Oxford Parkinson Disease Center (OPDC) discovery cohort | PD | **PD:**  N = 490 (67.9 ± 9.3)  M = 305  F = 185  **Controls:**  N = 176 (64.3 ± 9.1)  M = 64  F = 112 | 1. Clinical features, demographics, medical characteristics, non-motor and motor symptoms were obtained from the OPDC cohort 2. Disease features were stratified according to age and compared between genders, controlling for effects of common covariates | 1. For motor symptoms, a pattern of increased severity and greater symptom symmetricity in the face, neck, and arms in males with females having more postural problems 2. Males with PD had more cognitive impairment, greater rate of RBD, more orthostatic hypotension and sexual dysfunction | 1. Cross-sectional nature may bias some variables through retrospective reporting 2. Age and gender were investigated in univariate comparisons 3. Relatively small sample size |
| **Pigott et al., (2015) [125]**  Pennsylvania, USA  Longitudinal study | iPD | N = 141 (68.6 ± 7)  M = 89  F = 52 | **Clinical and neuropsychological assessments:**   1. PD patients were assessed at baseline, and for a minimum of 2 years and up to 6 years 2. UPDRS-I, III, H&Y 3. GDS 4. Cognition: DRS-2, Letter-Number sequencing, and phonemic verbal fluency (FAS), HVLT-R, Judgement of Line Orientation and Language, BNT, and semantic verbal fluency (animals) | 1. Predictors of future cognitive decline were male sex (p = 0.02), higher UPDRS motor score (p ≤ 0.001) and worse global cognitive score (p < 0.001) | 1. Small sample size 2. Selection bias as samples was 99% Caucasian with a narrow age range 3. Only used brief, self-reported measures of apathy, psychosis, and depression 4. Not consideration of other clinical factors of cognitive decline such as gait impairment and APOE4 status |
| **Clinical Features: Non-motor symptoms; Sleep** | | | | | |
| **Ratti et al., (2012) [110]**  Pavia, Italy  Cross-sectional survey | PD, PDD, DLB | **PD, PDD, DLB:**  **Final sample**:  N = 80 (66.6 ± 10)  M = 50  F = 30 | **Clinical and neuropsychological assessment**:   - - - 1. Clinical interview and neurological examination       2. UPDRS-III & H&Y       3. Sleep: Semi-structured questionnaire-based interview, ESS       4. Cognition: MMSE, tests evaluating attention, executive functions, verbal fluency, short-term verbal and spatial memory, long-term verbal memory, non-verbal logic, visual search abilities and visuo-constructional skills       5. Medications: LEDD   **Sleep monitoring and scoring:**   - - - 1. Video-PSG       2. Sleep indices: PLMs, and respiratory events       3. Quantification of sleep-related motor behaviour | - - - 1. SEBs consisting of RBD or occurring on arousal from NREM, or REM sleep were globally more frequent in the dementia group (PDD/DLB) than in PD group, the difference being statistically significant for arousal-related episodes, while a trend emerged for RBD  1. Male sex, daytime sleepiness, higher motor impairment, and lower mini-mental score were significantly more frequent with the occurrence of abnormal sleep-related motor-behavioural episodes | - - - 1. Relatively small sample size  1. Other factors that could potentially affect sleep such as lifestyle factors were not included |
| **Liu et al., (2021) [109]**  Taiwan, China  Cross-sectional study | iPD | N = 586  M = 347 (65.7 ± 10.2)  F = 239 (64.1 ± 9.03) | **Demographics and clinical assessments:**   1. Patients divided into EOPD (Early onset PD; <50 years) or LOPD (AAO >50 years) 2. Sex, age, AAO disease duration, LEDD, motor subtype, motor fluctuation, and dyskinesia 3. UPDRS-III and H&Y 4. Depression: HAMD   **Sleep assessments:**   1. RBDSQ 2. ESS 3. SCOPA-DS 4. PSQI | 1. PD patients with different sexes and AAO show a differential clinical phenotype 2. Prevalence of PD in males is 1.45 times higher than of females 3. EDS was more prominent in males than females 4. LOPD patients had a higher probability of poor night-time sleep quality 5. Using a multivariate logistic regression analysis, male sex, disease duration and depression were risk factors for EDS | 1. Healthy control group not included 2. Selection bias as only subjects with H&Y score less than 2.5 was included 3. Not use of MLST or PSG, only self-rated scales were used – risk may be underestimated |
| **Bjørnarå et al., (2013) [139]**  Drammen, Norway  Cross-sectional study | iPD | N = 107  M = 65 (68 ± 9)  F = 42 (68.6 ± 7.3) | **Demographics and clinical assessments:**   - - - 1. MDS-UPDRS, H&Y       2. Medications: LEDD       3. HAD       4. Sleep: PDSS, ESS   **Assessment of RBD:**   - - - 1. PSG  1. RBDSQ | - - - 1. Males had more fights (96% versus 54% p < 0.001), violent behaviour (71% versus 39%, p = 0.04) and awakening by own movements (89% versus 62%, p = 0.04).  1. A higher proportion of females experienced disturbed sleep (85% versus 32%, p = 0.02) 2. The frequency of pRBD was 31% in females and 43% in males (p = 0.2), total frequency of 38%. | - - - 1. Relatively small sample size  1. No consideration of other factors that might affect sleep such as lifestyle factors |
| **Symptomology: Non-motor symptoms; Others** | | | | | |
| **Solla et al., (2020) [111]**  Cagliari, Italy  Case-control, clinical study | PD | **PD:**  N = 99  M = 57 (68.5 ± 7.5)  F = 42 (69.6 ± 8.1)  **Controls:**  N = 69  M = 31  F = 38 | **Demographics and clinical assessments:**   1. Age, weight, height, BMI, smoking status, LEDD 2. MoCA 3. Fatigue and apathy: PFS and SAS 4. H&Y and UPDRS-III 5. Olfactory function: SSET | 1. Males with PD scored significantly lower on odour discrimination, identification, and threshold-discrimination-identification score than females, but not odour threshold 2. Using the multivariable linear regression analysis, only significant predictors of TDI were sex and apathy | 1. Potential bias caused by assessors being unblinded to the case or the control status 2. Referral bias as patients were recruited from a clinic 3. Use of healthy controls |
| **Nicoletti et al., (2017) [112]**  FRACAMP study; large multicenter case-control study in Southern Italy | PD | **PD:**  N = 585 (66.8 ± 9.8)  M = 348  F = 237  **Controls:**  N = 481 (63.4 ± 10.1)  M = 168  F = 313 | **Demographics and clinical assessments:**   1. Face-to-face interview 2. Neurological examination 3. MMSE 4. HAMD 5. SAPS 6. Standardised questionnaire on sleep, gastrointestinal, urinary, and sexual disturbances | 1. Females with PD displayed a significantly higher rates of depression and urinary disturbances than males 2. A similar rate among females and males with PD were obtained for hallucination, cognitive impairments, and sleep disorders 3. As compared to controls and after stratifying for sex and age, PD males exhibited a stronger positive significant association with almost all NMS except for urinary disturbances, as compared to females with PD 4. Strongest association among males with PD were cognitive impairment (adjusted OR 5.44 for males and 2.82 for females) and depression (adjusted OR 30.88 for males and 12.72 for females) | 1. Differences in NMS assessments makes it tedious for direct comparison 2. Possible interviewer bias due to clinical characteristic of study population 3. Selection bias |
| **Hu et al., (2018) [113]**  Sichuan, China  Cross sectional observational study | Drug-naïve PD | N = 552 (58.1 ± 12.4)  M = 275 (57.9 ± 13.7)  F = 294 (58.2 ± 11) | **Detailed clinical and demographic information from self-reported history and neurological examination:**   1. Patients were divided into early onset and late onset 2. UPDRS-III and H&Y 3. Executive function: FAB & MoCA 4. Depression: HDRS 5. Anxiety: HADS 6. Non-motors symptoms: Chinese NMSS, 9 domains 7. QoL: Chinese version of PDQ-39 | 1. NMS is common in non-treated PD patients, however heterogeneous symptoms between sexes and onset age group 2. Males reported a significant increase in urinary and sexual dysfunction, whilst females had increased frequencies of sleep/fatigue and mood/apathy 3. Patients with late-onset PD showed increased frequencies of attention/memory, gastrointestinal, urinary, sexual dysfunction as compared to early onset PD | 1. No use of healthy controls 2. Cognition and NMSS measures were limited to global measurement instead of specific ones 3. Relatively large sample size |
| **Picillo et al., (2013) [114]**  Naples, Italy  Retrospective study | PD | **PD:**  N = 200 (61.3 ± 8.8)  M = 126 (61.2 ± 9)  F = 74 (61.5 ± 8.6)  **Controls:**  N = 93 (60.4 ± 7.7)  M = 60 (60.7 ± 7.8)  F = 33 (59.8 ± 7.6) | **Demographics and clinical assessments:**   1. Detailed clinical and demographics were obtained from patients’ history and neurological examination 2. UPDRS-III, H&Y 3. NMSQuest | 1. Males with PD reported of problems having sex and taste/smelling difficulties significantly more than females with PD 2. Males with PD complained more frequently of dribbling, sadness/blues, loss of interest, anxiety, acting during dreams, and taste/smelling difficulties as compared to healthy controls 3. Females with PD reported more frequent experience of loss of interest and anxiety as compared to healthy controls | 1. Relatively small sample size 2. Lack of severity assessment of NMS 3. Need a more comprehensive assessment of NMS symptoms |
| **Zhu et al., (2017) [136]**  Nanjing, China  Cross-sectional study | Non-Demented PD | N = 519 (65.35 ± 10.19)  M = 326  F = 193 | **Demographics and clinical assessments:**   1. Standardized questionnaires on demographics information (e.g., age, sex, age of onset, education level) 2. UPDRS-III and H&Y 3. NMS: NMS-Quest 4. MMSE 5. Sleep quality: PDSS 6. Depression: HAMD | 1. Significant correlation was found between total HAMD score and sex, PD duration, UPDRS-III, H & Y, PD-NMS, PDSS and MMSE were found 2. Female patients had significantly higher total HAMD score and domains of anxiety/somatisation, mental disorder, and hopelessness | 1. No use of any healthy age- and sex-matched controls 2. No considerations of any use of medication that might influence non-motor symptoms 3. Cohort comprised of patients who were relatively cognitively intact limiting the scope of disease severity |
| **Defazio et al., (2017) [115]**    Multistates, Italy  Cross-sectional, observational study | iPD | N = 321 (68.3 ± 9.2)  M = 190  F = 131 | **Demographics and clinical assessments:**   1. Standardized interview on patients’ demographic/clinical data (i.e., age, sex, age at PD onset, antiparkinsonian therapy (LEDD) H&Y) 2. Pain quality: Dystonic pain, musculoskeletal pain, peripheral neuropathic pain, central neuropathic pain 3. Motor complications: Fluctuations/dystonia/dyskinesia 4. Any medical conditions predisposing to pain: e.g., diabetes, osteoporosis 5. Non-motor symptoms: NMS scale in nine domains | 1. 56% of PD patients reported chronic pain, either being muscular or arthralgic pain 2. Using a main effect model, female sex, medical conditions predisposing pain, motor complications, and NMS (sleep/fatigue and mood/cognition) are associated with pain 3. Pain in PD is more frequent in females | 1. Participants’ sample were limited to only high proportion of patients staging <3 on HY scale, a relatively short mean duration of follow-up and a low frequency of motor fluctuations (Only in early stage of PD) 2. No consideration of disease progression (no follow-up data) |
| **Perrin et al., (2017) [130]**  Vancouver, Canada  Cross-sectional, retrospective study | PD | N = 307  M = 190  F = 117 | 1. Patients’ records from tertiary movement disorders were reviewed and demographic data (i.e., age of motor onset symptoms, disease motor sub-type, disease severity (H&Y), BDI scores, MoCA scores and medications were extracted | 1. Recursive partitioning of males and females with depression relied on different key BDI items; melancholy featured prominently in females, while factors associated with PD depression (i.e., apathy and loss of libido) features prominently in males with PD | 1. Cross-sectional and retrospective nature of this study 2. Relatively small sample size 3. Other lifestyle and genetic factors that might affect experience of depression were not included |
| **Wang and Tickle-Degnen, (2018) [116]**  Boston, USA  Clinic-based cohort interview | iPD | N = 96 (66.46 ± 9.07)  M = 70 (66.8 ± 9.1)  F = 26 (65.54 ± 9.09) | **Clinical and neurophysiological tests:**   1. MMSE, 2. H&Y 3. GDS 4. Medications 5. Comorbidities   **Interview:**   1. 30-minutes videotaped interview protocol that involved self-reports about quality of life and enjoyable events in the past week, together with self-report measure of their experienced affect during interview   **Measures of emotional experience:**   1. PANAS 2. GDS 3. PDQ-39   **Measures of expressive behaviour:**   1. Interpersonal Communication Rating Protocol (ICRP) | 1. Females with more negative affect and depression appeared to smile and laugh more 2. A higher degree of expressive behaviour indicated more positive affect in PD patients 3. Males had more severe movements symptoms than females 4. Both sexes presented contrasting smiling and laughing behaviour when experiencing negative affect | 1. Sample did not include patients with severe depression – limited variation of depression 2. Relatively small sample size |
| **Picillo et al., (2019) [124]**  PRIAMO study, Italy  Prospective, longitudinal study | iPD | **Total at baseline:**  N = 355  M = 238 (M (with sexual activity) = 62.51 ± 9.77; M (without sexual activity) = 70.84 ± 7.24)  F = 117 (F (with sexual activity) = 61.27 ± 8.57; F (without sexual activity) = 68.06 ± 6.48) | **Demographics and clinical assessments:**   1. Each patient underwent a baseline (T1) and two follow-ups at 12 months (T2) and 9-16 months after T2 (T3) 2. Semi-structured interview exploring 12 NMS symptoms 3. Semi-structured interview on sexual activity for the past 12 months gender-tailored 4. UPDRS-III 5. MMSE 6. Depression: HAMD 7. Quality of Life: PDQ-39, EQ-VAS | 1. Gastrointestinal symptoms and apathy were less likely to be associated with sexual activity in males 2. Sexual activity in males was associated with lower motor disability and lower depression score 3. No association was found in females | 1. Still lack of longer longitudinal observation that enables understanding the natural history of men with PD with active sexual life 2. Lack of healthy controls 3. Lack of assessment of NMS severity 4. Association with apathy and reduced sexual activity not well-defined 5. Selection bias: Only included patients with H&Y <2.5 |
| **Picillo et al., (2021) [117]**  PRIAMO study, Italy    Cross-sectional, longitudinal, prospective study | iPD | **Total at baseline:**  N = 385  M = 247 (M without constipation: 65.05 ± 9.57; M with constipation: 68.24 ± 8.96)  F = 138 (F without constipation: 65.83 ± 8.82; F with constipation: 67.92 ± 7.57) | **Demographics and clinical assessments:**   - - - 1. Each patient underwent a baseline (T1) and two follow-ups at 12 months (T2) and 9-16 months after T2 (T3)  1. Semi-structured interview exploring 12 NMS symptoms 2. Only patients reporting gastrointestinal dysfunction in the semi structured interview were asked if they had developed constipation before the onset of motor symptoms 3. Motor disability: UPDRS-III 4. Cognition: MMSE 5. Depression: HAMD 6. Quality of Life: PDQ-39, EQ-VAS | 1. Prodromal constipation was highly associated with attention and memory complaints and apathy symptoms only in females 2. Prodromal constipation was associated with lower cognitive and higher apathy scores in older patients only | 1. Still lack of longer longitudinal observation that enables understanding the natural history of males and females with PD 2. Recall bias since presence of prodromal constipation was done with a patient interview only 3. Majority of non-motor were evaluated with a semi structured interview but not an objective interview 4. Sample drop/attrition at different stages 5. Lack of control group |
| **Anang et al., (2014) [123]**  Montreal, Canada  Prospective, longitudinal cohort study | iPD | N = 80 (66.2 ± 10.9)  M = 51  F = 29 | **Cognitive assessments:**   1. Executive function/attention: Digit Span from WAIS-III, TMT, Stroop Colour Word Test 2. Memory: Semantic and Letter verbal fluency and episodic verbal and learning memory 3. Visuospatial: Copy of the Rey-O figure, Block design from WAIS-III and Bells test   **Clinical assessments:**   1. Motor: UPDRS I-IV, H&Y, LEDD, Symmetry of onset, Purdue Pegboard Test, motor subtype (Schiess), axial-limb ratio, history of falls and freezing 2. Special sensory variables: Odour and colour discrimination 3. Autonomic variable: Blood pressure, symptoms of orthostatic dysfunction, urinary dysfunction, erectile dysfunction, and constipation 4. Sleep: RBD-PSG, ISI, ESS 5. Cognition: MCI, visual hallucination, depression, and apathy | 1. Patients destined to develop dementia were older and more often males (p = 0.023) 2. Those with baseline mild cognitive impairment had increased dementia risk 3. RBD at baseline dramatically increased dementia risk; however, neither daytime sleepiness nor insomnia predicted dementia | - - - 1. Severe dementia makes it difficult to confirm diagnosis       2. Size of cohort was still insufficient to examine markers with modest predictive value       3. Factors such as history of prior severe head injury, non-parkinsonian medication use, and family history of dementia were not included |
| **Raciti et al., (2020) [118]**  Multi-centre study, Italy  Cross-sectional study | PD | N = 203 (68.36 ± 8.5)  M = 113 (68.52 ± 8.65)  F = 90 (68.15 ± 8.35) | **Demographics, clinical and sexual assessments:**   1. UPDRS, HAM-D 2. Caregiver: CBI 3. Sexual function: Semi-structured interview, IIEF, FSFI and DIQ 4. Other demographics details: Semi-structured interview of 40-item | 1. 68% of males with PD and 53% of females with PD experience sexual dysfunction with loss of libido as the main sexual concern for both sexes 2. Males with PD were significantly more affected by sexual dysfunction than females with PD (p = 0.037), although no differences in severity of dysfunction was found | 1. Cross-sectional design of study 2. Relatively small sample size 3. No consideration of sexual body image to understand changes in sexual intercourse and sexual desire 4. Lack of control group 5. No use of endocrinology assessment |
| **Caplliure-Llopis et al., (2022) [259]**  Valencia, Spain  Cross-sectional, pilot study | PD | **PD:**  N = 21 (71.75)  M = 12  F = 9  **Controls:**  N = 30 (67.93)  M = 13  F = 17 | 1. Demographics such as height, weight and other comorbidities were obtained 2. Bone quality was assessed using calcaneal quantitative ultrasound (QUS). Parameters recorded were broadband ultrasound attenuation (BUA), imaging speed of sound (SOS), stiffness index (SI), T-score and bone density (BMD) | **Between controls and PD findings:**   1. T-score was lower in the PD group (p < 0.05) and SOS was higher in PD group (p < 0.05) 2. 28.6% of PD patients were osteoporotic with T-score values lower than -1.5 compared to 16.7% of osteoporotic individuals in the control group (p < 0.01)   **Between sexes:**   1. There were significant differences between females with PD and control group in the SI, T-score, BUA, and BMD (p < 0.05) with no difference in the comparison between the male groups 2. Comparison between both sexes in T-score only displayed significant differences for the PD group (p < 0.05), with worse bond quality in females | 1. Very small sample size 2. Data and groups were not stratified by time of incidence of the disease or stages of the disease 3. Other factors such as homocysteine levels or medications were not considered |
| **Cereda et al., (2016) [120]**  Milan, Italy  Cross sectional, retrospective study using Parkinson Institute-Milan (Italy) research database | iPD | N = 6599 (68.4 ± 10.1)  M = 3848 (67.8 ± 10.1)  F = 2751 (69.2 ± 10.1) | 1. Demographics, lifestyle information, general medical information, and disease specific records of all PD patients were obtained from database over a period of 18 years | 1. Male sex was an independent risk factor in developing dementia in PD 2. While the rate of dementia increased in males over all age strata, female prevalence began to increase steadily after the age of 65 years, reaching male estimates only after 80 years of age 3. Higher rates in male gender were observed between 60 and 80 years of age | 1. Samples captures institutionalised patients that are not commonly included in community-based studies 2. Large sample size allowed 5-year stratification for both age and PD duration that maintained sufficient statistical power even for disease duration than 20 years 3. Lack of comprehensive neuropsychological assessment |
| **Wee et al., (2016) [119]**  Singapore, Singapore  Prospective, longitudinal study | iPD | N = 89 (at baseline visit = 65.41 ± 7.86)  M = 65  F = 24 | **Demographics and Clinical Assessments:**   1. PD patients were assessed at baseline and at 6 months intervals for a period of 18 months 2. SAS 3. GDS, HADS-A, ESS, modified Barthel index 4. H&Y, UPDRS-III 5. MoCA 6. LEDD | 1. Significant levels of apathy were present in 42.7% of the sample at baseline with symptom severity remaining relatively stable on average over the course of 18 months 2. Male gender, lower educational attainment, higher depression symptom severity, more severe functional disability, and presence of dyskinesias predicted increasing apathy over the 18 months period | 1. Small sample size 2. Use of SAS only yields a global score and provides little information on the different dimensions of apathy 3. Use of self-reported scale underestimate symptoms in severely apathetic PD patients 4. Apathy and depression scale may overlap, hence need to be accounted |
| **Liu et al., (2015) [122]**  Pennsylvania, USA  Cross-sectional study using the PPMI database | Drug-naive PD | **PD:**  N = 414  M = 269 (62.1 ± 9.8)  F = 145 (60.6 ± 9.6)  **Controls:**  N = 188  M = 121 (61.7 ± 10.9)  F = 67 (59.6 ± 11.7) | **Demographics and Clinical assessments:**   1. Demographics, clinical assessment, and history were obtained from the PPMI database 2. UPDRS-III, H&Y, PD phenotypes 3. Sleep: ESS, RBDSQ 4. Olfactory: UPSIT 5. Neurobehavioral: STAI, Questionnaire for Impulsive-Compulsive Disorder, GDS 6. Autonomic: SCOPA-AUT 7. Neuropsychological: MoCA | 1. Males with PD had significantly more prominent deficits in olfaction (p = 0.02), and in certain cognitive measurements (all p < 0.01) than females with PD 2. Females with PD experienced higher trait anxiety (p = 0.02) 3. UPSIT, MoCA and SCOPA-AUT were most predictive NMS measurements in males as compared to UPSIT, MoCA, and RBDSQ in females | 1. Use of wide range of well-validated assessments 2. Cross-sectional nature of study 3. Sample comprises of predominantly white volunteers - selection bias |
| **Guo et al., (2013) [129]**  Sichuan, China  Cross-sectional, observational study | PD | N = 522 (61.6 ± 11.4)  M = 296 (63 ± 11.2)  F = 226 (59.7 ± 11.5) | **Demographics and Clinical assessments:**   1. Demographic features and clinical data including age, age of onset, gender, disease duration, and anti-parkinsonism medication were obtained using a standard questionnaire during face-to-face interviews 2. LEDD, UPDRS-III, H&Y, NMSS scale | 1. The sleep/fatigue domain, mood/apathy and “pain” symptoms were more prevalent and severe in females with PD while urinary symptoms were more common and severe in males with PD 2. Significant positive correlations were found between disease duration, H&Y stage, UPDRS-III and NMSS score in total sample, subgroups of both males and females with PD and early and late onset PD | 1. Cross-sectional nature of study - does not account for development of disease at different stages of disease 2. Samples are not generalizable to other populations |
| **Leentjens et al., (2013) [128]**  Multicenter, international study  Cross-sectional study | PD | N = 342 (64.8 ± 9.2)  M = 209  F = 133 | **Demographics and Clinical Assessments:**   1. Demographics and disease-related variables assessed during an unstructured clinical interview 2. UPDRS and H&Y 3. MMSE 4. IADL 5. HAMD   Structural equation modelling of direct and indirect associations of risk factors with the latent depression outcome | 1. Model with acceptable fit explained 41% of variance in depression 2. 3 PD-specified variables (increased disease duration, more severe motor symptoms and the use of levodopa) and 6 nonspecific variables (female sex, history of anxiety and/or depression, family history of depression, worse functioning on activities of daily living and worse cognitive status) were significantly associated with depression | 1. Other important factors of depression such as marital status, availability of caregiver was not included 2. It is difficult to separate markers that are related to PD and those that are not directly related to PD 3. Model requires confirmation in a more longitudinal design that includes more contextual variables |
| **Kang et al. (2022) [133]**  Gwangju, South Korea  Retrospective, cross-sectional study | PD | N = 415 (65.6 ± 9.5)  M = 201 (65.4 ± 10.2)  F = 214 (65.9 ± 8.9) | **Demographics and clinical assessments:**   - - - 1. Age, duration of PD, age of symptom onset, and formal education, anti-PD drug treatment was obtained via face-to-face interviews  1. Motor and non-motor symptoms: UPDRS-II, III, NMSS, MMSE, BDI, PSQI | - - - 1. Females with PD had significantly lower scores on the UPDRS-III and postural tremor compared to males with PD, after controlling for formal education. No gender differences were observed in scores related to other motor symptoms  1. Males with PD had higher scores of sexual functions on the NMSS, which means sexual dysfunction was more severe and occurred frequently in males with PD | - - - 1. Study did not include control group  1. This study is a retrospective study conducted in a single tertiary referral centre, thus there is a possibility that gender differences may be influenced by biases such as treatment and recruitment 2. Influence of dopaminergic drugs on motor and non-motor symptoms cannot be excluded |
| **Martinez-Martin et al. (2012) [3]**  Non-Motor International Longitudinal Study (NILS)  Longitudinal, cross-sectional study | PD | N = 950 (age onset = 56.43 ± 10.78)  M = 595 (age onset = 55.9 ± 10.59)  F = 355 (age onset = 57.33 ± 11.04) | **Demographics and clinical assessments:**   - - - 1. Demographics and history data were obtained       2. H&Y       3. NMSS Scale       4. Motor disability and complications: SCOPA-Motor Scale       5. CISI-PD | - - - 1. There were no significant gender differences in demographics and clinical characteristics       2. For the entire PD sample, the most prevalent symptoms were nocturia (64.88%) and fatigue (62.78%) and the most prevalent affected domains were sleep/fatigue (84.02%)       3. Fatigue, feelings of nervousness, sadness, constipation, restless legs, and pain were more common and severe in females       4. Daytime sleepiness, dribbling saliva, interest in sex and problems having sex were more prevalent and severe in males | - - - 1. The nature of cross-sectional design of this study       2. Potential selection bias as the sample came from clinical specialized units, and the lack of cognitive data       3. Study did not include healthy controls |
| **Kon et al. (2018) [135]**  Aomori, Japan  Longitudinal, retrospective study | PD | N = 148 (ICB+ = 68; ICB- = 71) | **Demographics and clinical assessments: two-year follow-up**   - - - 1. MMSE       2. QUIP       3. H&Y and UPDRS       4. Olfactory dysfunction: Assessed by interview       5. Dyskinesias       6. LEDD | The ICB remission rate was 60%, the ICB persistence ratio was 40%, and the ICB development ratio was 12.6% over 2 years  Statistically, younger age and pergolide use were associated with ICB persistence.  Being male, having dyskinesia, and rotigotine, entacapone, zonisamide, and istradefylline use were associated with ICB development. | This study was a retrospective observational study, so conclusions about the factors associated with the development of ICB may be tenuous  The QUIP assessment conducted by their patients or their caregivers and has some delicate questions, such as regarding sexual activity, so patients may feel uncomfortable completing it honestly  This study used a small sample size of PD patients  This study was conducted in a single centre and patients may not represent the general PD population |
| **Kovács et al. (2016) [138]**  Pécs, Hungary  Cross-sectional study | PD | N = 621 (66.9 ± 9.2)  M = 361  F = 260 | **Demographics and clinical assessments:**   - - - 1. Age at onset, duration of fluctuations, type of PD (i.e., tremor dominant or rigid akinetic) and LEDD were obtained       2. Motor symptoms: UPDRS, H&Y, dyskinesia       3. Non-motor symptoms: UPDRS, NMSS, ESS, PDSS-2, MADRS, LARS, HAMA, MMSE, MoCA, ACE       4. Quality of life: PDQ-39 | - - - 1. Although females received significantly lower dosage of levodopa, they had significantly more disabling dyskinesia and worse postural instability than males       2. Anxiety, pain, sleep disturbances and orthostatic symptoms were more frequent among females while sexual dysfunction, apathy and daytime sleepiness were more severe than males       3. Females had worse quality of life than males       4. Female sex is an independent predictor for having lower quality of life in PD | - - - 1. This study had a monocentre design instead of a more favourable multicentre one  1. Possibility of selection bias |
| **Interventions: Surgical (Deep Brain Stimulation, DBS)** | | | | | |
| **Chan et al. (2014) [169]**  Multicentre, USA  Retrospective cohort study | PD | Total PD discharge = 2408302 (77.53 ± 13.19)  Total PD discharge for DBS of total discharge = 18 312 (63.66 ± 11.3) | 1. Queried the Nationwide Inpatient Sample in combination with a neurologist and neurological surgeon countrywide density data from the Area Resource File 2. Analysed factors predicting DBS use in PD using hierarchical logistic regression analysis, including patient and hospital characteristics, including age, sex, and settings | 1. 4.7% of all PD discharges were African American, while only 0.1% of DBS for PD discharges were African American 2. Factors that predicted DBS use are younger age, male sex, increasing income quartile of patient zip code, large hospitals, teaching hospitals, urban settings, hospitals with a higher number of annual discharges for PD, and increased countywide density of neurologists (p < 0.05) 3. Predictors of non-use include African American race, Medicaid use and increasing comorbidity score (p < 0.001) | 1. This study is limited to the period 2002 to 2009 – the impact of disparity in DBS access for PD may be greater in the recent years 2. Retrospective nature of this study |
| **Chandran et al. (2014) [166]**  Kerala, India  Clinic-based cohort study | PD | N = 51  M = 32 (age at DBS = 55.8 ± 10.7)  F = 19 (age at DBS = 54.5 ± 10.7) | **Demographics/Clinical assessments:**   1. Patients were evaluated one month before and 12 months after STN DBS 2. UPDRS I-IV 3. LEDD 4. MMSE & ACE 5. PDQL | **Pre-operative characteristics:**   1. Lower doses of drugs (p = 0.03), worse emotional scores in PDQL (p = 0.01) and worse depression (p = 0.03) in females   **Post-operative:**   1. No gender differences in surgical outcomes, except a lesser reduction of dopaminergic drugs in females | 1. This study only included a relatively short duration of follow-up (one year) |
| **Chiou (2015) [173]**  Taiwan, China  Retrospective study | PD | N = 72 (age at DBS = 61.1 ± 2.3)  M = 48 (age at DBS = 60.5 ± 3)  F = 24 (age at DBS = 62.5 ± 3.7) | **Demographics and Clinical assessments:**   1. Patients were evaluated at baseline and six months after 2. UPDRS I-IV and H&Y 3. MMSE | **Pre-operative:**   1. Males and females with PD did not differ in clinical severity, but females had slightly worse cognition (p < 0.05) and better response to levodopa (p < 0.05)   **Post-operative:**   1. No sex differences were observed after STN-DBS therapy | 1. Retrospective nature of this study 2. Findings only limited till December 2013 |
| **Willis et al. (2014) [170]**  Washington, USA  Retrospective, cohort study | iPD | **No DBS:**  N = 657,345  M = 331,870  F = 329, 987  **With DBS:**  N = 8420  M = 4,996  F = 3,424 | **Demographics and clinical history:**   - - - 1. Patients’ comorbidities       2. Patients’ neighbourhood SES       3. Provider and PD patient population characteristics: i.e., physician speciality, outpatients encounter | - - - 1. Females have lower odds of receiving DBS compared with males  1. Greatest disparities associated with race: Black and Asian beneficiaries were considered less likely to receive DBS than white | - - - 1. Retrospective nature of this study |
| **Shpiner et al. (2019) [168]**  Miami, USA  Retrospective study | PD | N = 207  M = 157  F = 50 | 1. Demographics, clinical history, and disease characteristics were obtained from the University of Miami DBS database from January 2014 to December 2017. | 1. Of reasons of those who did not receive surgery, females with PD’s preference (28.0%) were statistically significant compared to males (12.2%) (p < 0.001), whilst males were more likely to be lost to follow-up (p = 0.046) 2. No significant difference in post-surgical outcomes was found | - - - 1. Small sample size       2. The analysis only limited to a single health system       3. Retrospective nature of this study       4. No consideration of factors such as clinical manifestations and quality of life |
| **Rocha et al. (2021) [175]**  Porto, Portugal  Retrospective study | PD | N = 346 (age at surgery = 60 ± 7)  M = 208  F = 138 | **Demographics and Clinical assessments:**  PD patients were assessed before and one month after surgery and then every six months (i.e., UPDRS I, II, IV, Schwab and England Scale, BDI-II, GDS)  **Neuropsychological assessments:**   1. Performed prior to surgery and post-operatively at six months, 18 months, and five years (i.e., MMSE, FAB, Clock drawing test, DRS, Verbal fluency, Stroop test, TmT | 1. Male sex and disease duration were the only predictors of mortality in this multivariate analysis | Retrospective nature of this study  Selection bias as analyses only conducted in a single centre  Absence of control group |
| **Roediger et al. (2019) [171]**  International, multicentre  Retrospective study | iPD | N = 158 (age at surgery = 58.7 ± 7.7) | **Demographics and clinical assessments:**   1. Data from video repositories in three specialized Movement Disorders Centres were obtained 2. Video assessments, medical records, and clinical assessments (i.e., UPDRS)   **Video frame extraction and marker placement for posture analysis:**  A computerized analysis of posture was used to quantify the thoracolumbar, thoracic, and cervical-occipital ventral angles, as well as the thoracolumbar and cervical-occipital lateral angles from the video  Data were extracted from frames from video recordings in the pre-surgical medication-ON (dopamine therapy) and post-surgical stimulation-ON/medication-ON states (STN DBS plus dopaminergic therapy) | There was a 6.7% amelioration in the global postural angle between the pre- and post-surgical assessments (p = 0.031).  Motor response to and pre-surgical dosage of levodopa, male gender, and shorter PD duration were identified as predictors for posture improvement after STN DBS | There is a lack of information relating to the duration of posture abnormalities, which may play a crucial role in the outcome of STN DBS  There was a lack of data on the long-term posture effect of STN DBS  There was a lack of patient-centred measures of functional impairment, such as health-related quality of life |
| **Martinez-Ramirez et al. (2014) [151]**  Florida, USA  Retrospective study | PD | N = 345  M = 239  F = 106 | A retrospective chart review of PD patients over a period of 6 months was obtained  Demographics and clinical assessments reports (i.e., treatment doses, UPDRS, H&Y, PDQ-39)  BR patients were defined as taking 100mg or less of levodopa per dose and were required to report symptoms of disabling dyskinesia in a more recent visits before DBS surgery | There were significantly more females in BR group (58%) compared to 29% in the NBR group (p = 0.008)  BR patients had lower mean weight (p < 0.001), longer mean disease duration (p = 0.003), longer time on LD (p = 0.001) and higher UPDRS scores (p = 0.001)  63% of BR group had undergone DBS compared to 18% (p = 0.001). Dyskinesia was more severe and common and often painful in the BR group (p < 0.001) | Cross-sectional and retrospective nature of the study  Selection bias as study sample may have been more geared to severely affected patients |
| **Kim et al. (2019) [176]**  Seoul, Korea  Retrospective study | PD | N = 100  M = 48 (age at surgery = 57.3 ± 8.5)  F = 52 (age at surgery = 60.2 ± 6.7) | Records of PD patients with 5-year follow-up data were obtained between 2005 and 2013  Postoperative evaluations were performed 3, 6, 12 months and then yearly after STN-DBS  Clinical assessments were conducted at baseline (before surgery) and 1- and 5-year postoperative follow-up data: SF-36, PCS | There was an improvement in the PCS scores in both males (p < 0.001) and females (p = 0.001) at 1-year follow-up as compared to baseline  At 5-year follow-up, males had greater improvement in PCS scores (p < 0.001) but not in females (p = 0.409) compared with baseline | Small sample size  Some more validated measures of symptoms, such as other non-motor symptoms, could be included |
| **Jost et al. (2022) [165]**  Longitudinal data from prospective, observational, multicenter international NILS study | PD | N = 505  M = 335  F = 170 | Cross-sectional cohort: Gender proportions at referral, indication evaluations and DBS surgery were obtained  Longitudinal cohort: Clinical assessments at preoperative baseline and 6-month follow-up after surgery (i.e., LEDD, NMSScale) | Females with PD were disproportionately underrepresented in referrals as compared to the general population (p = 0.002) but more likely to be approved for DBS than males (p = 0.029)  For females with PD, the total relative risk of undergoing DBS was 0.74 compared to males with PD  At baseline, females with PD had longer disease duration, worse dyskinesia, worse mobility, and bodily discomfort. At follow-up, all main outcomes improved equally in both genders | In the cross-sectional cohort, the reasons why patients did not receive DBS were analysed retrospectively and the reasons for DBS referrals were not assessed systematically  This study only used a relatively small sample size of the two cohorts  A short time of 6 months for the evaluation of the outcome of DBS in PD patients |
| **Hariz et al. (2013) [177]**  Umeä, Sweden  Clinic-based cohort study | PD | N = 49  M = 31 (57.7 ± 7.8)  F = 18 (57.6 ± 6.6) | Patients were assessed pre- and post-operatively after DBS with a series of clinical assessments such as:  Quality of life: PDQ-39  Motor and non-motor symptoms: UPDRS I-IV  Medications: LEDD | Duration of disease at surgery and off-medication scores of the motor part of the UPDRS were similar in females and males with PD  At baseline, females had lower doses of dopaminergic medication than males, experienced more disability due to dyskinesias, had more sensory symptoms, and perceived more difficulties in mobility  Following DBS, both sexes display equal and significant improvements in off-medication scores on the UPDRS-III  On the PDQ-39, females expressed improvement in ADL to a greater extent than males. Moreover, females showed a positive effect on mobility, stigma, and cognition, as well as on the summary score of PDQ-39 | Small sample size  No consideration of other factors that might influence treatment outcomes, such as lifestyle factors |
| **Hamberg and Hariz (2014) [172]**  Umeä, Sweden  Qualitative study | PD | N = 39 (64.1 ± 8.2)  M = 31  F = 8 | Data were collected through qualitative interviews performed face-to-face in patients’ homes or at the clinic, or by telephone  Interviews were thematically structured with open-ended questions concerning broad areas in relation to PD and its treatment (i.e., course of disease before surgery, considerations about the surgery, the operations, symptoms, and life with PD)  Interviews were analysed according to the constant comparison technique in grounded theory (Qualitative analysis) | Three different approaches to DBS were identified among patients: (1) ‘Taking own initiative’; (2) ‘Agreeing when offered’; (3) ‘Hesitating and waiting’.   1. A higher proportion of females expressed strong fear of complications, and more females consulted friends and relatives prior to deciding about DBS | No consideration of cultural factors that might influence patients’ decision-making process  Small sample size |
| **Golfrè Andreasi et al. (2022) [174]**  Milan, Italy  Retrospective study | PD | N = 107 (age onset = 43.35 ± 7.97)  M = 71 (age onset = 43.38 ± 8.38)  F = 36 (age onset = 43.28 ± 7.22) | Patients’ demographics and clinical data were retrieved from the electronic database at baseline (less than 3 months before surgery) and at three follow-up visits (± 2 months, 5 ± 1 years, 10 ± 2 years).  **Pre-operative assessments:**  UPDRS-III in OFF and ON state  **Each follow-up visit:**  UPDRS-III & H&Y  LEDD  Dyskinesias: Anamnestic interviews by clinicians  Total Electrical Energy Delivered (TEED)  Adverse events | Females showed a trend towards worsening in bradykinesia already at 1-year follow-up and poorer scores in non-dopaminergic features at 10-year follow-up  LEDD was significantly reduced after surgery compared to baseline values; however, in males, LEDD remained significantly lower than baseline even 10 years after surgery, in females LEDD returned to baseline values  Males showed a sustained effect on dyskinesias, but this benefit was less clear in females  The total electrical energy delivered was consistently lower in females compared to males  Profile of adverse events did not appear to be influenced by sex. | Retrospective nature of this study and lack of collection of specific clinical scales on dyskinesias and wearing-off (only inferred from outpatients’ visits)  Absence of validated scales for measuring the quality of life and activities of daily living, and lack of comprehensive non-motor outcome variables, including cognitive performance |
| **Dalrymple et al. (2019) [167]**  Virginia, USA  Retrospective, cohort study | PD | N = 137 (63.1 ± 7.8)  M = 95  F = 42 | **Demographics and clinical data:**  Patients’ characteristics were obtained from a clinical database and chart review  Patients were assigned to the following groups: medication refractory tremor, motor fluctuations (with or without dyskinesias), motor fluctuations and tremor (when neither could be determined to be the primary indication), adverse effects of medication and dystonia  UPDRS-I, II, III, MoCA, TMT A&B, COWA, Semantic Fluency Test (animals), BDI-II, PDQ-39 | 1. 93.5% of PD patients who underwent DBS for medication refractory tremors were males, and 62.3% of PD patients who underwent DBS for motor fluctuations were males (p = 0.001). | Retrospective cohort study of patients accumulated from a single centre increased likelihood of missing data and selection bias  Regional variation in clinician referral patterns and patient preference may influence findings |
| **Interventions: Pharmacological** | | | | | |
| **Conti et al. (2022) [156]**  Salerno, Italy  Multicentric study | PD | N = 35  M = 19 (61 ± 8.7)  F = 16 (62 ± 11.8) | Patients were LD-naïve and received a single dose of LD/benserazide formulation  Fasting blood samples were collected before drug intake and then at 8-time points until 260 mins to measure pharmacokinetic parameters  Ultra-high-performance liquid chromatography coupled with mass spectrometry (UHPLC-MS) was used to measure LD concentrations | Area under curve (AUC) and maximum plasma concentration (Cmax) were significantly higher in females with PD than males with PD; (p = 0.0006 and p = 0.0014, respectively)  Female sex (p < 0.0001) and BMI (p = 0.014) significantly predicted AUC  Only female sex significantly predicted Cmax (p = 0.001) | Small sample size  No consideration of other factor that could affect drug mechanisms such as age, and other comorbidities |
| **Umeh et al. (2014) [260]**  USA, Canada  Large multicentre study | PD | N = 1,741  M = 1123 (age at diagnosis = 60.5 ± 9.4)  F = 618 (age diagnosis = 59.8 ± 10.1) | 1. Baseline data obtained from 45 participating sites, within five years of PD diagnosis 2. Type of dopaminergic medication and levodopa daily dose at baseline were measured 3. Chi-square statistic and linear regression models were used for statistical analysis | 1. No statistically significant differences in the frequency of use of different types of dopaminergic medications at baseline between females and males with PD 2. Small statistically significant difference was observed in the median unadjusted levodopa equivalent daily dose at baseline between females (300mg) and males (325mg), however results were not significant after adjusting for disease duration, severity, and body weight | Did not include any measures of hormones  Other factors that could affect medication intake such as mechanisms and effectiveness were not included |
| **Schwarzschild et al. (2019) [161]**  Multi-centre, USA  Retrospective study | PD | N = 75  M = 34  F = 41 | **Demographics, interventions, dosing, and follow-up:**  Demographics, patients’ screening (i.e., PD patients not on antiparkinsonian drug therapy, serum urate levels below ~6 mg/dL)  Participants were randomized 1:1:1 to 3 treatment groups: (1) Placebo, (2) Inosine titrated to mildly elevate serum urate (to 6.1 – 7.0 mg/dL), and (3) inosine titrated to moderately elevate serum urate (to 7.1 – 8.0mg/dL)  Primary outcomes: Safety, tolerability, and efficacy for urate elevation  Secondary outcomes: Clinical outcomes based on serial measurements of parkinsonism (UPDRS subscales, nonmotor assessments) | Inosine produced an absolute increase in average serum urate from baseline that was 50% greater in females (3.0 mg / dL) than males (2.0 mg/ dL)  Only in females was CSF urate significantly greater on mild or moderate inosine (+87% and +98% respectively) than on placebo.  Females in the higher inosine dosing group showed a 7.0 UPDRS points/year lower rate of decline versus placebo (p = 0.01).  In females, slower rates of UPDRS change were associated with greater increases in serum urate (p = 0.001).  No significant associations were observed in males | There is a lack of a direct measure of oxidative damage as a biomarker of the potential to benefit from inosine treatment given a putative antioxidant mechanism of action  Relatively small sample size |
| **Pellecchia et al. (2021) [163]**  Multinational, multicentre, observational study | PD | N = 1610 (68.4 ± 9.7)  M = 994 (67.8 ± 9.7)  F = 616 (69.4 ± 9.4) | **Data and Clinical Measurements:**  Data on demographics, adverse events and motor complications were collected from medical charts and by interviewing patients at each study visit  Adverse events: Medical Dictionary for Regulatory Activities (MedDRA)  ADL, UPDRS-III, PIGD | Safinamide improved motor symptoms and motor complications (fluctuations and dyskinesias) in both sexes, with good safety profile and without requiring any change in the concomitant dopaminergic therapy  Significant improvements were observed in 46% of males and females for the UPDRS motor score and 43.5% of males versus 39.1% of females for the total UPDRS score | Retrospective design of this study  There is the lack of standardized scales or patients’ diaries assessing motor complications |
| **Nishikawa et al. (2020) [158]**  Tokyo, Japan  Retrospective, clinical study | PD | N = 220 (68.1 ± 8.9)  M = 112  F = 108 | **Pharmacokinetics of levodopa:**  Venous blood samples were obtained for measurement of the plasma levodopa concentrations before drug administration and at 15 min, 30 min, 1, 2, 3, and 4 hours after drug administration  **Measurement of levodopa concentration:**   1. Levodopa concentration was measured by high performance liquid chromatography (HPLC) analysis | 1. Age, female sex, DOPA decarboxylase inhibitor (DCI) and body weight were significantly related to area under curve (AUC) at 4 hours, while disease duration, dyskinesia status and eGFR were not related to AUC at 4 hours and Cmax. | This study did not perform longitudinal assessment of temporal changes in the AUC at 4 hours of individual patients  This study only analysed the pharmacokinetics of levodopa and did not fully evaluate the aspect of pharmacodynamics such as efficacy of levodopa |
| **Kumagai et al. (2014) [157]**  Tokyo, Japan  Clinical study | PD | N = 128 (77.9 ± 6.3)  M = 51  F = 77 | **Demographics, neuroimaging, clinical assessments, and levodopa pharmacokinetics:**  H&Y  [123I] PET  Patients were orally given tablets containing 100mg of L-dopa and 10mg of carbidopa  Blood samples were collected at 0, 15, 30, 60, 120, 180 minutes after L-dopa administration. After which, pharmacokinetics parameters were computed | The area under the curve (AUC) and the AUC adjusted for body weight were found to be significantly greater in the females compared to males (p <0.0001 and p <0.0001, respectively).  In elderly patients, the AUC and AUC adjusted for body weight were significantly greater among females (p < 0.0001 and p < 0.0001, respectively) | Relatively small sample size  This study does not account for other factors such as lifestyle and genetic factors |
| **Olanow et al. (2013) [159]**  Stalevo Reduction in Dyskinesia Evaluation in PD (STRIDE-PD) study  Prospective, multicentre, double-blind, randomized study | Early PD | N = 745  N (levodopa therapy with carbidopa, LC) = 372  N (levodopa therapy with entacapone, LCE) = 373 | Blinded assessments for dyskinesia and wearing-off were performed at 3-month intervals for the 134- to 208-week duration of the study  Patients were divided into 4 dose groups based on nominal L-dopa dose at the time of onset of dyskinesia (or at study conclusion if there was no dyskinesia): Group 1 (<400 mg/day), Group 2 (400mg/day), Group 3 (401 to 600 mg/day) and Group 4 (>600mg)  Outcome measures: times to onset and frequency of dyskinesias, wearing-off, or any motor complication | Factors that were predictive of dyskinesias, in rank order were: young age at onset, higher L-dopa dose, low body weight, North American geographic region, LCE treatment group, female sex, higher severity on the UPDRS-II scale  The risk of developing dyskinesia or wearing-off was closely linked to L-dopa dose | Study is one of the longest and largest prospective double-blind trials  Relatively small sample size  Other factors such as genetics were not considered |
| **Quality of Life** | | | | | |
| **Balash et al. (2019) [152]**  Tel-Aviv, Israel  Cross-sectional study | PD | N = 319 (68.3 ± 10.6)  M = 206  F = 113 | **Demographics and clinical assessments:**   1. PDQ-39: domains such as mobility, activities of daily living, emotional problems, stigmatisation, social support, communication problems etc 2. MCSI: burdens such as physical strain, social constraint, financial strain etc | **Patients:**   1. Female patients reported reduced QoL due to depression and pain 2. Worsening of QoL in advanced PD was reported only in male patients   **Caregivers:**   1. Female caregivers felt exhaustion and damage to their health resulting from care twice as often as male caregivers 2. Social constraints and time limitations were more frequent in female caregivers 3. With increasing disease severity, female caregivers reported excessive demands from their male spouses, increased | 1. The cross-sectional design of this study may result in bias due to selective survival 2. Examined PD cohort is not typical for a general PD population 3. Insufficient information about lifestyle factors of patients and caregivers, demographics, education and emotional state and other additional help |
| **Nwabuobi et al. (2019) [179]**  New York, USA  Retrospective, exploratory study | Homebound PD | N = 85 (median age at first visit = 79.6)  M = 41 (median age at first visit = 77.2)  F = 44 (median age at first visit = 79.4) | 1. Demographics and clinical history of patients from a centre, from February 2014 to July 2016, including data collected via in-person interviews at the time of each initial home visit, and subsequent chart review | 1. Males were significantly more likely to have a PD diagnosis than females (17.1 % vs 2.3%) 2. Females were more likely to live alone (18.1% of females had no caregiver vs 2.4% of males) | 1. Findings are from a convenience sample in New York, and may not be generalizable to a wide PD population 2. Only focuses on social aspects, no other factors that could influence sex differences 3. Small sample size |
| **Vlaanderen et al. (2019) [187]**  Nijmegen, Netherlands  Retrospective study | Newly diagnosed PD | N = 22,293  M = 13,518 (age at diagnosis = 71.6 ± 9.9)  F = 8,775 (age at diagnosis = 72.5 ± 10.2) | 1. Clinical and demographics of PD patients from 2012 and 2016 in a national administrative medical claims database 2. Time-to-event analysis was used to identify the moments when patients received care from neurologists, allied healthcare therapists or general practitioners. 3. Other clinical milestones: unexpected hospitalization for PD, pneumonia, orthopaedic injuries, nursing home admission and death | 1. Females significantly visited general practitioners and physiotherapists significantly earlier and more often than males 2. After five years, 37.9% of females had visited an occupational therapist and 18.5% a speech and language therapist at least once, and 33.1% and 23.7% | 1. This dataset contained all newly diagnosed patients in the country for the past five years, reducing a potential selection bias 2. This study sample most likely included people who were incorrectly diagnosed due to selection criteria 3. Highly standardised claims data 4. Findings might be difficult to extrapolate to other country with another organisation of the healthcare system |
| **Farhadi et al. (2017) [184]**  Tehran, Iran  Stockholm, Sweden  Observational, cross-sectional study | iPD | N = 157  M = 108 (61.4 ± 11.2)  F = 49 (60.5 ± 13.6) | **Demographics and clinical assessments:** face-to-face interview   1. Motor severity: UPDRS I-IV and H&Y 2. Motor Subtypes: PIGD and FOSS 3. Predominance of core manifestations: tremor, bradykinesia etc 4. Axial/Limb ratio 5. Presence of falls and freezing 6. Nonmotor symptoms: HADS, FSS, MNA, SCOPA-PS, PDQ-39, HRQoL | 1. No sex differences in UPDRS and a cumulative daily dose of levodopa 2. Females had significantly more severe anxiety, worse nutritional status and poorer QoL 3. Emotional well-being, bodily discomfort, social support, mobility, and communication domains of HRQoL, together with anxiety, depression and psychosocial functioning, were the strongest features with more severe/worse status in females after adjusting for confounders | 1. Cross-sectional research restricts any causal inferences and going beyond associations 2. Other aspects of sexual dimorphism that need to be accounted for (i.e., progression, mortality) 3. Selection bias as patients was from an outpatient neurology clinic where most patients had mild-to-moderate iPD |
| **Fullard et al. (2018) [182]**  Philadelphia, USA  Retrospective, cohort study | Newly diagnosed PD | N = 133,133  M = 62, 675  F = 70, 458 | 1. Dataset from Medicare beneficiaries diagnosed with PD in 2002 through 2008 2. Prevalence and cumulative incidence of common medical conditions, trends in survival and health care utilisation of males and females with PD were computed | 1. Females with PD had significantly higher adjusted incidence rate ratio (IRR) of depression, osteoporosis, and rheumatoid/osteoarthritis than males 2. Despite higher survival, females with PD used home health and skilled nursing facility care more often and had less outpatient physician contact than males throughout the period | 1. Large sample size 2. The cohort study population may not be representative of the general population |
| **Meng et al. (2022) [180]**  Beijing, China  Cross-sectional study | PD | N = 162  M = 70 (60.41 ± 9.23)  F = 92 (59.6 ± 7.24) | **Clinical Assessments:**   1. UPDRS I-IV and H&Y 2. MoCA 3. HAMD and HAMA 4. RBDSQ 5. PFS-16 6. PDSS 7. HRS 8. MAES 9. QUIP 10. PAC-QOL and PDQ-39 | 1. Significant differences between genders were found in levodopa equivalent, Hamilton Anxiety Rating Score, RBDSQ, HRS 2. After baseline imbalance corrections, a significantly higher score of PDQ-39 in females than in males 3. Females PD patients have poorer QoL than male patients, especially in bodily discomfort, stigma and emotional well-being | 1. Only included PD patients rated between 1 and 3 on the H&Y stage - selection bias 2. This study did not include the economic status of PD patients, which affects QoL 3. Small sample size |
| **Yoon et al. (2017) [186]**  Seoul, South Korea  Retrospective, case-control study | PD | **PD:**  N = 89  M = 47 (68.18 ± 8.14)  F = 42 (68.9 ± 7.71)  **Controls:**  N = 36 (65.2 ± 10.76) | **Demographics and clinical assessments:**   - - - 1. Clinical history       2. Quality of Life: HRQoL       3. BAI | 1. Total PD QoL and BAI scores were higher in female than in male patients 2. No association was found between non-motor symptoms and HRQoL in male patients 3. HRQoL was higher correlated with depression and moderately associated with fatigue in female patients | 1. Small sample size |
| **Sperens et al. (2020) [178]**  Part of a population- based project, the NYPUM project, New Parkinsonism in UMea)  Retrospective, exploratory study | PD | N = 129 (age at diagnosis = 70.5 ± 10)  M = 76 (age at diagnosis = 70.5 ± 10.2)  F = 53 (age at diagnosis = 70.5 ± 9.8) | **Demographics and clinical assessment:**   - - - 1. Collected at baseline and 12, 36, 60 and 96 months  1. Clinical assessments: UPDRS-III, H&Y, LEDD, MMSE, MADRS, ADL Taxonomy | 1. 9 out of 12 domains in ADL taxonomy showed a significant change over time (Eating and Drinking), mobility, toilet activities, dressing, personal hygiene, communication, cooking and cleaning 2. Two domains, shopping and cleaning, showed an effect of gender, with worse scores in females | 1. Longitudinal clinical studies are biased, with a greater dropout of older and ill patients 2. Small sample size |
| **Dahodwala et al. (2016) [185]**  National Parkinson Foundation (NPF) Parkinson’s Outcome Project  Longitudinal, observational study | iPD | N = 4,679  M = 2,938 (65.5 ± 9.7)  F = 1,741 (66.9 ± 9.7) | **Clinical assessments:** at enrolment and after one year   - - - 1. Demographics, comorbidities, medications, disease duration  1. H&Y 2. Cognition: Five-word recall and verbal fluency, and Timed Up and Go (TUG) test score 3. 4. Quality of life: PDQ-39 | - - - 1. At baseline, females were significantly more likely to be older and have a higher disease severity and more comorbidities than males, which corresponds to worse function on the PDQ-39 and Timed Up and Go (TUG) test but not to the number of medications or cognitive functions.  1. After one year, declines across all functional measures except delayed recall occurred. There were no significant changes in PDQ-39, TUG, number of medications, or verbal fluency between the sexes | - - - 1. Large sample size, although there is still the possibility of selection bias among those who chose not to participate in the registry or were lost to follow-up  1. No consideration of other factors such as socioeconomic status, such as education or income, that might influence the relationship between sex and outcomes |
| **Abraham et al. (2019) [183]**  Maryland, USA  Longitudinal, clinical cohort study | PD | N = 1463  M = 914 (age at diagnosis = 63.1 ± 10.19)  F = 549 (age at diagnosis = 63.4 ± 11 | **Demographics and clinical history:**   - - - 1. Sociodemographic and clinical characteristics, treatment, care timing   **Outcomes:**   - - - 1. Impairment: physician reported UPDRS motor subscale score  1. Disability: Patient-reported disability using OARS-ADLs, OARS-IADLs and physician-reported disability using S&E 2. Health-related quality of life; patient-reported SF-12 3. Treatment complications: UPDRS Complications of Therapy 4. Psychological distress: BSI-18 5. Comorbidities: CIRS-G 6. Cognition: MMSE, MoCA | - - - 1. Females with PD had significantly less social support, more psychological distress, and worse self-reported (but not physician-reported) disability and HRQoL at initial PD care visits compared to males  1. PD progression sex differences were minimal | - - - 1. Misspecification of progression models  1. Certain information, such as medication dosage and some non-motor symptoms, were unavailable 2. Used database that includes extensive information on both physician- and patient-reported outcome measures 3. Use of mediation analysis able to provide insight into the reasons for sex differences in PD |
| **Iwaki et al. (2021) [181]**  International, 12 PD cohorts (North America, Europe, and Australia)  Cross-sectional, longitudinal study | PD | **Clinical cohorts:**  N = 5946  **Web-based, online cohort:**  N = 17719 | **Clinical outcome measurements:**   - - - 1. 23 outcome measures: 11 binomial and 9 continuous measurements  1. Binomial outcomes include constipation, mild cognitive impairments, depression, daytime sleepiness, hyposmia, insomnia, wearing-off, dyskinesia, and RBD. 2. 3. For continuous outcomes, H&Y, UPDRS, MMSE, MoCA and SEADL were used 3. Linear mixed-effects models to test sex-associated differences in progression were used | 1. Females with PD had a higher risk of developing dyskinesia early during the follow-up period, with a slower progression in activities of daily living difficulties and a lower risk of developing cognitive impairments, as compared with male patients | - - - 1. This study sample were mostly of European descent       2. No inclusion of environmental factors such as smoking, alcohol, and diet       3. No inclusion of sex hormones |

**Abbreviations:** **AAO**: Age at Onset; **ACE**: Addenbrooke’s Cognitive Examination; **ADAS-Cog**: Alzheimer’s Disease Assessment Scale-Cognitive; **ADL**: Activities of Daily Living; **BAI**: Beck Anxiety Inventory Score; **BDI-II**: Beck Depression Inventory-II; **BMI**: Body Mass Index; **BNT**: Boston Naming Test; **BSI-18**: Brief Symptom Inventory-18; **BTA**: Brief Test of Attention; **CBI**: Caregiver Burden Inventory; **CES-D**: Epidemiological Studies Depression Scales; **CIRS-G**: Cumulative Illness Rating Scale-Geriatric; **CISI-PD**: Clinical Impression of Severity Index of PD; **COWA**: Controlled Oral Word Association; **DBS**: Deep Brain Stimulation; **DRS**: Dementia Rating Scale; **EDS**: Excessive Daytime Sleepiness; **ESS**: Epworth Sleepiness Scale; **EQ-VAS**: EuroQoL Visual Analogue Scale; **F**: Female sample; **DIQ**: Diagnostic Impotence Questionnaire; **FAB**: Frontal Assessment Battery; **FOSS**: Freezing-Speech-Swallowing score; **FSFI**: Female Sexual Function Index; **FSS**: Fatigue Severity Scale; **GDS**: Geriatric Depression Scale; **HADS**: Hospital Anxiety and Depression Scale Questionnaire; **HAMA**: Hamilton Anxiety Rating Scale; **HAMD**: Hamilton Depression Rating Scale; **HRS**: Hyposmia Rating Scale; **HrQoL**: Health-related Quality of Life; **H&Y**: Hoehn and Yahr Scale; **HVLT-R**: Hopkins Verbal Learning Test;  **IIEF**: International Index of Erectile Function; **LARS**: Lille Apathy Scale; **L-dopa**: Levodopa; **LEDD**: Levodopa Equivalent Daily Dose; **M**: Male sample; **MADRS**: Montgomery-Asberg Depression Rating Scale; **ISI**: Insomnia Severity Index**; MAES**: Modified Apathy Evaluation Scale; **MCSI**: Multidimensional Caregiver Strain Index; **MCI**: Mild Cognitive Impairments; **MMSE**: Mini-Mental State Exam; **MNA**: Mini-Nutritional Assessment Questionnaire; **MoCA**: Montreal Cognitive Assessment; **MSLT**: Multiple Sleep Latency Test; **N**: Total number of samples; **NMSS**: Non-motor Symptom Scale; **NMS-Quest**: Non-motor Symptoms Questionnaire; **OARS-ADLs**: Older Americans Resource and Services Multidimensional Functional Assessment Questionnaire (OARS) Activities of Daily Living (ADLs); **OARS-IADLs**: Older Americans Resource and Services Multidimensional Functional Assessment Questionnaire (OARS) Instrumental Activities of Daily Living (IADLs); **PAC-QOL**: Patient Assessment of Constipation Quality of Life questionnaire; **PANAS**: Positive and Negative Affect Schedule; **PANDA**: Parkinson Neuropsychometric Assessment; **PCS**: Physical Component Summary; **PFS-16**: Parkinson Fatigue Scale; **PD**: Parkinson’s Disease; **PDSS**: Parkinson’s Disease Sleep Scale; **PDQ-39**: Parkinson’s Disease Questionnaire on Daily Living; **PLM**: Periodic Limb Movement; **PIGD**: Postural Instability-gait difficulty score; **PSQI**: Pittsburgh Sleep Quality Index; **QUIP**: Questionnaire for Impulsive-Compulsive disorders in Parkinson’s Disease; **RBD**: REM Behaviour Disorder; **RBDSQ**: REM Behaviour Sleep Disorder questionnaire; **SAPS**: Scale for Assessment of Positive Symptoms; **SAS**: Starkstein Apathy Scale; **SCOPA-AUT:** Scales for Outcomes in Parkinson’s Disease – Autonomic Questionnaire; **SCOPA-DS**: Scales for Outcomes in Parkinson’s Disease – Sleep Daytime Sleepiness; **SCOPA-Motor**: Scales for Outcomes in Parkinson’s Disease – Motor Questionnaire; **SCOPA-PS**: Scales for Outcomes in Parkinson’s Disease – Psychosocial Questionnaire; **SDMT**: Symbol Digit Modalities Test; **SF-12**: patient-reported 12 item Short Form Health Survey; **SF-36**: 36-Item Short Form Health Survey; **S&E**: Schwab and England Activities of Daily Living Scale; **SEADL**: modified Schwab and England Activities of Daily Living Scale; STAI: State-Trait Anxiety Inventory; **SSET:** Sniffin’ Sticks Extended test; **STN**: Subthalamic Nucleus; **TMT**: Trail Making Test; **UPDRS**: Unified PD Rating Scale; **UPSIT**: University of Pennsylvania Smell Identification Test; **WAIS**: Wechsler Adult Intelligence Scale; **WOQ-19**: 19-item Wearing Off-Questionnaire

*SS1.1 Methodological quality assessment results*

Comprehensive tables of the methodological quality assessment of studies using the EPHPP Quality Assessment Tool for Quantitative Studies [15] or the AMSTAR-2 [16] is displayed in the supplementary section*.* For studies assessed using the EPHPP scale, selection bias was generally rated ‘moderate’ as most study participants were sampled from outpatient settings such as movement disorder clinics. Study design was generally rated as ‘weak’ as most were cross-sectional. Most studies controlled for at least 80% of confounding variables, such as age and disease severity, and were rated as ‘strong’. Blinding was rated as ‘moderate’ if it was not described, as in almost all studies. In data collection, although most studies used well-validated and reliable measures of alpha-synucleinopathies, many studies had small sample sizes and other limiting factors, and were, therefore, rated ‘weak’. Where applicable, withdrawals were reported ‘satisfactorily’.

As a general score on the AMSTAR-2 scale is not recommended [16], Supplementary Table 2 only shows the results of all domains. None of the studies adhere to a well-designed protocol (Question 2, Q2). None used a comprehensive literature search strategy (Q4), although most studies reported some features of a comprehensive literature search (Q4). Only two studies included a list of excluded studies and justified their exclusions (Q7), while the results of the remaining studies were mixed. Half the reviews failed to use a satisfactory technique for assessing the risk of bias in individual studies, while the other half partially fulfilled this condition (Q9). Six of the studies included a meta-analysis, and these studies used appropriate methods for the statistical combinations of results (Q11). Six studies reported for results of risk of bias (RoB) assessments (Q13). Finally, four studies performed publication bias analyses and discussed its impact (Q15).

**SS Table 2:** Methodological evaluation of studies using the EPHPP Quality Assessment Tool for Quantitative Studies

| **Author and Year** | **Disease** | **Selection Bias** | **Study Design** | **Confounders** | **Blinding** | **Data Collection** | **Withdrawals and Drop-outs** | **Global rating** |
| --- | --- | --- | --- | --- | --- | --- | --- | --- |
| **Takeuchi et al. (2020) [25]** | RBD | Moderate | Weak | Strong | Moderate | Moderate | NA | **Moderate** |
| **Castelnuovo et al. (2020) [26]** | RBD | Weak | Weak | Strong | Moderate | Moderate | NA | **Weak** |
| **Bugalho & Salavisa (2019) [31]** | RBD | Moderate | Weak | Moderate | Moderate | Moderate | NA | **Moderate** |
| **Fernández-Arcos et al. (2016) [34]** | RBD | Moderate | Weak | Strong | Moderate | Moderate | NA | **Moderate** |
| **Zhou et al. (2015) [9]** | RBD | Moderate | Weak | Strong | Moderate | Moderate | NA | **Moderate** |
| **Brakedal et al. (2022) [43]** | PD | Weak | Weak | Strong | Moderate | Moderate | NA | **Weak** |
| **de Lau et al. (2014) [45]** | PD | Moderate | Moderate | Strong | Moderate | Moderate | Moderate | **Strong** |
| **Moisan et al. (2016) [48]** | PD | Moderate | Weak | Strong | Moderate | Moderate | NA | **Moderate** |
| **Pinter et al. (2015) [46]** | PD | Moderate | Moderate | Strong | Moderate | Moderate | Strong | **Strong** |
| **Savica et al. (2018) [50]** | PD | Moderate | Weak | Strong | Moderate | Moderate | NA | **Moderate** |
| **Ulivelli et al. (2022) [44]** | PD | Moderate | Weak | Moderate | Moderate | Weak | NA | **Weak** |
| **Georgiou et al. (2017) [56]** | PD | Strong | Moderate | Strong | Strong | Strong | NA | **Strong** |
| **Sampaio et al. (2018) [61]** | PD | Strong | Moderate | Strong | Strong | Strong | NA | **Strong** |
| **Cilia et al. (2014) [52]** | PD | Moderate | Moderate | Strong | Strong | Moderate | NA | **Strong** |
| **Gatt et al. (2013) [57]** | PD | Strong | Moderate | Strong | Strong | Moderate | NA | **Strong** |
| **Gusdon et al. (2015) [58]** | PD | Moderate | Moderate | Strong | Strong | Strong | NA | **Strong** |
| **Liu et al. (2014) [66]** | PD | Moderate | Moderate | Strong | Strong | Moderate | NA | **Strong** |
| **Mariani et al. (2016) [254]** | PD | Moderate | Moderate | Moderate | Strong | Moderate | NA | **Strong** |
| **Zhang et al. (2014) [64]** | PD | Moderate | Moderate | Strong | Strong | Strong | NA | **Strong** |
| **Zhao et al. (2015) [62]** | PD | Moderate | Moderate | Strong | Strong | Moderate | NA | **Strong** |
| **Ping et al. (2018) [63]** | PD | Moderate | Moderate | Strong | Moderate | Strong | Strong | **Strong** |
| **Cui et al. (2021) [55]** | PD | Moderate | Moderate | Moderate | Moderate | Strong | Moderate | **Strong** |
| **Lee et al. (2018) [67]** | PD | Weak | Weak | Moderate | Moderate | Strong | NA | **Weak** |
| **Baik et al. (2020) [68]** | PD | Weak | Weak | Strong | Moderate | Strong | NA | **Weak** |
| **Seyfried et al. (2018) [255]** | PD | Moderate | Moderate | Moderate | Moderate | Moderate | NA | **Strong** |
| **Bakeberg et al. (2019) [75]** | PD | Moderate | Weak | Moderate | Moderate | Moderate | NA | **Moderate** |
| **Wu et al. (2020) [76]** | PD | Weak | Weak | Strong | Moderate | Strong | NA | **Weak** |
| **Tremblay et al. (2020) [94]** | PD | Weak | Weak | Strong | Strong | Strong | NA | **Weak** |
| **Caranci et al. (2013) [82]** | PD | Moderate | Weak | Strong | Strong | Strong | NA | **Moderate** |
| **Gao et al. (2016) [69]** | PD | Moderate | Moderate | Moderate | Moderate | Strong | NA | **Strong** |
| **Ho et al. (2014) [83]** | PD | Moderate | Weak | Strong | Moderate | Strong | NA | **Moderate** |
| **Jesus et al. (2013) [70]** | PD | Moderate | Weak | Moderate | Moderate | Strong | NA | **Moderate** |
| **McFarland et al. (2013) [71]** | PD | Moderate | Moderate | Moderate | Moderate | Strong | NA | **Strong** |
| **Hamid et al. (2019) [81]** | PD | Moderate | Weak | Strong | Moderate | Strong | NA | **Moderate** |
| **Zhang et al. (2012) [72]** | PD | Moderate | Weak | Strong | Moderate | Strong | NA | **Moderate** |
| **Huang et al. (2021) [84]** | PD | Weak | Weak | Strong | Strong | Moderate | NA | **Weak** |
| **Zhang et al. (2022) [77]** | PD | Moderate | Weak | Strong | Moderate | Moderate | NA | **Moderate** |
| **Meoni et al. (2022) [80]** | PD | Moderate | Weak | Strong | Strong | Strong | NA | **Moderate** |
| **Luca et al. (2022) [78]** | PD | Moderate | Weak | Moderate | Moderate | Strong | NA | **Moderate** |
| **Bakeberg et al. (2021) [79]** | PD | Moderate | Weak | Weak | Moderate | Moderate | NA | **Weak** |
| **Baldini et al. (2020) [85]** | PD | Moderate | Weak | Moderate | Moderate | Strong | NA | **Moderate** |
| **Houser et al. (2018) [88]** | PD | Moderate | Moderate | Moderate | Moderate | Strong | NA | **Strong** |
| **Nissen et al. (2021) [87]** | PD | Moderate | Weak | Strong | Strong | Strong | NA | **Moderate** |
| **Nissen et al. (2022) [86]** | PD | Moderate | Weak | Moderate | Moderate | Strong | NA | **Moderate** |
| **Carlisle et al. (2021) [89]** | PD | Weak | Moderate | Moderate | Moderate | Strong | NA | **Moderate** |
| **Cortese et al. (2018) [74]** | PD | Moderate | Moderate | Moderate | Moderate | Weak | Strong | **Moderate** |
| **Lubomski et al. (2014) [96]** | PD | Moderate | Weak | Moderate | Moderate | Moderate | NA | **Moderate** |
| **Boccalini et al. (2022) [90]** | PD | Weak | Moderate | Strong | Moderate | Moderate | NA | **Moderate** |
| **De Micco et al. (2019) [92]** | PD | Weak | Moderate | Strong | Moderate | Strong | Strong | **Moderate** |
| **Porta et al. (2019) [91]** | PD | Weak | Weak | Moderate | Moderate | Moderate | NA | **Weak** |
| **Kolmancic et al. (2019) [93]** | PD | Weak | Moderate | Strong | Moderate | Moderate | Strong | **Moderate** |
| **Rusz et al. (2022) [256]** | PD | Weak | Weak | Strong | Moderate | Moderate | NA | **Weak** |
| **Bjornstad et al. (2016) [160]** | PD | Moderate | Moderate | Moderate | Moderate | Strong | Strong | **Strong** |
| **Colombo et al. (2015) [100]** | PD | Moderate | Weak | Weak | Moderate | Moderate | NA | **Weak** |
| **Cho et al. (2019) [257]** | PD | Weak | Weak | Strong | Moderate | Weak | NA | **Weak** |
| **Kim et al. (2018) [101]** | PD | Weak | Moderate | Moderate | Moderate | Strong | Strong | **Moderate** |
| **Ou et al. (2018) [102]** | PD | Weak | Moderate | Moderate | Moderate | Moderate | Moderate | **Moderate** |
| **Bakeberg et al. (2021) [42]** | PD | Moderate | Weak | Strong | Moderate | Moderate | Strong | **Moderate** |
| **Reekes et al. (2020) [103]** | PD | Weak | Moderate | Strong | Moderate | Moderate | Moderate | **Moderate** |
| **Cholerton et al. (2018) [121]** | PD | Moderate | Moderate | Moderate | Moderate | Moderate | Strong | **Strong** |
| **Gao et al. (2015) [131]** | PD | Moderate | Weak | Strong | Moderate | Strong | NA | **Moderate** |
| **Yang et al. (2018) [104]** | PD | Moderate | Weak | Strong | Moderate | Strong | NA | **Moderate** |
| **Heller et al. (2018) [105]** | PD | Moderate | Weak | Strong | Moderate | Moderate | NA | **Moderate** |
| **Kim et al. (2021) [65]** | PD | Weak | Moderate | Strong | Moderate | Moderate | Strong | **Moderate** |
| **Oltra et al. (2022) [107]** | PD | Weak | Weak | Strong | Moderate | Weak | NA | **Weak** |
| **Bayram et al. (2020) [106]** | PD | Moderate | Moderate | Strong | Moderate | Moderate | Moderate | **Strong** |
| **Oltra et al. (2022) [108]** | PD | Weak | Weak | Strong | Moderate | Strong | NA | **Weak** |
| **Song et al. (2014) [137]** | PD | Weak | Weak | Moderate | Moderate | Moderate | NA | **Weak** |
| **Picillo et al. (2022) [95]** | PD | Weak | Moderate | Strong | Moderate | Moderate | Weak | **Weak** |
| **Shin et al. (2017) [127]** | PD | Moderate | Weak | Moderate | Moderate | Moderate | NA | **Moderate** |
| **Fengler et al. (2016) [132]** | PD | Moderate | Weak | Strong | Moderate | Weak | NA | **Weak** |
| **Augustine et al. (2015) [258]** | PD | Weak | Weak | Strong | Moderate | Moderate | NA | **Weak** |
| **Szewczyk-Krolikowski et al. (2014) [126]** | PD | Moderate | Weak | Moderate | Moderate | Strong | NA | **Moderate** |
| **Pigott et al. (2015) [125]** | PD | Moderate | Moderate | Moderate | Moderate | Weak | Strong | **Moderate** |
| **Ratti et al. (2012) [110]** | PD | Weak | Weak | Moderate | Moderate | Moderate | NA | **Weak** |
| **Liu et al. (2021) [109]** | PD | Moderate | Weak | Moderate | Moderate | Moderate | NA | **Moderate** |
| **Bjørnarå et al. (2013) [139]** | PD | Weak | Moderate | Moderate | Moderate | Moderate | NA | **Moderate** |
| **Solla et al. (2020) [111]** | PD | Moderate | Weak | Strong | Moderate | Strong | NA | **Moderate** |
| **Nicoletti et al. (2017) [112]** | PD | Moderate | Moderate | Weak | Moderate | Strong | Strong | **Moderate** |
| **Hu et al. (2018) [113]** | PD | Weak | Weak | Moderate | Moderate | Moderate | NA | **Weak** |
| **Picillo et al. (2013) [114]** | PD | Weak | Weak | Strong | Moderate | Strong | NA | **Weak** |
| **Zhu et al. (2017) [136]** | PD | Moderate | Weak | Moderate | Moderate | Moderate | NA | **Moderate** |
| **Defazio et al. (2017) [115]** | PD | Moderate | Weak | Strong | Moderate | Moderate | NA | **Moderate** |
| **Perrin et al. (2017) [130]** | PD | Moderate | Weak | Moderate | Moderate | Moderate | NA | **Moderate** |
| **Wang and Tickle-Degnen (2018) [116]** | PD | Weak | Weak | Strong | Moderate | Moderate | NA | **Weak** |
| **Picillo et al. (2019) [124]** | PD | Weak | Moderate | Strong | Moderate | Moderate | Moderate | **Moderate** |
| **Picillo et al. (2021) [117]** | PD | Weak | Moderate | Moderate | Moderate | Moderate | Strong | **Moderate** |
| **Anang et al. (2014) [123]** | PD | Moderate | Moderate | Moderate | Moderate | Moderate | Strong | **Strong** |
| **Raciti et al. (2020) [118]** | PD | Weak | Weak | Strong | Moderate | Moderate | NA | **Weak** |
| **Caplliure-Llopis et al. (2022) [259]** | PD | Weak | Moderate | Strong | Moderate | Weak | Moderate | **Weak** |
| **Cereda et al. (2016) [120]** | PD | Moderate | Weak | Weak | Moderate | Strong | NA | **Weak** |
| **Wee et al. (2016) [119]** | PD | Moderate | Moderate | Moderate | Moderate | Weak | Strong | **Moderate** |
| **Liu et al. (2015) [122]** | PD | Weak | Weak | Weak | Moderate | Moderate | NA | **Weak** |
| **Guo et al. (2013) [129]** | PD | Moderate | Weak | Moderate | Moderate | Moderate | NA | **Moderate** |
| **Leentjens et al. (2013) [128]** | PD | Moderate | Weak | Strong | Moderate | Weak | NA | **Weak** |
| **Kang et al. (2022) [133]** | PD | Weak | Weak | Moderate | Moderate | Moderate | NA | **Weak** |
| **Martinez-Martin et al. (2012) [3]** | PD | Moderate | Moderate | Moderate | Moderate | Strong | NA | **Strong** |
| **Kon et al. (2018) [135]** | PD | Moderate | Moderate | Moderate | Moderate | Weak | NA | **Moderate** |
| **Kovács et al. (2016) [138]** | PD | Moderate | Moderate | Moderate | Moderate | Moderate | NA | **Strong** |
| **Chan et al. (2014) [169]** | PD | Moderate | Weak | Strong | Moderate | Moderate | NA | **Moderate** |
| **Chandran et al. (2014) [166]** | PD | Weak | Moderate | Weak | Moderate | Strong | Strong | **Weak** |
| **Chiou (2015) [173]** | PD | Weak | Weak | Strong | Moderate | Strong | NA | **Weak** |
| **Willis et al. (2014) [170]** | PD | Moderate | Moderate | Moderate | Moderate | Weak | Strong | **Moderate** |
| **Shpiner et al. (2019) [168]** | PD | Weak | Weak | Strong | Moderate | Moderate | NA | **Weak** |
| **Rocha et al. (2021) [175]** | PD | Weak | Weak | Strong | Moderate | Moderate | NA | **Weak** |
| **Roediger et al. (2019) [171]** | PD | Moderate | Moderate | Weak | Moderate | Moderate | NA | **Moderate** |
| **Martinez-Ramirez et al. (2014) [151]** | PD | Moderate | Weak | Strong | Moderate | Strong | NA | **Moderate** |
| **Kim et al. (2019) [176]** | PD | Weak | Medium | Strong | Moderate | Moderate | Strong | **Moderate** |
| **Jost et al. (2022) [165]** | PD | Weak | Weak | Moderate | Strong | Moderate | NA | **Weak** |
| **Hariz et al. (2013) [177]** | PD | Weak | Moderate | Moderate | Moderate | Moderate | Strong | **Moderate** |
| **Hamberg and Hariz (2014) [172]** | PD | Weak | Moderate | Moderate | Moderate | Moderate | Strong | **Moderate** |
| **Golfrè Andreasi et al. (2022) [174]** | PD | Weak | Moderate | Moderate | Moderate | Weak | Strong | **Weak** |
| **Dalrymple et al. (2019) [167]** | PD | Weak | Moderate | Strong | Moderate | Moderate | Strong | **Moderate** |
| **Conti et al. (2022) [156]** | PD | Weak | Moderate | Strong | Moderate | Strong | Strong | **Moderate** |
| **Umeh et al. (2014) [260]** | PD | Moderate | Weak | Strong | Moderate | Strong | NA | **Moderate** |
| **Schwarzschild et al. (2019) [161]** | PD | Weak | Weak | Moderate | Strong | Weak | NA | **Weak** |
| **Pellecchia et al. (2021) [163]** | PD | Weak | Moderate | Moderate | Moderate | Weak | Moderate | **Weak** |
| **Nishikawa et al. (2020) [158]** | PD | Weak | Moderate | Strong | Moderate | Moderate | Strong | **Moderate** |
| **Kumagai et al. (2014) [157]** | PD | Weak | Moderate | Moderate | Moderate | Moderate | NA | **Moderate** |
| **Olanow et al. (2013) [159]** | PD | Moderate | Moderate | Moderate | Moderate | Moderate | NA | **Strong** |
| **Balash et al. (2019) [152]** | PD | Moderate | Weak | Strong | Moderate | Moderate | NA | **Moderate** |
| **Nwabuobi et al. (2019) [179]** | PD | Weak | Moderate | Moderate | Moderate | Weak | Strong | **Weak** |
| **Vlaanderen et al. (2019) [187]** | PD | Moderate | Weak | Strong | Moderate | Moderate | NA | **Moderate** |
| **Farhadi et al. (2017) [184]** | PD | Moderate | Weak | Moderate | Moderate | Moderate | NA | **Moderate** |
| **Fullard et al. (2018) [182]** | PD | Moderate | Moderate | Strong | Moderate | Moderate | Moderate | **Strong** |
| **Meng et al. (2022) [180]** | PD | Weak | Weak | Strong | Moderate | Moderate | NA | **Weak** |
| **Yoon et al. (2017) [186]** | PD | Weak | Weak | Strong | Moderate | Strong | NA | **Weak** |
| **Sperens et al. (2020) [178]** | PD | Moderate | Moderate | Moderate | Moderate | Moderate | Strong | **Strong** |
| **Dahodwala et al. (2016) [185]** | PD | Strong | Moderate | Moderate | Moderate | Moderate | Strong | **Strong** |
| **Abraham et al. (2019) [183]** | PD | Moderate | Moderate | Moderate | Moderate | Weak | Moderate | **Moderate** |
| **Iwaki et al. (2021) [181]** | PD | Strong | Moderate | Moderate | Moderate | Moderate | NA | **Strong** |
| **Mouton et al. (2018) [193]** | DLB | Moderate | Weak | Strong | Moderate | Moderate | NA | **Moderate** |
| **Gan et al. (2021) [194]** | DLB | Moderate | Weak | Strong | Moderate | Moderate | NA | **Moderate** |
| **Savica et al. (2013) [195]** | DLB | Moderate | Moderate | Strong | Moderate | Moderate | NA | **Strong** |
| **Price et al. (2017) [196]** | DLB | Moderate | Moderate | Strong | Strong | Moderate | NA | **Strong** |
| **Boot et al. (2013) [197]** | DLB | Moderate | Moderate | Moderate | Moderate | Moderate | NA | **Moderate** |
| **Abdelnour et al. (2022) [211]** | DLB | Moderate | Moderate | Moderate | Moderate | Moderate | NA | **Strong** |
| **Gámez-Valero et al. (2016) [205]** | DLB | Weak | Moderate | Moderate | Moderate | Moderate | NA | **Moderate** |
| **Utsumi et al. (2020) [8]** | DLB | Moderate | Moderate | Strong | Moderate | Moderate | Strong | **Strong** |
| **Chiu et al. (2018) [208]** | DLB | Moderate | Weak | Strong | Moderate | Moderate | NA | **Moderate** |
| **Tsunoda et al. (2018) [207]** | DLB | Moderate | Moderate | Moderate | Moderate | Strong | Strong | **Strong** |
| **Bayram et al. (2021) [210]** | DLB | Moderate | Moderate | Strong | Moderate | Moderate | NA | **Strong** |
| **Choudhury et al. (2022) [209]** | DLB | Moderate | Weak | Moderate | Moderate | Moderate | Strong | **Moderate** |
| **Van de Beek et al. (2020) [213]** | DLB | Moderate | Weak | Moderate | Moderate | Weak | NA | **Weak** |
| **Ferreira et al. (2020) [204]** | DLB | Moderate | Weak | Moderate | Moderate | Strong | NA | **Moderate** |
| **Bayram et al. (2022) [203]** | DLB | Moderate | Weak | Moderate | Moderate | Moderate | NA | **Moderate** |
| **Sarro et al. (2017) [214]** | DLB | Moderate | Weak | Moderate | Moderate | Strong | Strong | **Moderate** |
| **Wennström et al. (2012) [215]** | DLB | Weak | Weak | Moderate | Moderate | Strong | NA | **Weak** |
| **Agbomi et al. (2022) [212]** | DLB | Moderate | Weak | Strong | Moderate | Strong | NA | **Moderate** |
| **Coon et al. (2019) [6]** | MSA | Moderate | Weak | Moderate | Moderate | Moderate | NA | **Moderate** |
| **Coon et al. (2015) [228]** | MSA | Moderate | Weak | Moderate | Moderate | Strong | NA | **Moderate** |
| **Cuoco et al. (2020) [233]** | MSA | Moderate | Moderate | Moderate | Moderate | Strong | Moderate | **Strong** |
| **Yamamoto et al. (2014) [7]** | MSA | Weak | Moderate | Moderate | Moderate | Moderate | Strong | **Moderate** |
| **Chen et al. (2015) [234]** | MSA | Moderate | Weak | Weak | Strong | Strong | NA | **Weak** |
| **Cao et al. (2013) [235]** | MSA | Moderate | Moderate | Strong | Strong | Strong | Moderate | **Strong** |

**SS Table 3:** Methodological quality assessment of included systematic reviews/meta-analyses using A MeaSurement Tool to Assess systematic Reviews- 2 (AMSTAR 2).

| **Question/Author and Year** | **Pringsheim et al. (2014) [49]** | **Xu et al. (2014) [47]** | **Gan-Or et al. (2015) [54]** | **Shu et al. (2018) [53]** | **Shen et al. (2013) [73]** | **Liu et al. (2022) [206]** | **Jones and O’Brien, (2014) [198]** |
| --- | --- | --- | --- | --- | --- | --- | --- |
| Q1. Did the research questions and inclusion criteria for the review include the components of PICO? | Yes | Yes | Yes | Yes | Yes | Yes | Yes |
| Q2. Did the report of the review contain an explicit statement that the review methods were established prior to the conduct of the review and did the report justify any significant deviations from the protocol? | No | No | No | No | No | No | No |
| Q3. Did the review authors explain their selection of the study designs for inclusion in the review? | Yes | Yes | Yes | Yes | Yes | Yes | Yes |
| Q4. Did the review authors use a comprehensive literature search strategy? | Partial Yes | Partial Yes | No | Partial Yes | Partial Yes | Partial Yes | No |
| Q5. Did the review authors perform study selection in duplicate? | Yes | No | No | Yes | Yes | No | Yes |
| Q6. Did the review authors perform data extraction in duplicate? | Yes | Yes | No | Yes | Yes | Yes | Yes |
| Q7. Did the review authors provide a list of excluded studies and justify the exclusions? | No | No | Yes | No | No | Yes | No |
| Q8. Did the review authors describe the included studies in adequate detail? | Partial Yes | Partial Yes | Yes | No | Yes | Yes | Partial Yes |
| Q9. Did the review authors use a satisfactory technique for assessing the risk of bias (RoB) in individual studies that were included in the review? | No | Partial Yes | No | No | Partial Yes | Partial Yes | Partial Yes |
| Q10. Did the review authors report on the sources of funding for the studies included in the review? | No | No | No | No | No | No | No |
| Q11. If meta-analysis was performed, did the review authors use appropriate methods for statistical combination of results? | Yes | No | Yes | No | Yes | Yes | No meta-analysis conducted |
| Q12. If meta-analysis was performed, did the review authors assess the potential impact of RoB in individual studies on the results of the meta-analysis or other evidence synthesis? | No | No | Yes | No | Yes | Yes | No meta-analysis conducted |
| Q13. Did the review authors account for RoB in primary studies when interpreting/discussing the results of the review? | Yes | Yes | Yes | No | Yes | Yes | Yes |
| Q14. Did the review authors provide a satisfactory explanation for, and discussion of, any heterogeneity observed in the results of the review? | Yes | Yes | Yes | Yes | Yes | Yes | Yes |
| Q15. If they performed quantitative synthesis did the review authors carry out an adequate investigation of publication bias (small study bias) and discuss its likely impact on the results of the review? | No | Yes | No | Yes | Yes | Yes | No meta-analysis conducted |
| Q16. Did the review authors report any potential sources of conflict of interest, including any funding they received for conducting the review? | Yes | Yes | Yes | Yes | Yes | Yes | Yes |
